# Supplementary material for: Longitudinal assessment of sputum microbiome by sequencing of the 16S rRNA gene in non-cystic fibrosis bronchiectasis patients
Source: PLoS One. 2017 Feb 7;12(2):e0170622. doi: 10.1371/journal.pone.0170622 (PMC5295668; doi:10.1371/journal.pone.0170622)
Supplement: S1 Appendix — (DOCX) [file pone.0170622.s001.docx]

**Supplementary methods**

**Quantitative PCR of 16S rRNA gene**

Quantitative PCR of each extracted DNA was performed in triplicate using the Viia7 Real Time PCR system (Life Technologies, Thermo Fisher Scientific, Waltham, MA, USA) and the primers 520 F AYT GGG YDT AAA GNG and 802 R TAC NVG GGT ATC TAA TCC targeting the 16S rRNA gene V4 region. One µl of DNA template was used in each 15 µl reaction consisting of 7.5 µl of KapaBio SYBR green Fast qPCR master mix (Qiagen, Venlo, Netherlands), 0.3 µl of each primer at 10 µM (0·2 µM final concentration) and 5·9 µl of analar water. Thermal cycling conditions were 90 ^o^C for 3 min followed by 40 cycles of 20 s at 95 ^o^C, 30 s at 50 ^o^C and 30 s at 72 ^o^C. This was performed with reference to a plasmid standard with known copy number between 10^4^ and 10^9^ copies (partial 16S rRNA gene of *Vibrio natriegens* DSMZ 759 (Deutsche Sammlung von Mikroorganismen, Braunschweig, Germany) from positions 27 to 1492 of the *Escherichia coli* reference cloned using TOPO TA cloning kit; Invitrogen, ThermoFisher Scientific). PCR efficiency was 65 and R^2^ of standard curve at least 0.99.

**16S rRNA gene sequencing and data processing**

16S rRNA gene amplification of the V3–V5 region was performed in quadruplicate using adapted primers 357F/926R (Sim *et al.*, 2012) with 12 bp barcodes included in the reverse primer (Fierer *et al.*, 2008) and 454 sequencing adaptors A and B included in the reverse and forward primers respectively. Quadruplicate reactions were combined, using Ampure XP (Ambion, ThermoFisher Scientific) and quantified using the QuantIT picogreen kit (Life Technologies, ThermoFisher Scientific). Purified amplicons were combined in three equi-molar pools. PCR negative controls, which gave no visible product on 1% agarose gels stained with GelRed (Biotium, Inc., Hayward, CA, USA) were also included in the sequencing as controls for any bacterial DNA introduced by the PCR reagents. The work was performed prior to routine DNA extraction control sequencing (Salter *et al.*, 2014). Three sequencing runs were performed on a Roche 454 FLX sequencer (454 Life Sciences, Branford, CT, USA) using the Titanium chemistry. Raw data was pre-processed on machine with software version 2·8 and using a custom version of the amplicon pipeline with parameters *valley trim back* and *qscore trim*: true and *vf scan all flows*: false thereby limiting OTU inflation and retaining additional reads.

All runs pre-processed raw data was then combined using Roche software and processed using AmpliconNoise and Perseus (Quince *et al.*, 2009) running in QIIME (Caporaso *et al.*, 2010) to limit the influence of sequence and PCR error on the results. Operational taxonomic units (OTUs) were picked using *de novo* OTU picking with UCLUST (Edgar, 2010) at 97% identity. The most abundant reads from each OTU were selected as reference sequences and identified using the RDP classifier (Wang *et al.*, 2007) retrained with the Silva 111 16S rRNA gene database release (Quast *et al.*, 2012). OTUs are named using the best taxonomic level for the identification and a unique number. A small number of reads were obtained from three of the six PCR negative controls. Additional OTUs known to be DNA kit contaminants in our lab (Salter *et al.*, 2014) were removed. The OTU table was randomly re-sampled to retain at least 451 reads per sample, with samples with fewer reads than this removed from the analysis. This was justified using examination of the sample ranked abundance curve (Fig S3) which indicated that this level retained the majority of samples, while also reasonably representing the species richness in each sample as demonstrated by the rarefaction curve (Fig S4). The resulting OTU table of 381 samples and 352 OTUs was used for all subsequent analyses and imported to the R statistical environment as a PhyloSeq (McMurdie and Holmes, 2013) object, along with a mid-point rooted FastTree phylogenetic tree. A cross-sectional dataset of 72 samples and 194 OTUs was produced by sub-sampling the first baseline sample available from each patient in PhyloSeq.

**PERMANOVA model**

The Adonis function from the Vegan package in R was used to test how much variation in beta-diversity could be explained by the test variables. Each variable was tested individually using the cross-sectional dataset and those that were significant retained for the final model. Adonis does not accept missing data and therefore samples were removed if they had missing data for variable being tested at the time. The variables affected and number of samples tested in each case of a possible 72 were: BMI, BMIClass(70), FEV1, FEV1pp, FEV50, FVC, FVCpp (68), Smoking (63), TotalExac (56), FirstPaer, and YrsPaer (39). There was no missing data for any other variable. The significant variables and their R^2^ values were: MucPaer 0.14437; Growth 0.07602; ColoPPx 0.07089; PrevPaer 0.05822; Saur 0.05718; PPxABx 0.03793; Hflu 0.006891. These variables were combined in a single model with the aim of maximising the variance explained and minimising the number of included variables. The sequence with which variables are input impacts the results, so the final variables were also tested in all possible (n = 24) sequences. The final model explained 29.1% of the variance and was:

adonis(bx.dist.cross.bray ~ MucPaer + Hflu + ColoPPx + Saur, as(sample_data(bx.cross), "data.frame"))

| Variable | Df | Sum of Squares | Mean of Squares | F model | R^2^ | Pr (>F) | Significance |
| --- | --- | --- | --- | --- | --- | --- | --- |
| MucPaer | 3 | 2.9842 | 0.99474 | 4.2764 | 0.14437 | 0.001 | *** |
| Hflu | 2 | 1.4111 | 0.70553 | 3.0331 | 0.06826 | 0.001 | *** |
| ColoPPx | 1 | 0.7240 | 0.72396 | 3.1123 | 0.03502 | 0.008 | ** |
| Saur | 2 | 0.8971 | 0.44856 | 1.9283 | 0.04340 | 0.013 | * |
| Residuals | 63 | 14.6457 | 0.23261 |  | 0.70895 |  |  |
| Total | 71 | 20.6710 |  |  | 1.00000 |  |  |

#Signif. codes: 0 ‘***’ 0.001 ‘**’ 0.01 ‘*’ 0.05 ‘.’ 0.1 ‘ ’ 1

Caporaso JG, Kuczynski J, Stombaugh J, Bittinger K, Bushman FD, Costello EK, *et al.* (2010). QIIME allows analysis of high-throughput community sequencing data. *Nat Meth* **7**: 335–336.

Edgar RC. (2010). Search and clustering orders of magnitude faster than BLAST. *Bioinformatics* **26**: 2460–2461.

Fierer N, Hamady M, Lauber CL, Knight R. (2008). The influence of sex, handedness, and washing on the diversity of hand surface bacteria. *Proceedings of the National Academy of Sciences* **105**: 17994–17999.

McMurdie PJ, Holmes S. (2013). phyloseq: An R Package for Reproducible Interactive Analysis and Graphics of Microbiome Census Data Watson M (ed). *PLoS ONE* **8**: e61217.

Quast C, Pruesse E, Yilmaz P, Gerken J, Schweer T, Yarza P, *et al.* (2012). The SILVA ribosomal RNA gene database project: improved data processing and web-based tools. *Nucleic Acids Research* **41**: D590–D596.

Quince C, Lanzén A, Curtis TP, Davenport RJ, Hall N, Head IM, *et al.* (2009). Accurate determination of microbial diversity from 454 pyrosequencing data. *Nat Meth* **6**: 639–641.

Salter SJ, Cox MJ, Turek EM, Calus ST, Cookson WO, Moffatt MF, *et al.* (2014). Reagent and laboratory contamination can critically impact sequence-based microbiome analyses. 1–12.

Sim K, Cox MJ, Wopereis H, Martin R, Knol J, Li M-S, *et al.* (2012). Improved Detection of Bifidobacteria with Optimised 16S rRNA-Gene Based Pyrosequencing Ahmed N (ed). *PLoS ONE* **7**: e32543.

Wang Q, Garrity GM, Tiedje JM, Cole JR. (2007). Naive Bayesian Classifier for Rapid Assignment of rRNA Sequences into the New Bacterial Taxonomy. *Applied and Environmental Microbiology* **73**: 5261–5267.
