# Supplementary figures and images for: Longitudinal assessment of sputum microbiome by sequencing of the 16S rRNA gene in non-cystic fibrosis bronchiectasis patients
Source: PLoS One. 2017 Feb 7;12(2):e0170622. doi: 10.1371/journal.pone.0170622 (PMC5295668; doi:10.1371/journal.pone.0170622)

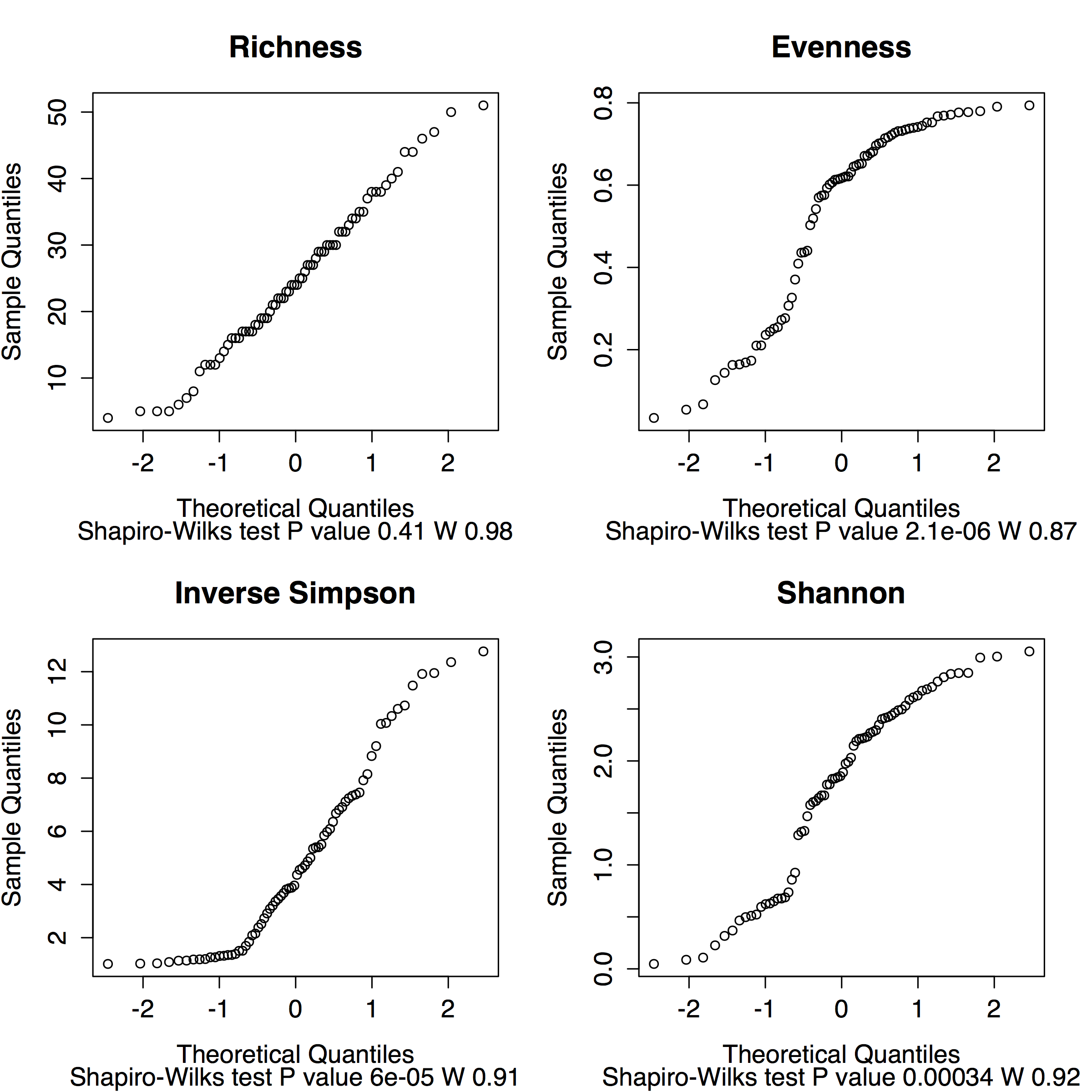

Supplement: S1 Fig — Species richness is confirmed to be normally distributed, while the other three measures, species evenness, inverse Simpson’s index and Shannon’s diversity index are non-normally distributed. (TIF) [file pone.0170622.s002.tif]

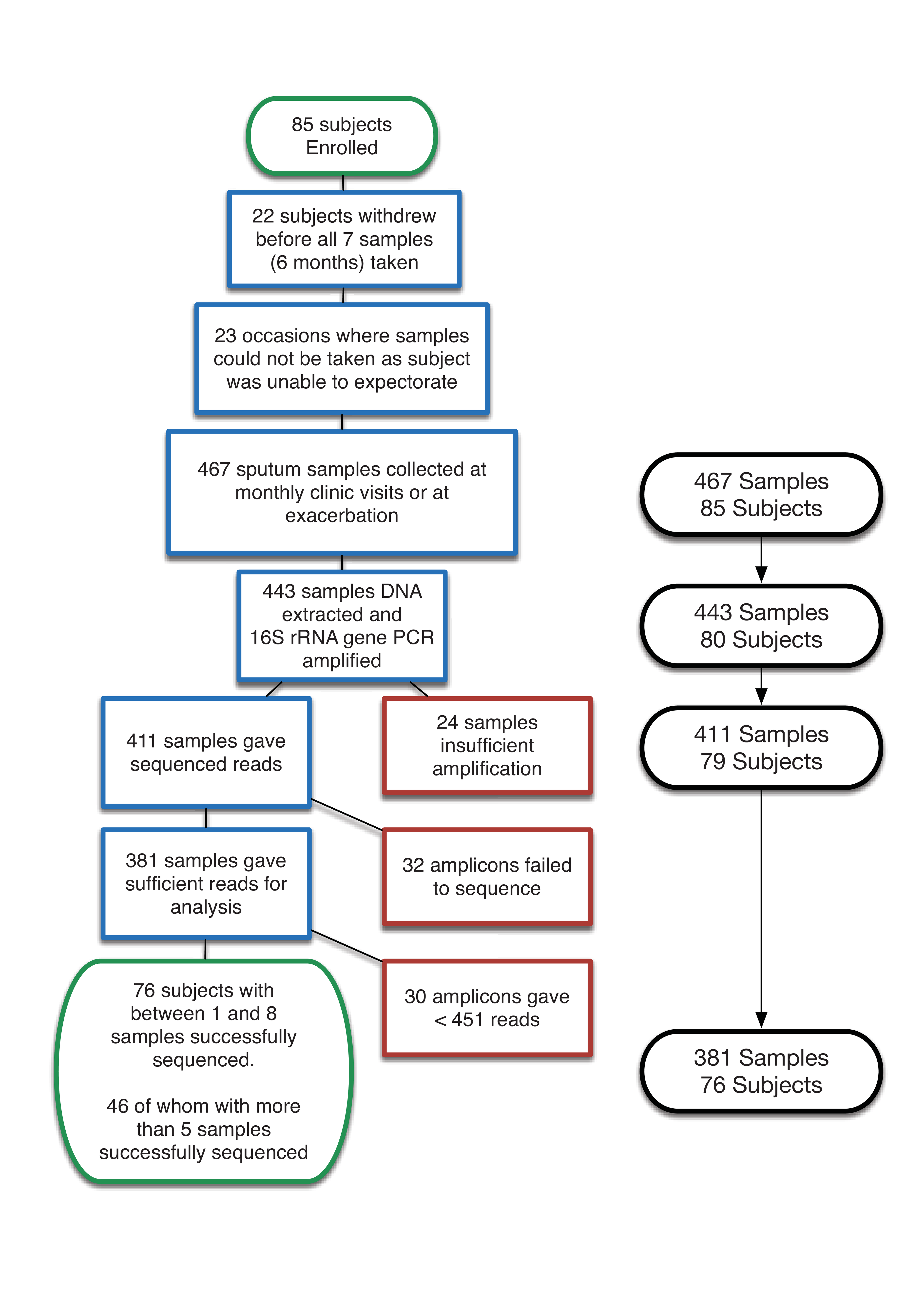

Supplement: S2 Fig — (TIF) [file pone.0170622.s003.tif]

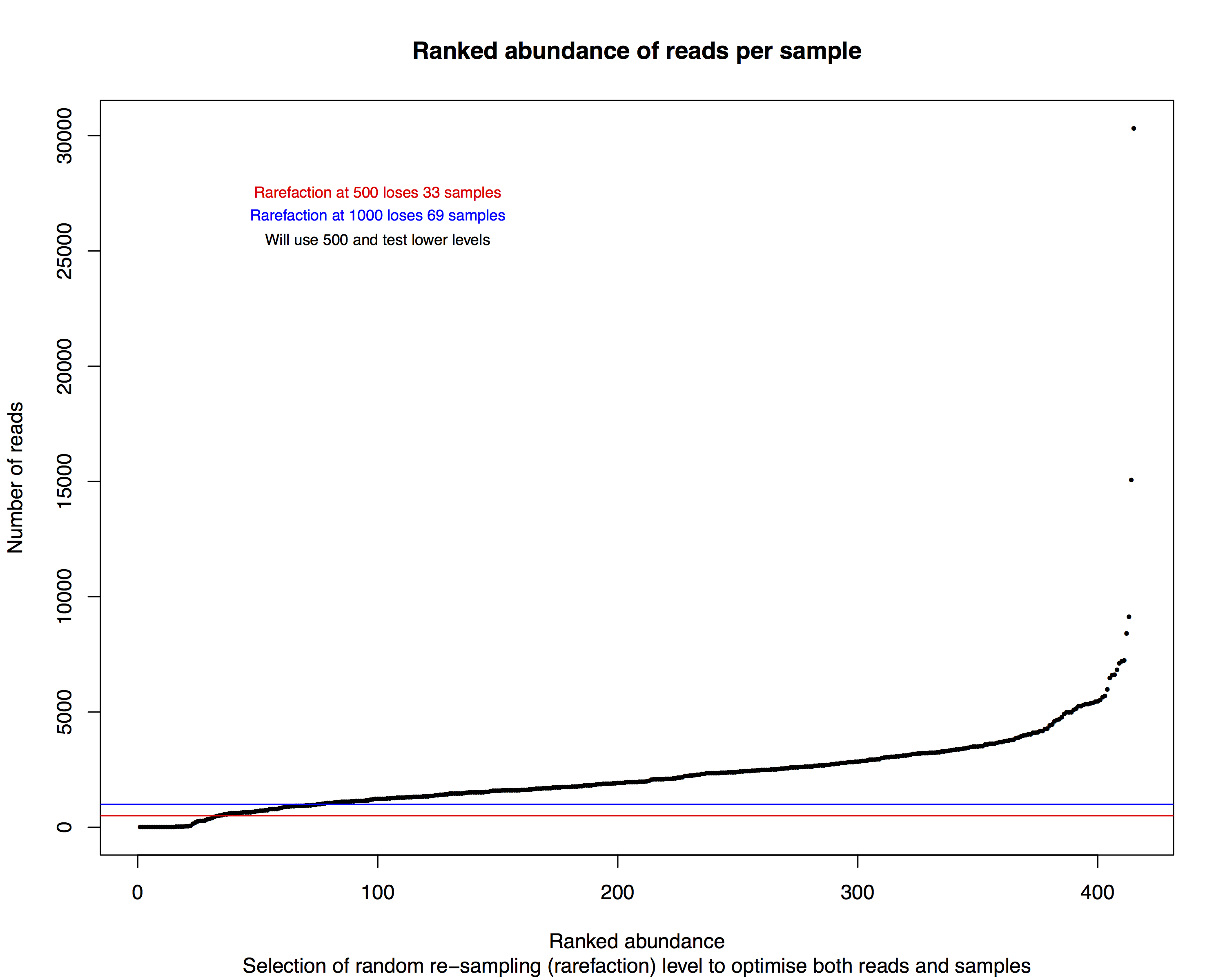

Supplement: S3 Fig — (TIF) [file pone.0170622.s004.tif]

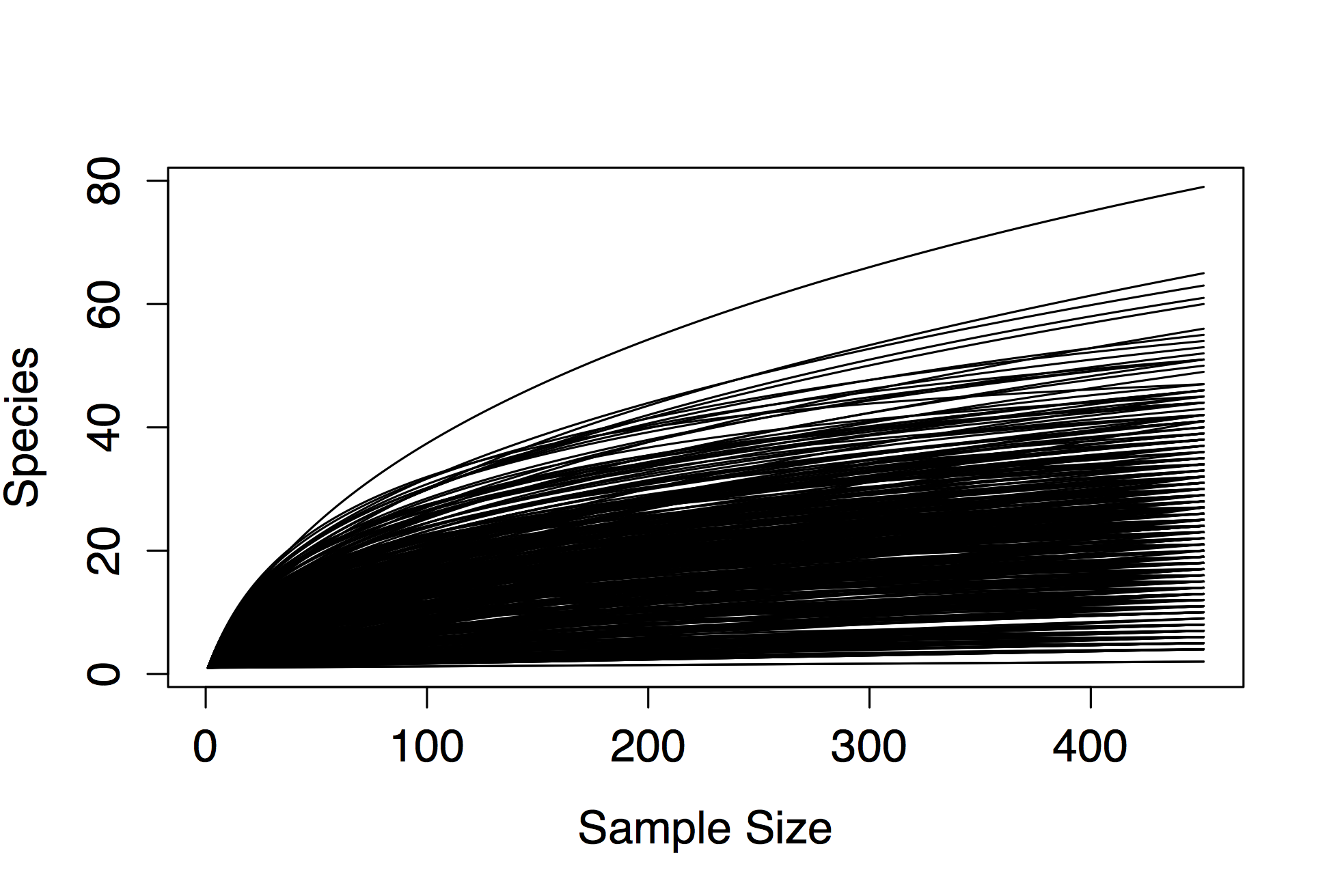

Supplement: S4 Fig — (TIF) [file pone.0170622.s005.tif]

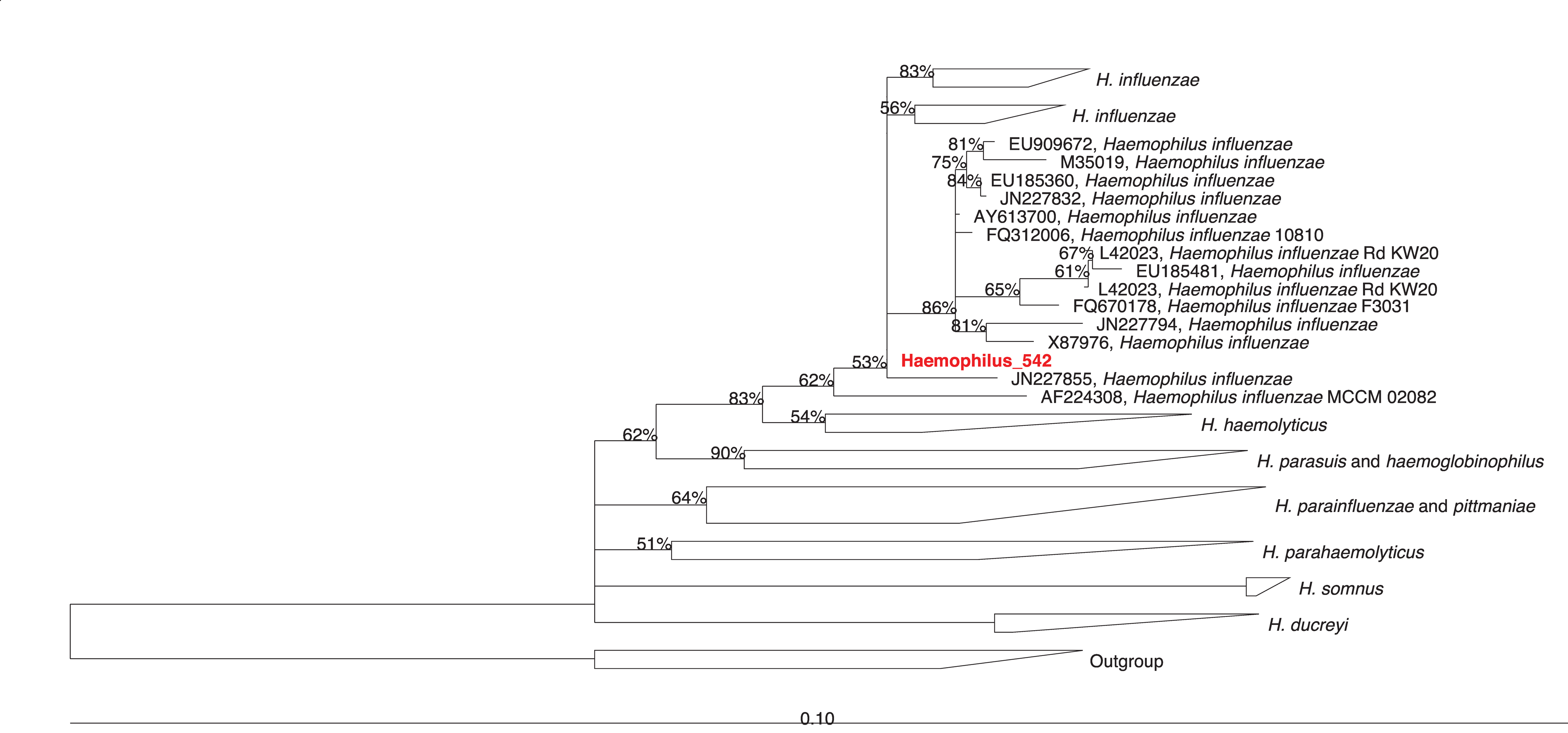

Supplement: S5 Fig — Alignment positions 509 to 909 (Escherichia coli numbering) were used to construct a neighbour joining tree of partial 16S rRNA gene sequences using ARBs (ref) neighbour function with 500 bootstraps. Bootstrap values less than 50% are collapsed to multi-furcations. The tree was rooted for display purposes with an outgroup consisting of Providencia spp., Morganella spp. and Proteus spp. The position of the Haemophilus_542 OTU is highlighted in red. (TIF) [file pone.0170622.s006.tif]

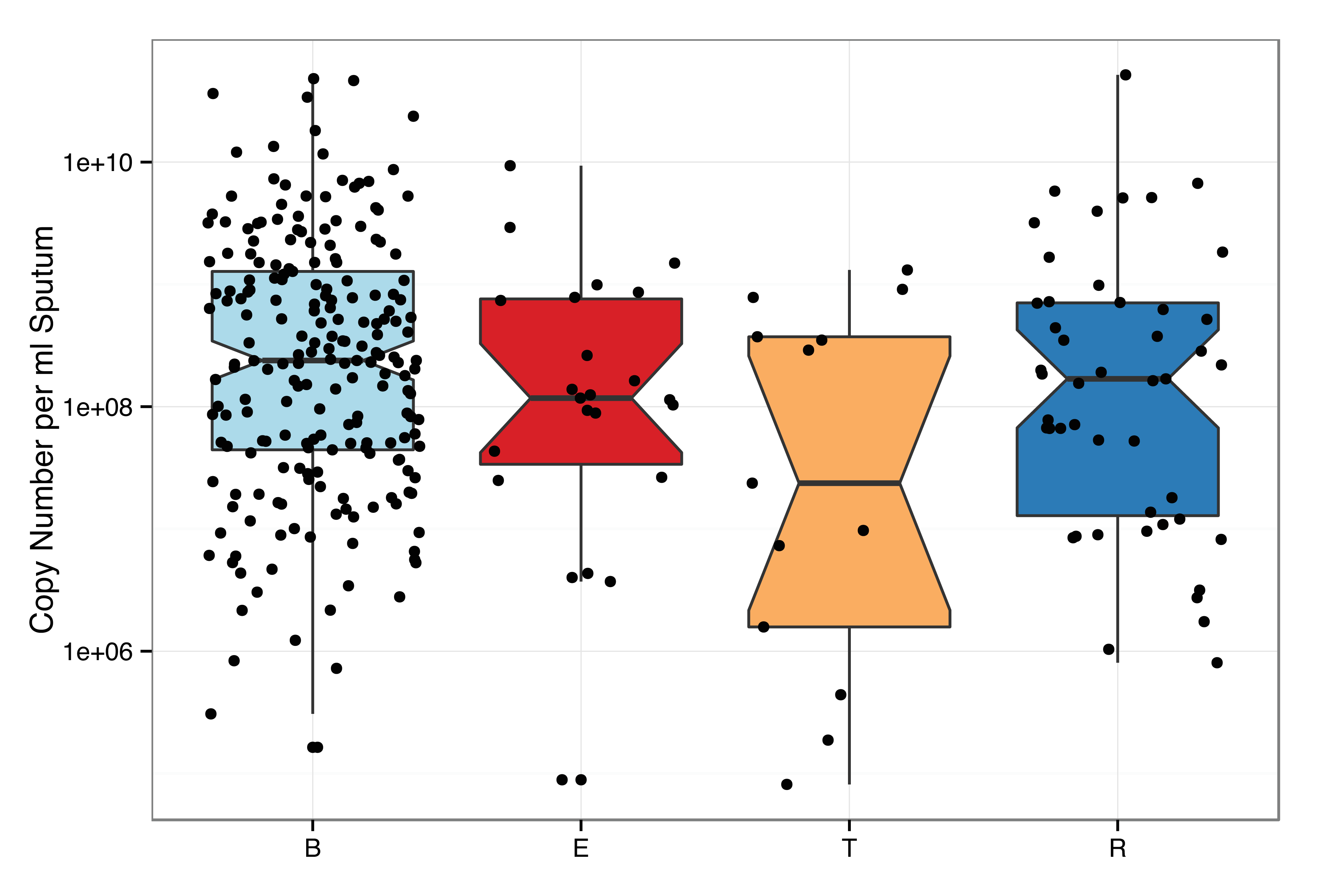

Supplement: S6 Fig — There is no significant difference in load. Samples with current treatment for exacerbation have the lowest median, though numbers of non-baseline samples are relatively low. (TIF) [file pone.0170622.s007.tif]

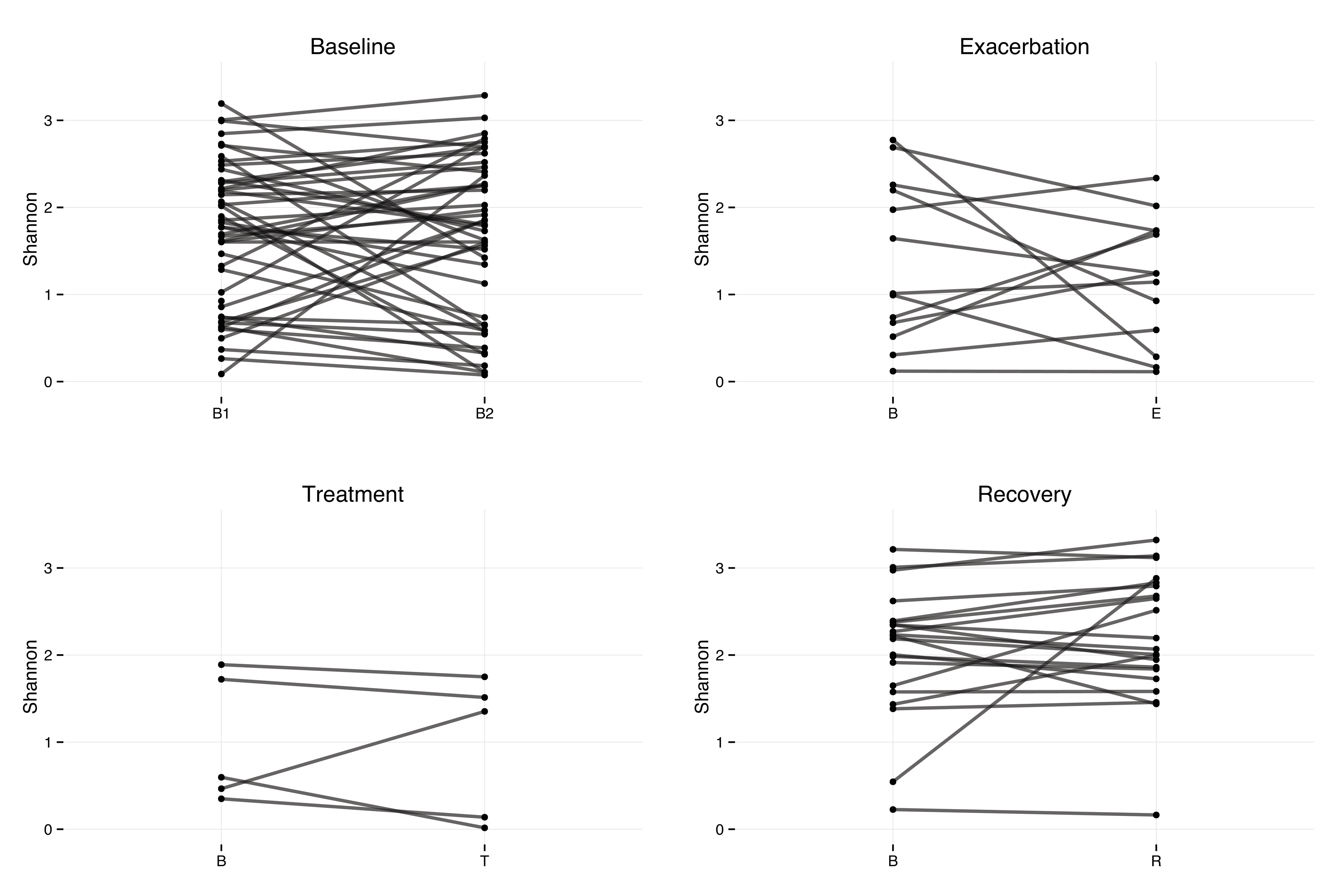

Supplement: S7 Fig — Baseline (B) samples are paired with samples taken the following month of each of the categories. It is possible that within an individual more than one pair of immediately following samples can be found, in this case, within each plot only the first from that subject was used. (TIF) [file pone.0170622.s008.tif]
