# Supplementary material for: Longitudinal assessment of sputum microbiome by sequencing of the 16S rRNA gene in non-cystic fibrosis bronchiectasis patients
Source: PLoS One. 2017 Feb 7;12(2):e0170622. doi: 10.1371/journal.pone.0170622 (PMC5295668; doi:10.1371/journal.pone.0170622)
Supplement: S8 Fig — Stacked barplots of every subject with three or more samples. As with S3 Fig each subject is represented by four plots, from top to bottom: clinical variables including antibiotic treatment, growth of microorganisms on clinical culture and B,E,T,R category; Lung function as FEV1% predicted (red), FVC % predicted (green) with 30% and 80% represented by the grey dotted line; bacterial load as measured by 16S rRNA gene qPCR in copies per ml of sputum with the detection limit of the assay indicated by the grey dotted line; stacked barplots of the OTUs present in each sample. Colour coding for top 26 OTUs is consistent between plots to allow comparison of the most common organisms; greyscale is used for the remaining OTUs, which is consistent only within each plot as there are insufficient colours for this to be possible across all plots. Rare OTUs in each plot are summed as “Other”. (PDF) [file pone.0170622.s009.pdf]

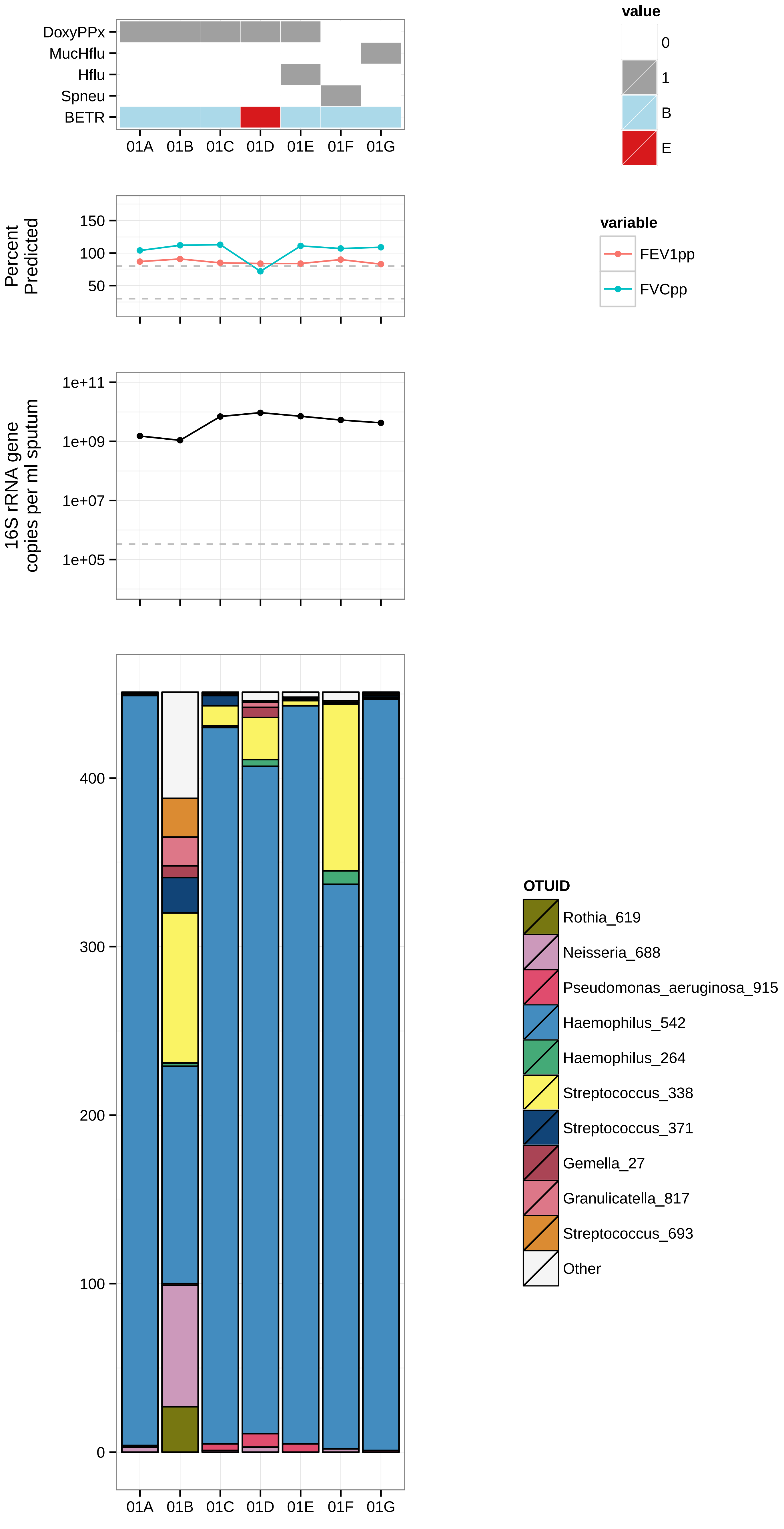

DoxyPPx
MucHflu
Hflu
Spneu
BETR
01A
01B
01C
01D
01E
01F
01G
value
0
1
B
E
Percent Predicted
150
100
50
variable
FEV1pp
FVCpp
16S rRNA gene copies per ml sputum
1e+11
1e+09
1e+07
1e+05
400
300
200
100
0
OTUID
Rothia_619
Neisseria_688
Pseudomonas_aeruginosa_915
Haemophilus_542
Haemophilus_264
Streptococcus_338
Streptococcus_371
Gemella_27
Granulicatella_817
Streptococcus_693
Other

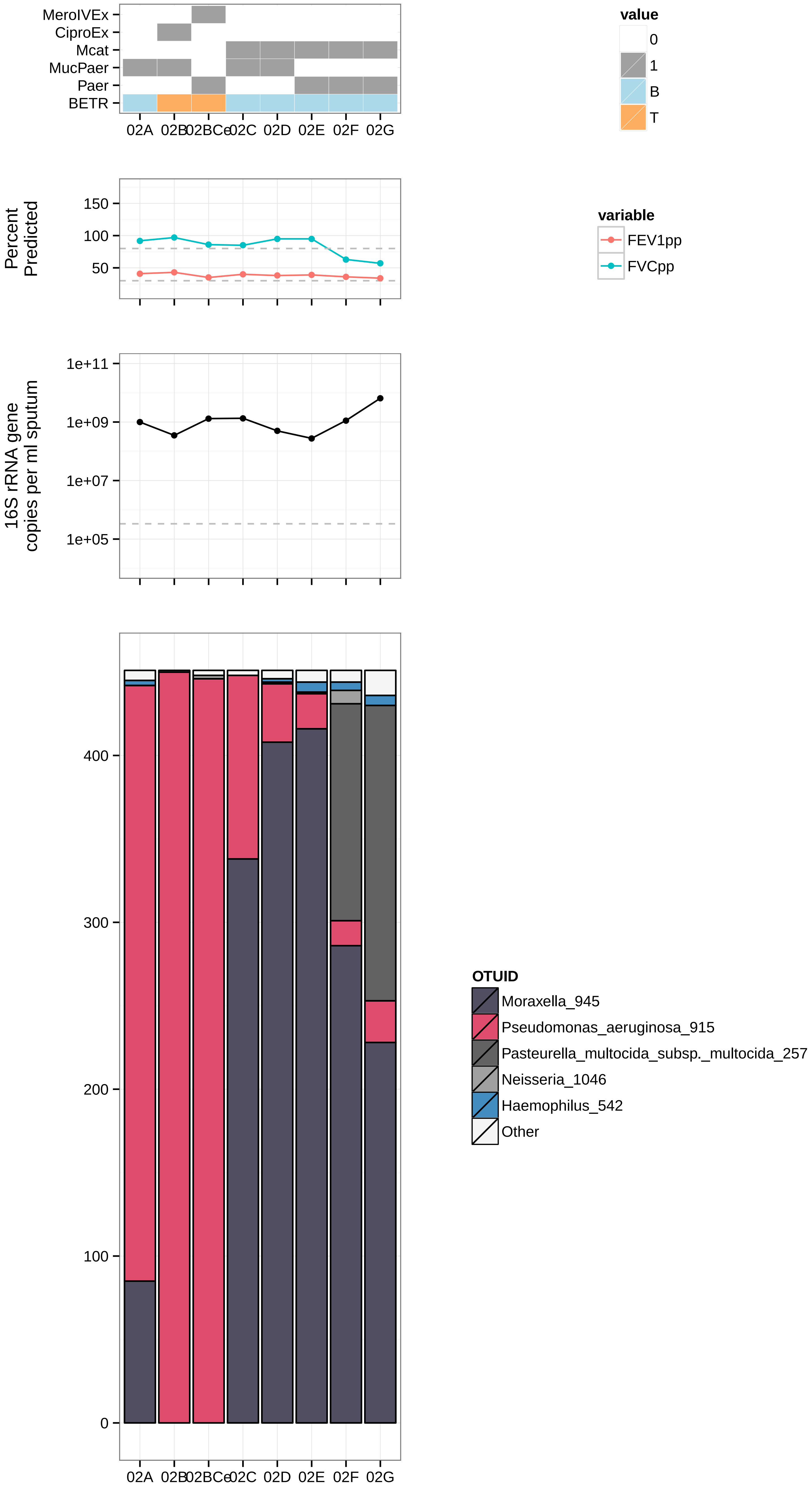

MeroIVEx
CiproEx
Mcat
MucPaer
Paer
BETR
02A 02B 02BCe 02C 02D 02E 02F 02G
value
0
1
B
T
Percent Predicted
150
100
50
variable
FEV1pp
FVCpp
16S rRNA gene copies per ml sputum
1e+11
1e+09
1e+07
1e+05
400
300
200
100
0
02A 02B 02BCe 02C 02D 02E 02F 02G
OTUID
Moraxella_945
Pseudomonas_aeruginosa_915
Pasteurella_multocida_subsp._multocida_257
Neisseria_1046
Haemophilus_542
Other

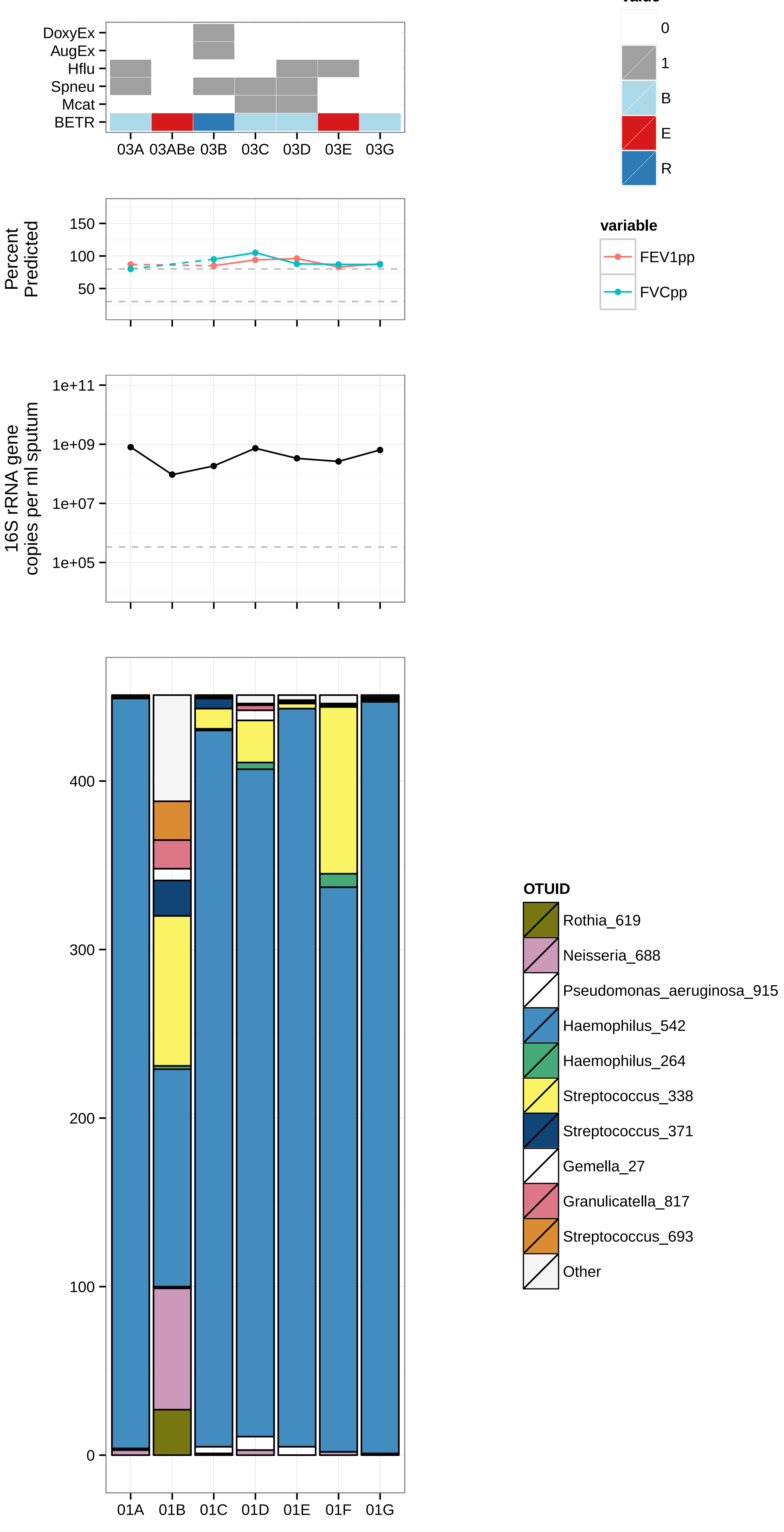

DoxyEx
AugEx
Hflu
Spneu
Mcat
BETR
03A 03ABe 03B 03C 03D 03E 03G
value
0
1
B
E
R
Percent Predicted
150
100
50
variable
FEV1pp
FVCpp
16S rRNA gene copies per ml sputum
1e+11
1e+09
1e+07
1e+05
400
300
200
100
0
01A 01B 01C 01D 01E 01F 01G
OTUID
Rothia_619
Neisseria_688
Pseudomonas_aeruginosa_915
Haemophilus_542
Haemophilus_264
Streptococcus_338
Streptococcus_371
Gemella_27
Granulicatella_817
Streptococcus_693
Other

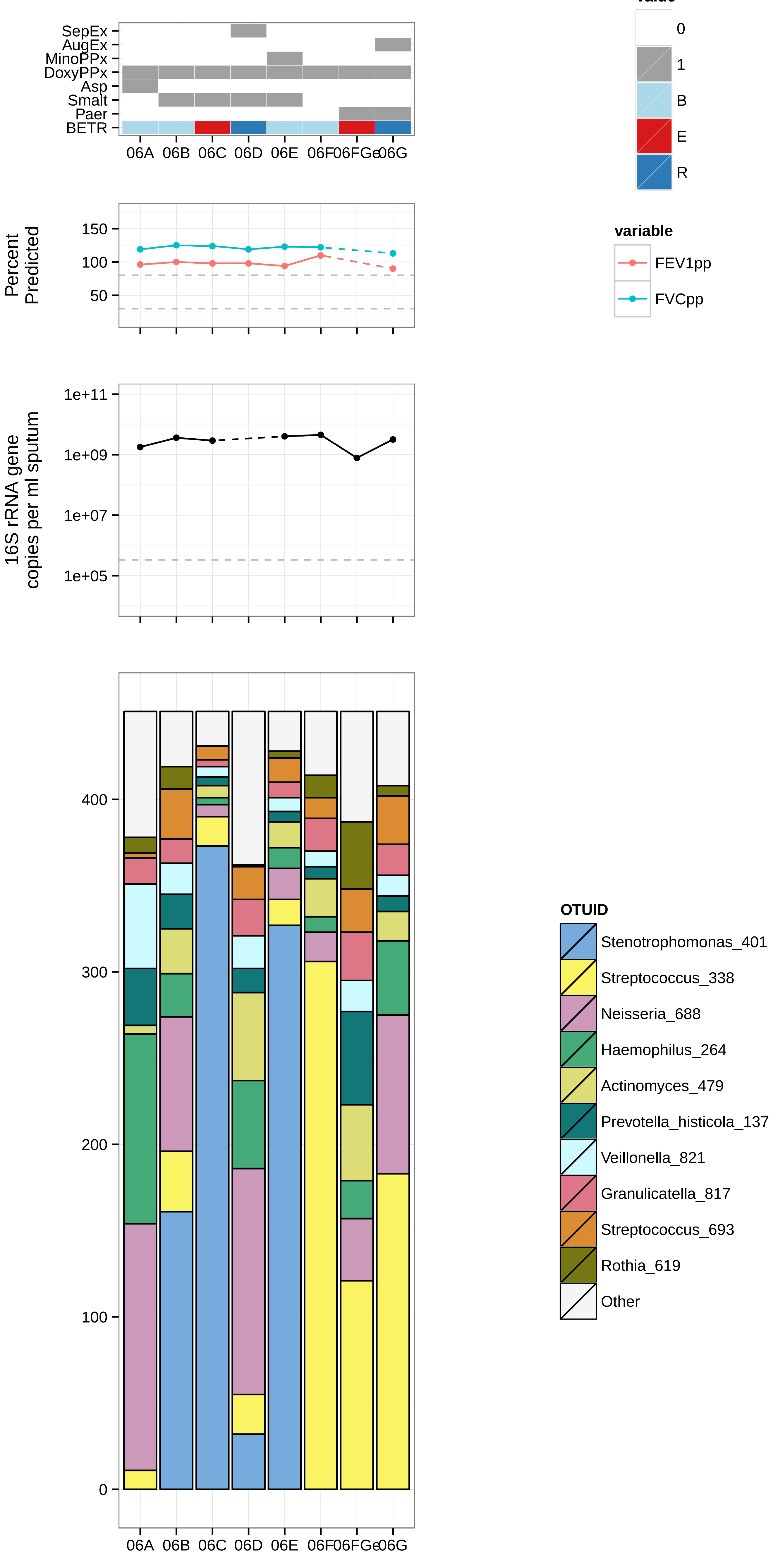

value
0
1
B
E
R
SepEx
AugEx
MinoPPx
DoxyPPx
Asp
Smalt
Paer
BETR
06A 06B 06C 06D 06E 06F 06FGe 06G
Percent Predicted
150
100
50
variable
FEV1pp
FVCpp
16S rRNA gene copies per ml sputum
1e+11
1e+09
1e+07
1e+05
400
300
200
100
0
06A 06B 06C 06D 06E 06F 06FGe 06G
OTUID
Stenotrophomonas_401
Streptococcus_338
Neisseria_688
Haemophilus_264
Actinomyces_479
Prevotella_histicola_137
Veillonella_821
Granulicatella_817
Streptococcus_693
Rothia_619
Other

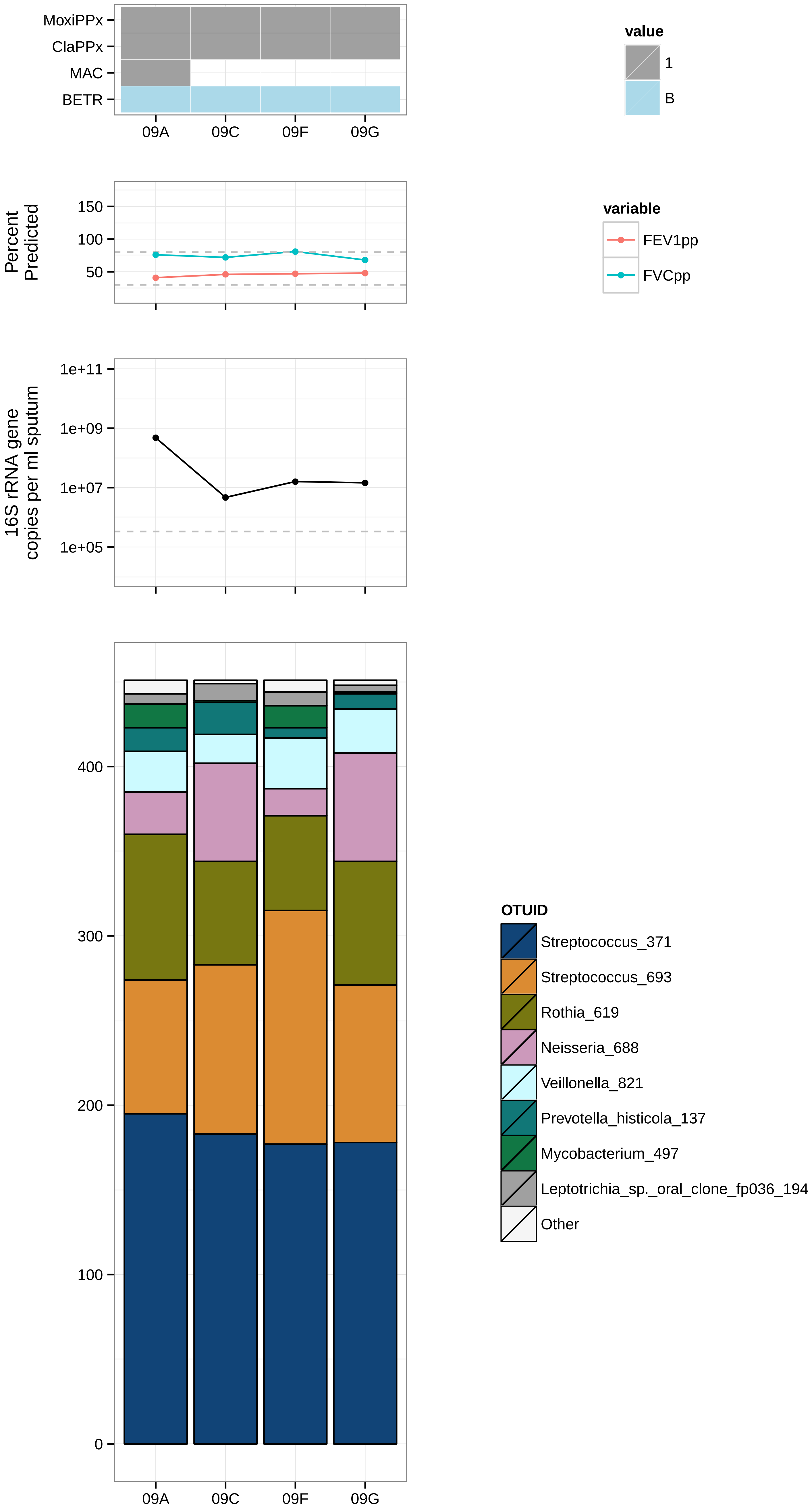
MoxiPPx
ClaPPx
MAC
BETR
09A
09C
09F
09G
value
1
B
Percent Predicted
150
100
50
variable
FEV1pp
FVCpp
16S rRNA gene copies per ml sputum
1e+11
1e+09
1e+07
1e+05
400
300
200
100
0
OTUID
Streptococcus_371
Streptococcus_693
Rothia_619
Neisseria_688
Veillonella_821
Prevotella_histicola_137
Mycobacterium_497
Leptotrichia_sp._oral_clone_fp036_194
Other

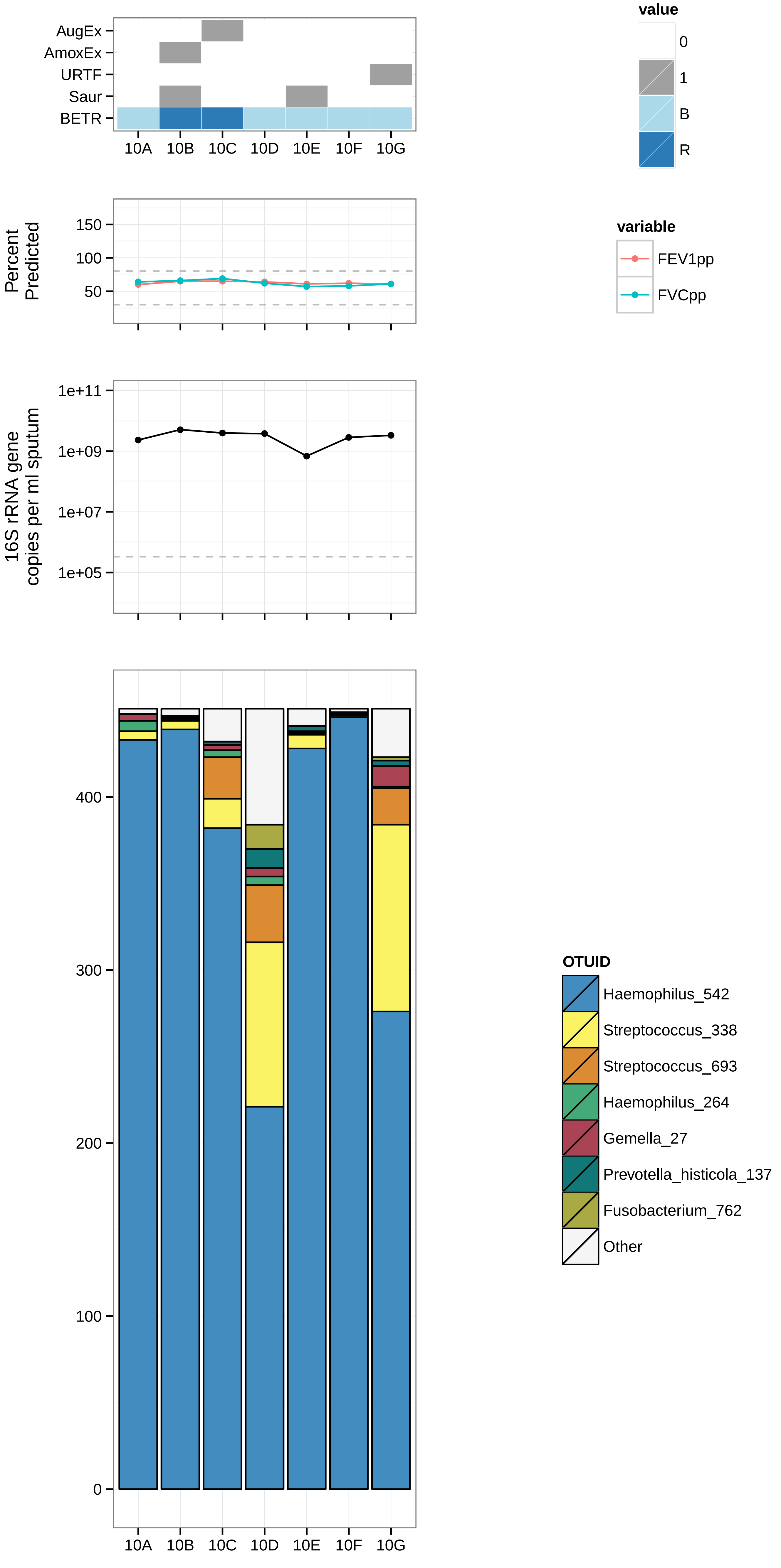

value
0
1
B
R
AugEx
AmoxEx
URTF
Saur
BETR
10A
10B
10C
10D
10E
10F
10G
Percent Predicted
150
100
50
variable
FEV1pp
FVCpp
16S rRNA gene copies per ml sputum
1e+11
1e+09
1e+07
1e+05
400
300
200
100
0
OTUID
Haemophilus_542
Streptococcus_338
Streptococcus_693
Haemophilus_264
Gemella_27
Prevotella_histicola_137
Fusobacterium_762
Other

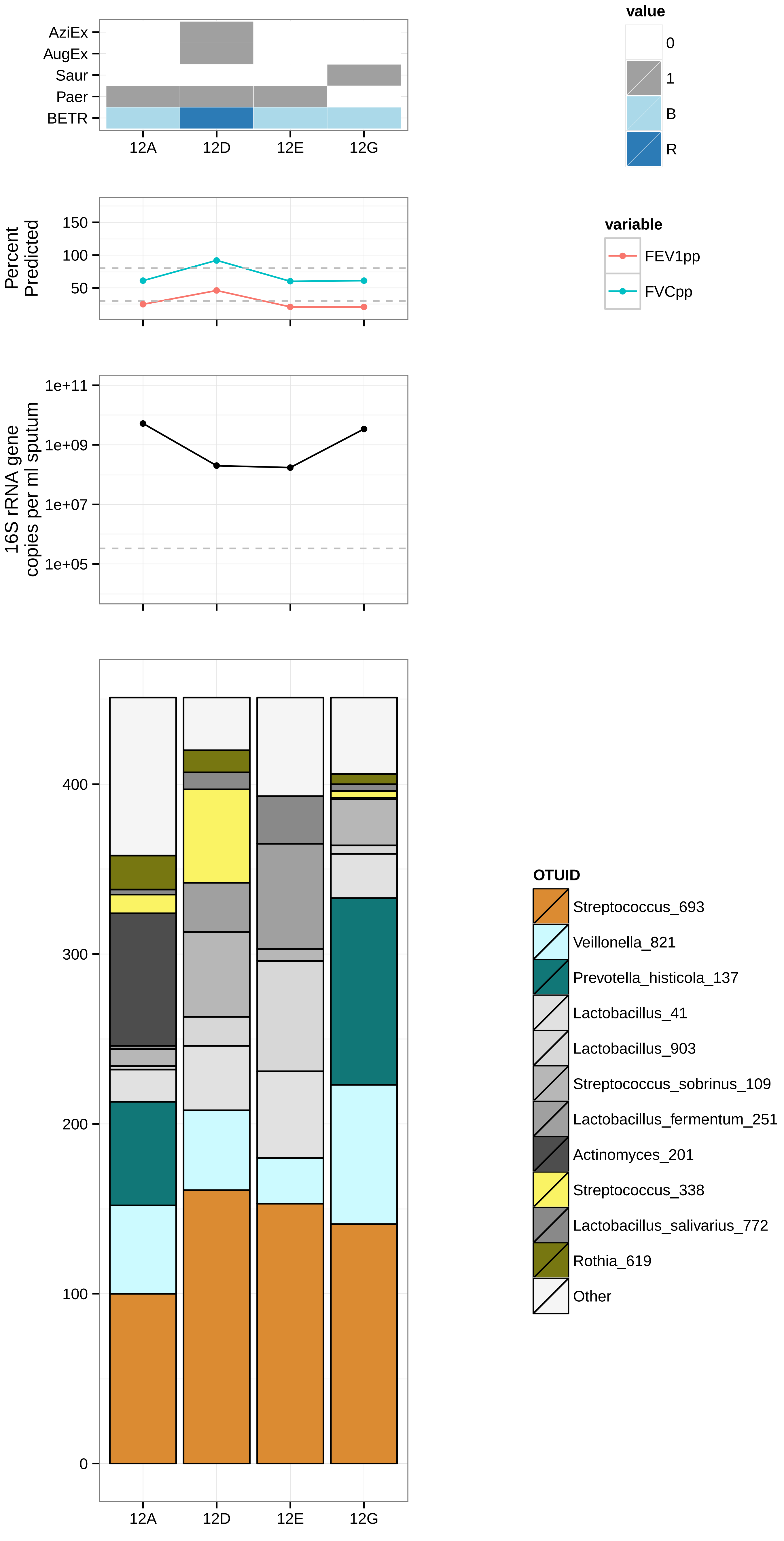

AziEx
AugEx
Saur
Paer
BETR
12A
12D
12E
12G
value
0
1
B
R
Percent Predicted
150
100
50
variable
FEV1pp
FVCpp
16S rRNA gene copies per ml sputum
1e+11
1e+09
1e+07
1e+05
400
300
200
100
0
OTUID
Streptococcus_693
Veillonella_821
Prevotella_histicola_137
Lactobacillus_41
Lactobacillus_903
Streptococcus_sobrinus_109
Lactobacillus_fermentum_251
Actinomyces_201
Streptococcus_338
Lactobacillus_salivarius_772
Rothia_619
Other

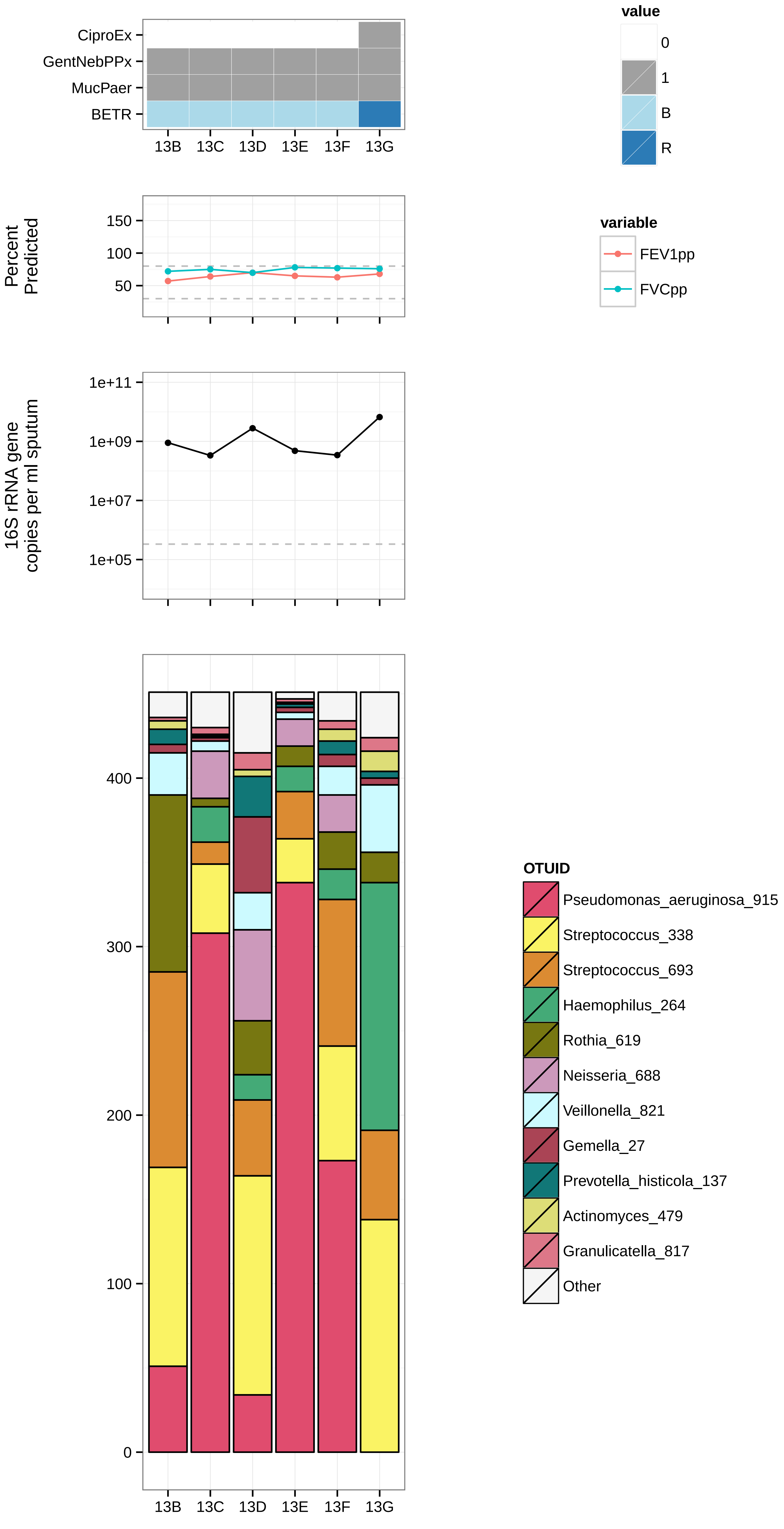

CiproEx
GentNebPPx
MucPaer
BETR
13B
13C
13D
13E
13F
13G
value
0
1
B
R
Percent Predicted
150
100
50
variable
FEV1pp
FVCpp
16S rRNA gene copies per ml sputum
1e+11
1e+09
1e+07
1e+05
400
300
200
100
0
OTUID
Pseudomonas_aeruginosa_915
Streptococcus_338
Streptococcus_693
Haemophilus_264
Rothia_619
Neisseria_688
Veillonella_821
Gemella_27
Prevotella_histicola_137
Actinomyces_479
Granulicatella_817
Other

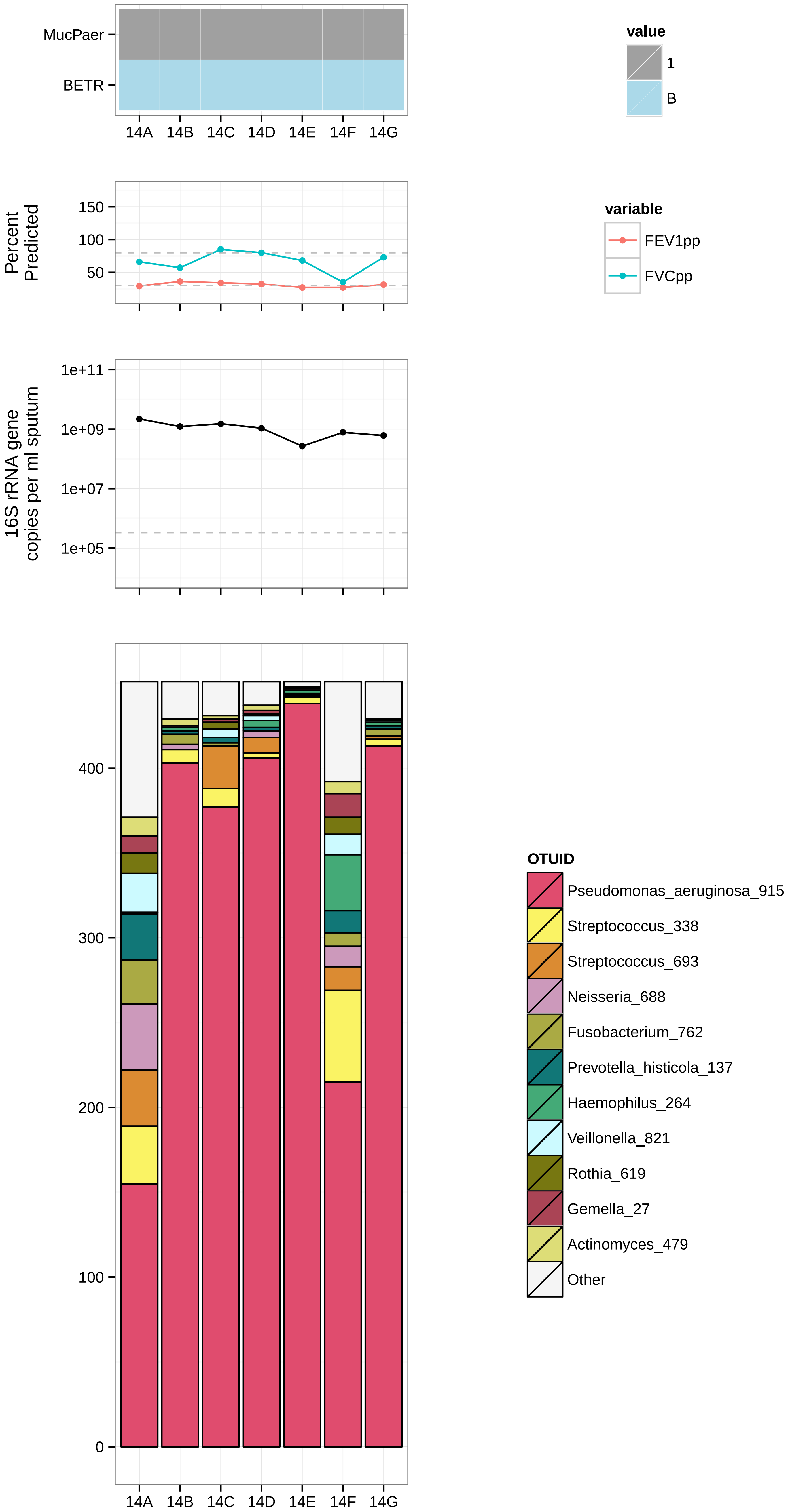
MucPaer
BETR
14A 14B 14C 14D 14E 14F 14G
value
1
B
Percent Predicted
150
100
50
variable
FEV1pp
FVCpp
16S rRNA gene copies per ml sputum
1e+11
1e+09
1e+07
1e+05
400
300
200
100
0
OTUID
Pseudomonas_aeruginosa_915
Streptococcus_338
Streptococcus_693
Neisseria_688
Fusobacterium_762
Prevotella_histicola_137
Haemophilus_264
Veillonella_821
Rothia_619
Gemella_27
Actinomyces_479
Other

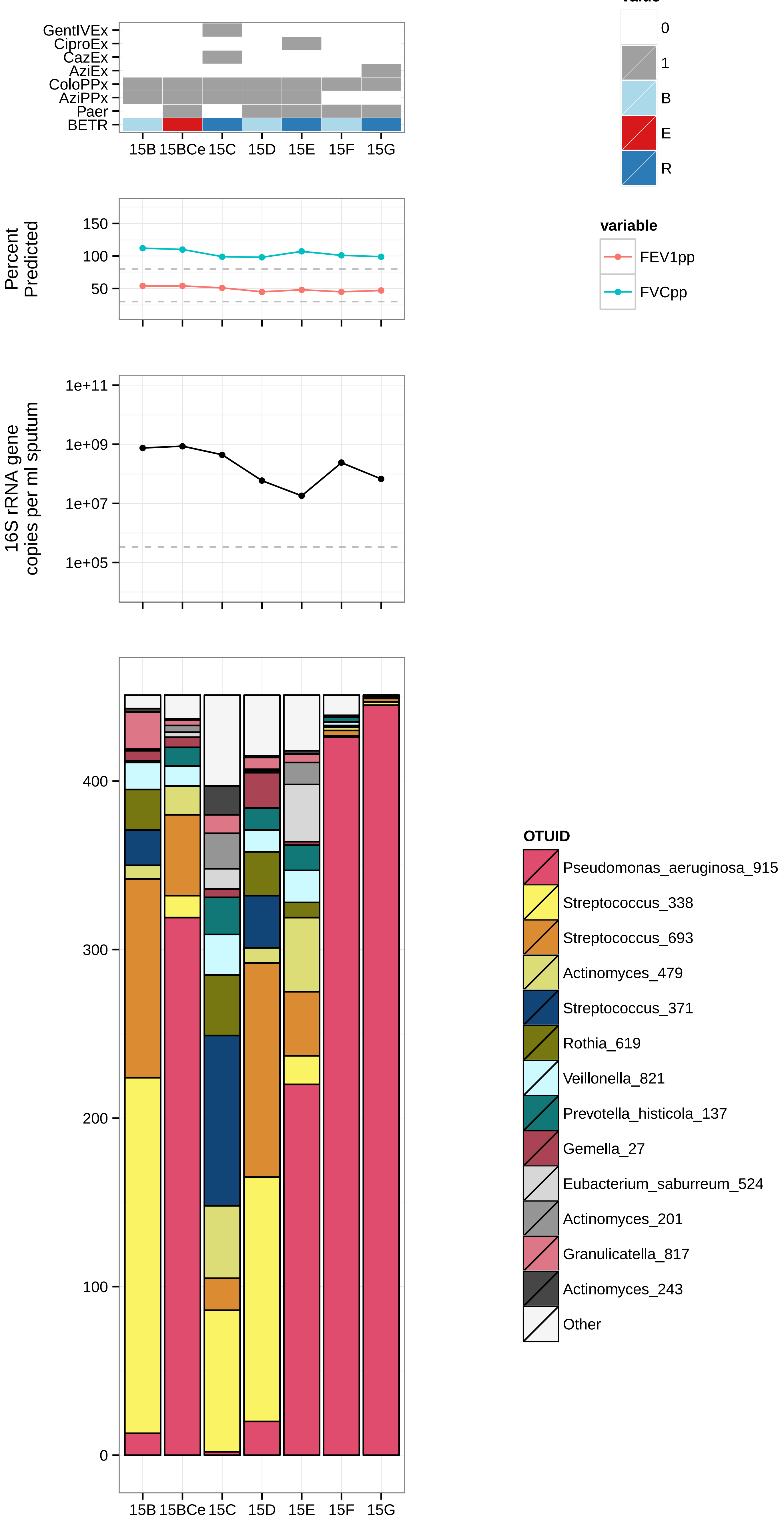

GentIVEx
CiproEx
CazEx
AziEx
ColoPPx
AziPPx
Paer
BETR
15B
15BCe
15C
15D
15E
15F
15G
0
1
B
E
R
Percent Predicted
150
100
50
variable
FEV1pp
FVCpp
16S rRNA gene copies per ml sputum
1e+11
1e+09
1e+07
1e+05
400
300
200
100
0
OTUID
Pseudomonas_aeruginosa_915
Streptococcus_338
Streptococcus_693
Actinomyces_479
Streptococcus_371
Rothia_619
Veillonella_821
Prevotella_histicola_137
Gemella_27
Eubacterium_saburreum_524
Actinomyces_201
Granulicatella_817
Actinomyces_243
Other

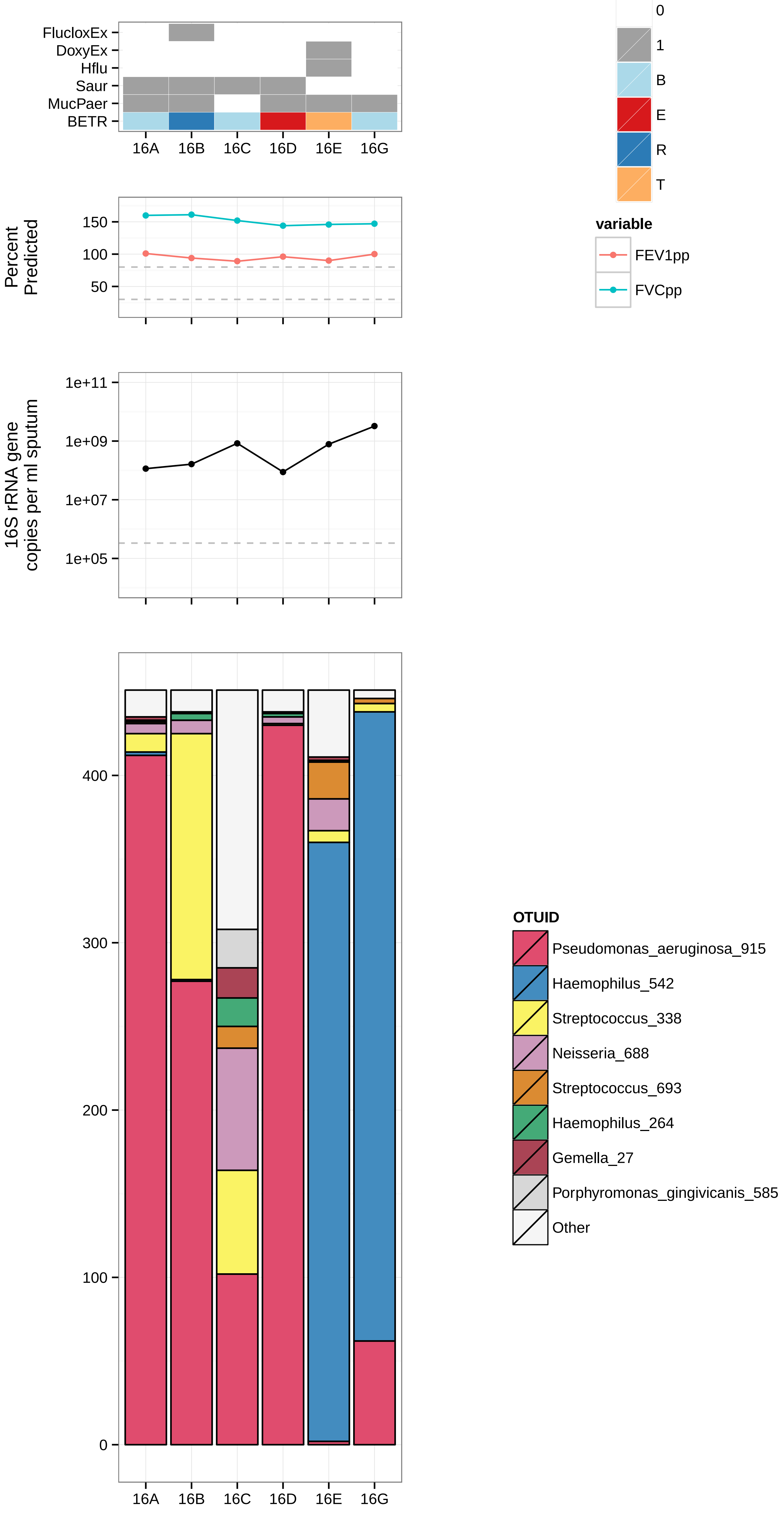

FlucloxEx
DoxyEx
Hflu
Saur
MucPaer
BETR
16A
16B
16C
16D
16E
16G
0
1
B
E
R
T
Percent Predicted
150
100
50
variable
FEV1pp
FVCpp
16S rRNA gene copies per ml sputum
1e+11
1e+09
1e+07
1e+05
400
300
200
100
0
OTUID
Pseudomonas_aeruginosa_915
Haemophilus_542
Streptococcus_338
Neisseria_688
Streptococcus_693
Haemophilus_264
Gemella_27
Porphyromonas_gingivicanis_585
Other

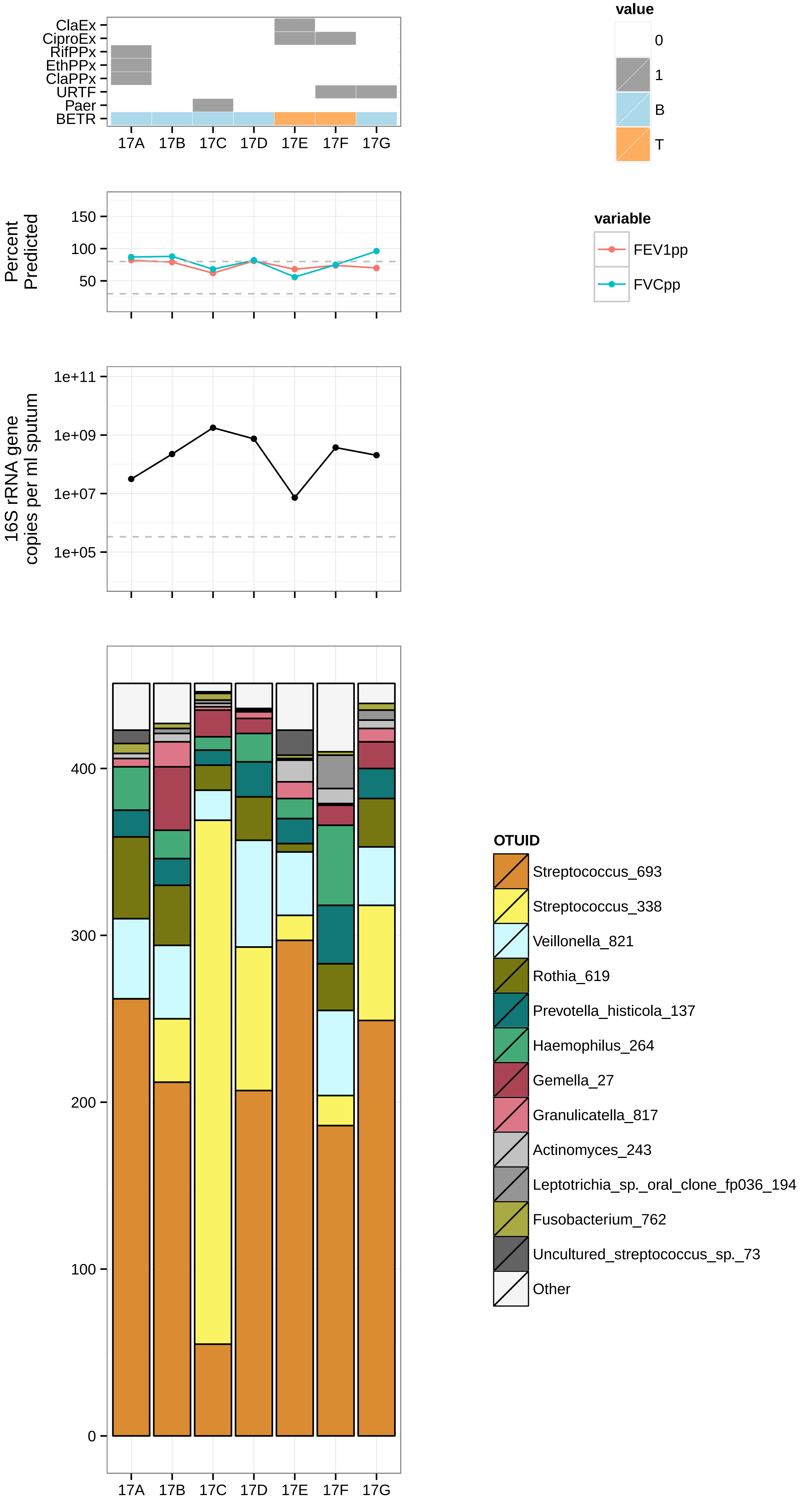
ClaEx
CiproEx
RifPPx
EthPPx
ClaPPx
URTF
Paer
BETR
17A
17B
17C
17D
17E
17F
17G
value
0
1
B
T
Percent Predicted
150
100
50
variable
FEV1pp
FVCpp
16S rRNA gene copies per ml sputum
1e+11
1e+09
1e+07
1e+05
400
300
200
100
0
OTUID
Streptococcus_693
Streptococcus_338
Veillonella_821
Rothia_619
Prevotella_histicola_137
Haemophilus_264
Gemella_27
Granulicatella_817
Actinomyces_243
Leptotrichia_sp._oral_clone_fp036_194
Fusobacterium_762
Uncultured_streptococcus_sp._73
Other

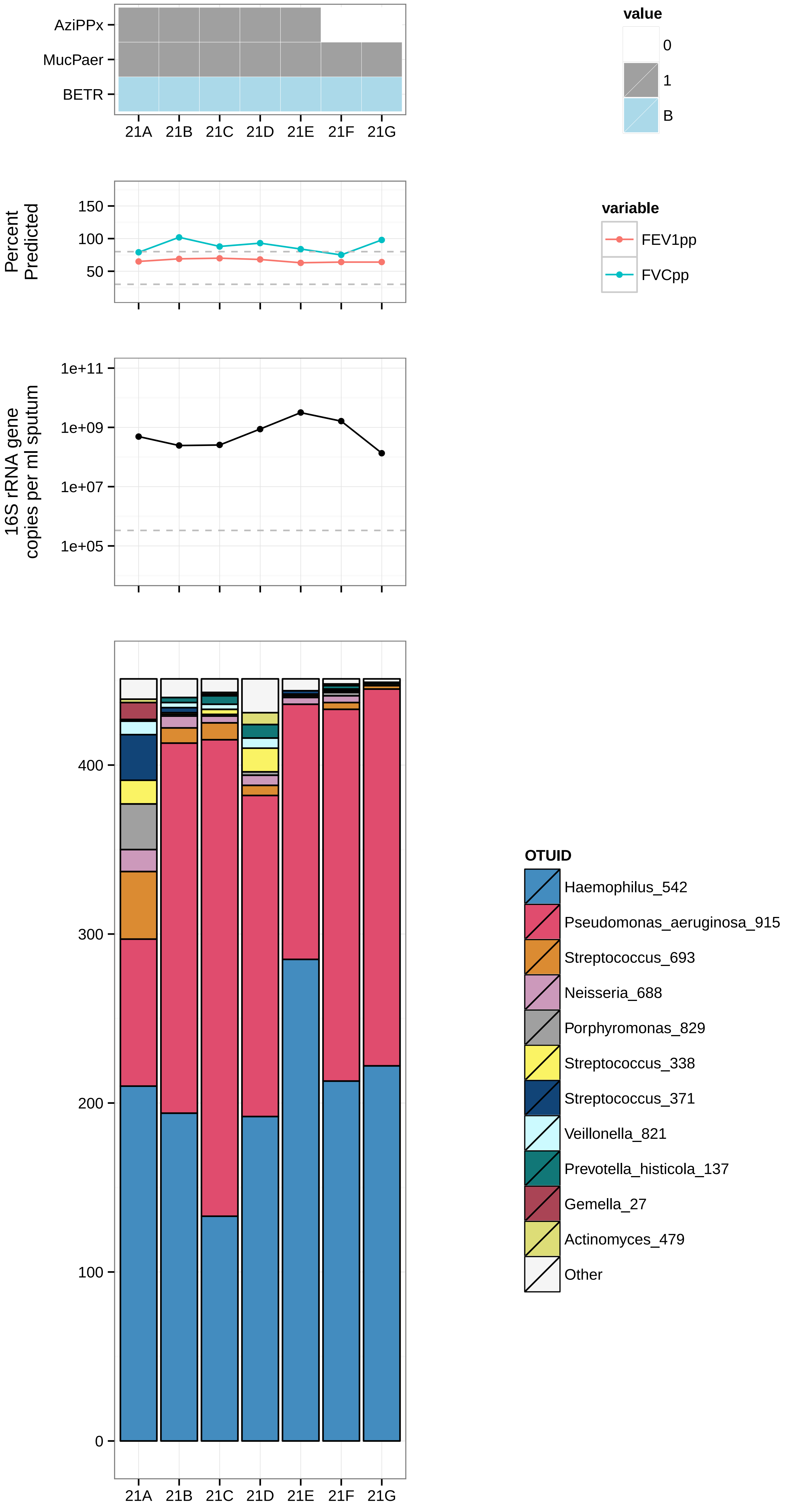

AziPPx
MucPaer
BETR
21A
21B
21C
21D
21E
21F
21G
value
0
1
B
Percent Predicted
150
100
50
variable
FEV1pp
FVCpp
16S rRNA gene copies per ml sputum
1e+11
1e+09
1e+07
1e+05
400
300
200
100
0
OTUID
Haemophilus_542
Pseudomonas_aeruginosa_915
Streptococcus_693
Neisseria_688
Porphyromonas_829
Streptococcus_338
Streptococcus_371
Veillonella_821
Prevotella_histicola_137
Gemella_27
Actinomyces_479
Other

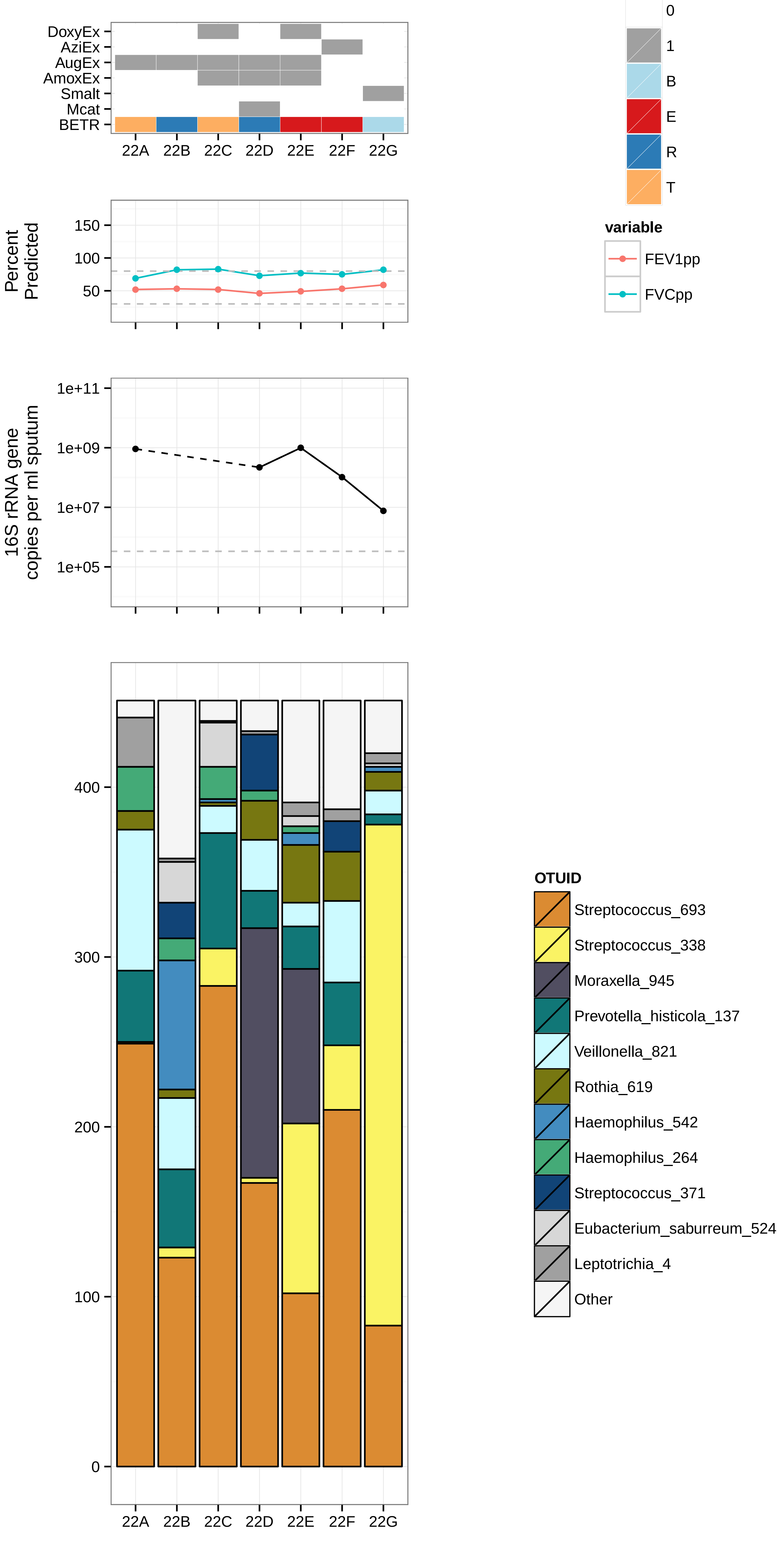

DoxyEx
AziEx
AugEx
AmoxEx
Smalt
Mcat
BETR
22A 22B 22C 22D 22E 22F 22G
0
1
B
E
R
T
Percent Predicted
150
100
50
variable
FEV1pp
FVCpp
16S rRNA gene copies per ml sputum
1e+11
1e+09
1e+07
1e+05
400
300
200
100
0
OTUID
Streptococcus_693
Streptococcus_338
Moraxella_945
Prevotella_histicola_137
Veillonella_821
Rothia_619
Haemophilus_542
Haemophilus_264
Streptococcus_371
Eubacterium_saburreum_524
Leptotrichia_4
Other

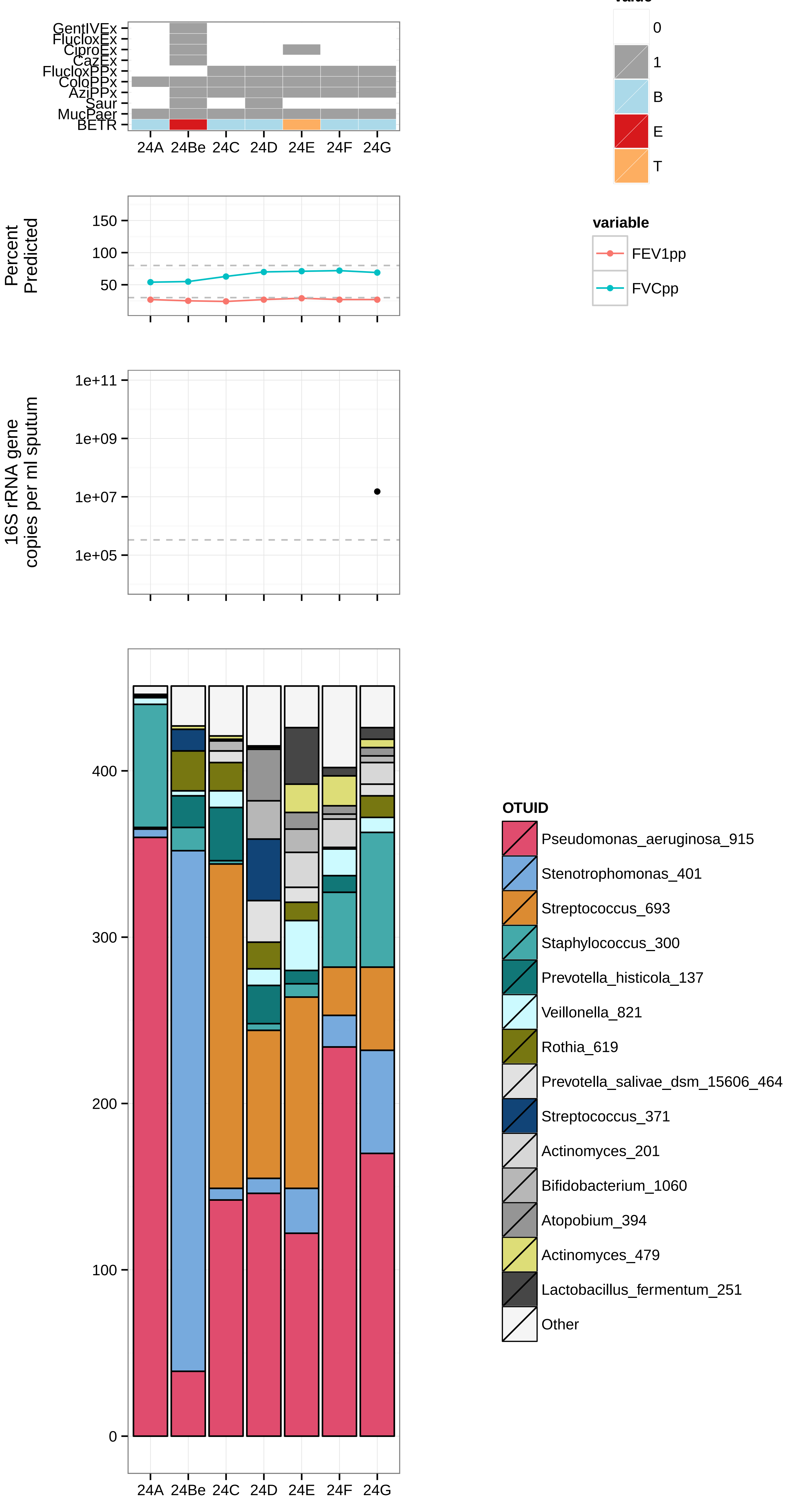
GentIVEx
FlucloxEx
CiproEx
CazEx
FlucloxPPx
ColoPPx
AziPPx
Saur
MucPaer
BETR
24A 24Be 24C 24D 24E 24F 24G
value
0
1
B
E
T
Percent Predicted
150
100
50
variable
FEV1pp
FVCpp
16S rRNA gene copies per ml sputum
1e+11
1e+09
1e+07
1e+05
400
300
200
100
0
OTUID
Pseudomonas_aeruginosa_915
Stenotrophomonas_401
Streptococcus_693
Staphylococcus_300
Prevotella_histicola_137
Veillonella_821
Rothia_619
Prevotella_salivae_dsm_15606_464
Streptococcus_371
Actinomyces_201
Bifidobacterium_1060
Atopobium_394
Actinomyces_479
Lactobacillus_fermentum_251
Other

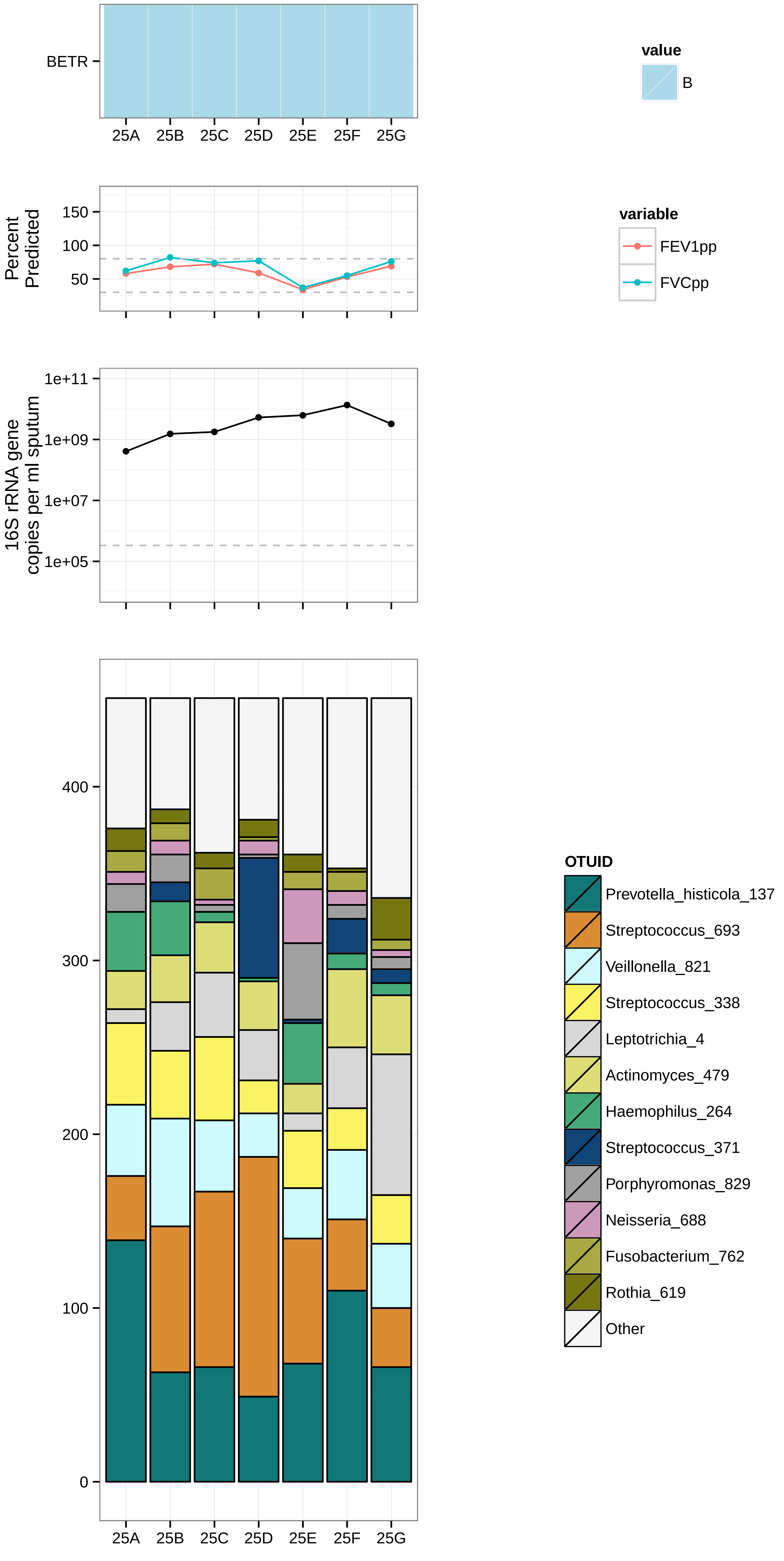
BETR
value
B
25A
25B
25C
25D
25E
25F
25G
Percent Predicted
150
100
50
variable
FEV1pp
FVCpp
16S rRNA gene copies per ml sputum
1e+11
1e+09
1e+07
1e+05
400
300
200
100
0
OTUID
Prevotella_histicola_137
Streptococcus_693
Veillonella_821
Streptococcus_338
Leptotrichia_4
Actinomyces_479
Haemophilus_264
Streptococcus_371
Porphyromonas_829
Neisseria_688
Fusobacterium_762
Rothia_619
Other

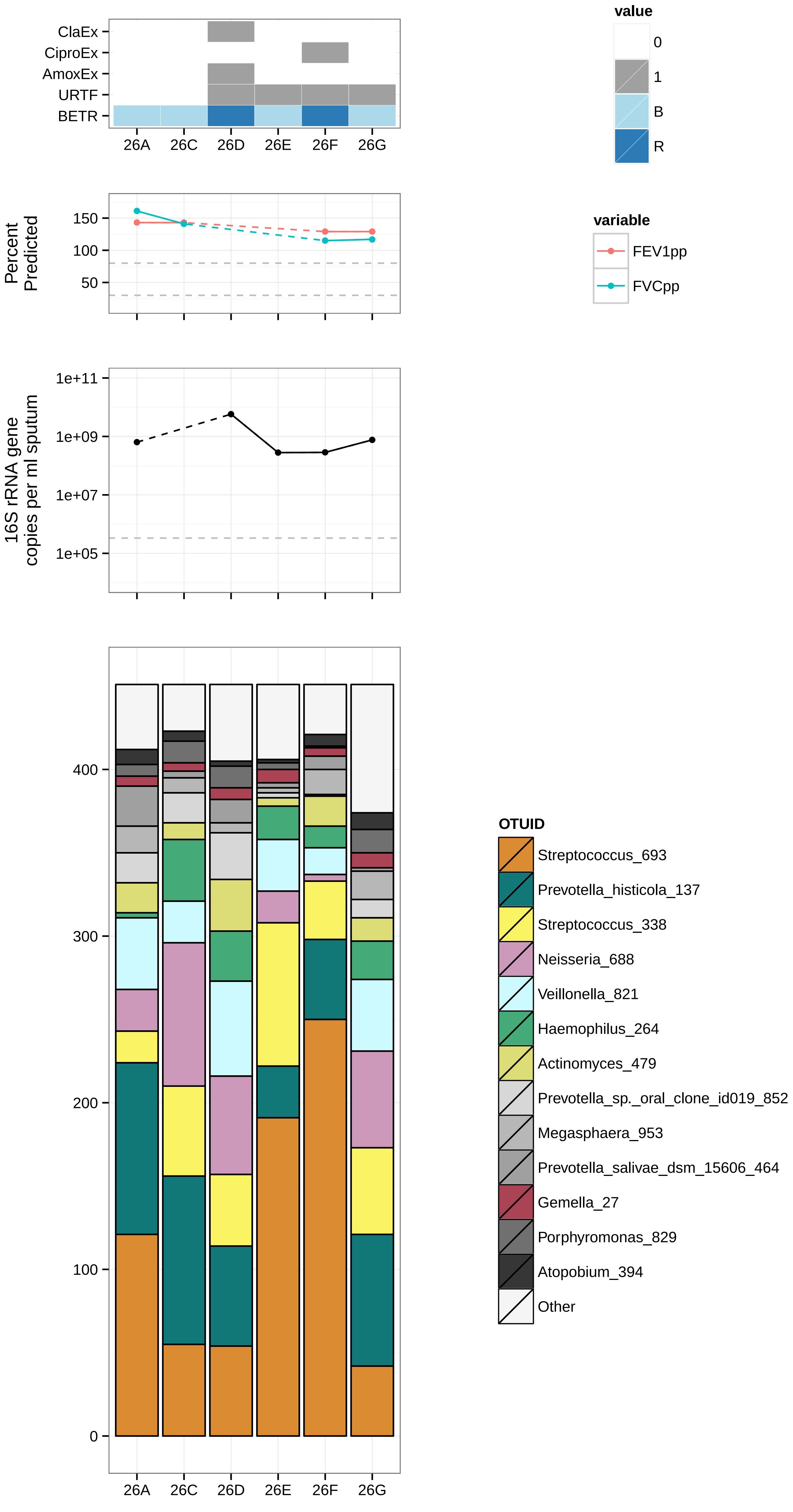

ClaEx
CiproEx
AmoxEx
URTF
BETR
26A
26C
26D
26E
26F
26G
value
0
1
B
R
Percent Predicted
150
100
50
variable
FEV1pp
FVCpp
16S rRNA gene copies per ml sputum
1e+11
1e+09
1e+07
1e+05
400
300
200
100
0
OTUID
Streptococcus_693
Prevotella_histicola_137
Streptococcus_338
Neisseria_688
Veillonella_821
Haemophilus_264
Actinomyces_479
Prevotella_sp._oral_clone_id019_852
Megasphaera_953
Prevotella_salivae_dsm_15606_464
Gemella_27
Porphyromonas_829
Atopobium_394
Other

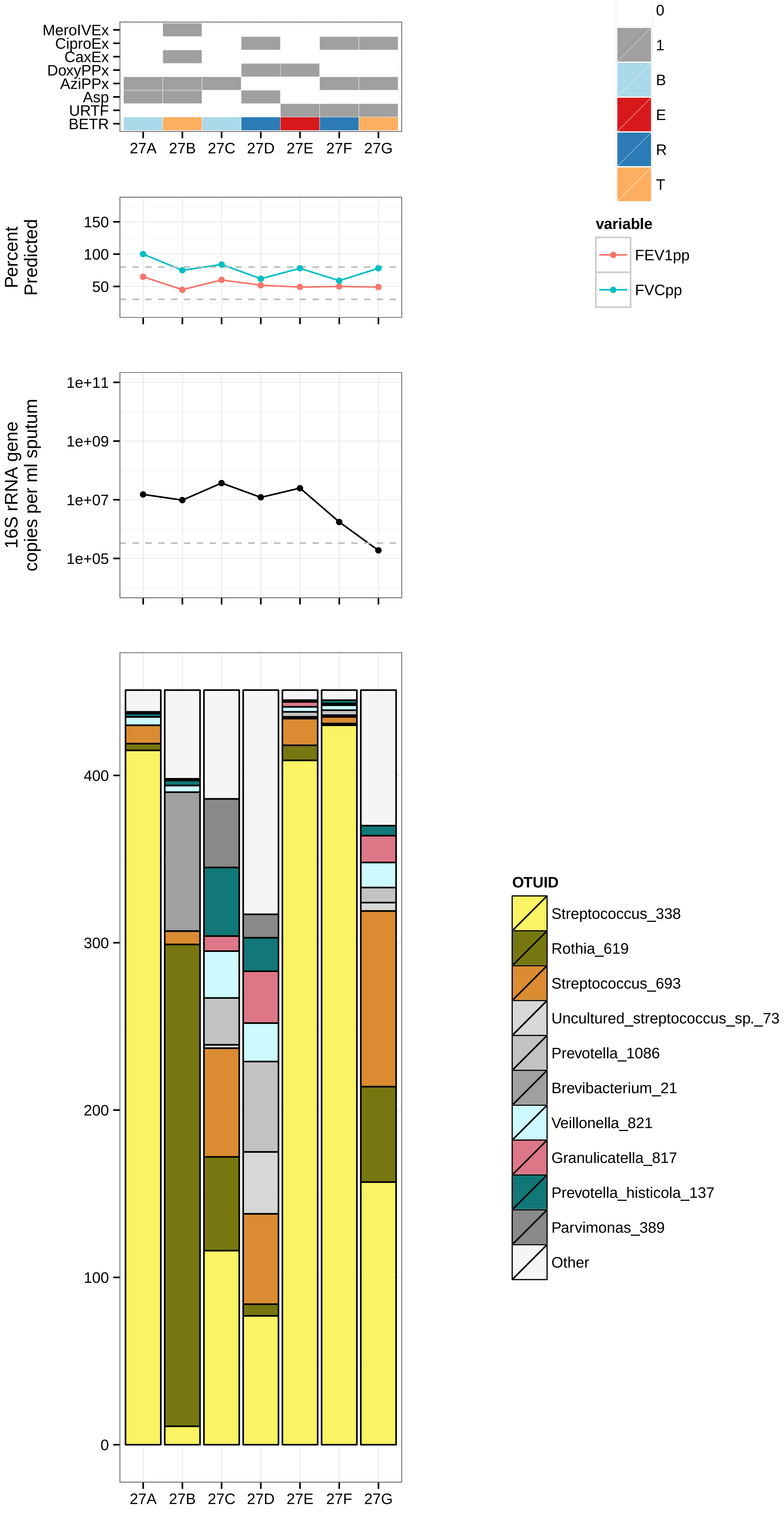
MeroIVEx
CiproEx
CaxEx
DoxyPPx
AziPPx
Asp
URTF
BETR
27A 27B 27C 27D 27E 27F 27G
0
1
B
E
R
T
Percent Predicted
150
100
50
variable
FEV1pp
FVCpp
16S rRNA gene copies per ml sputum
1e+11
1e+09
1e+07
1e+05
400
300
200
100
0
OTUID
Streptococcus_338
Rothia_619
Streptococcus_693
Uncultured_streptococcus_sp._73
Prevotella_1086
Brevibacterium_21
Veillonella_821
Granulicatella_817
Prevotella_histicola_137
Parvimonas_389
Other

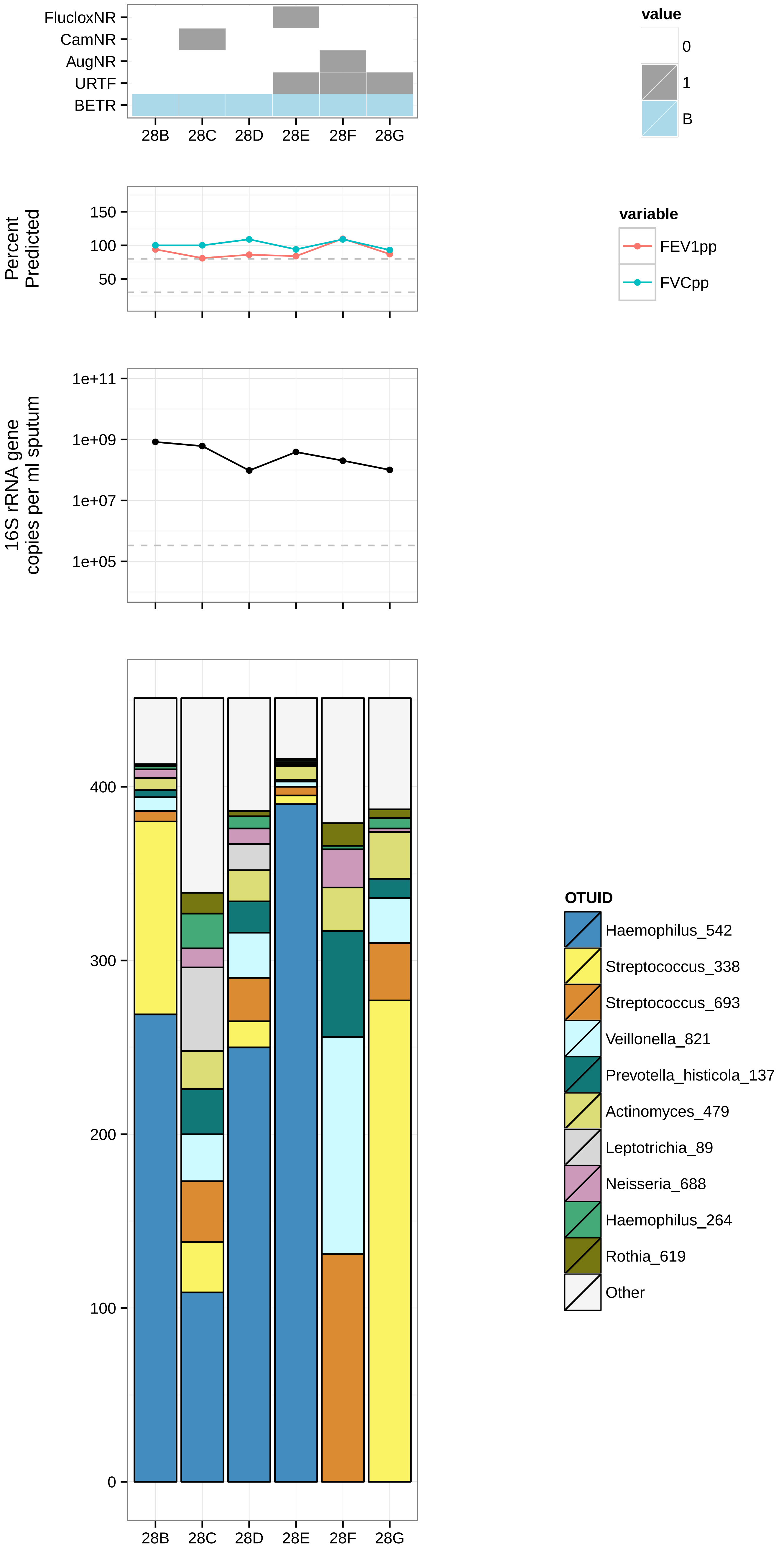
FlucloxNR
CamNR
AugNR
URTF
BETR
28B
28C
28D
28E
28F
28G
value
0
1
B
Percent Predicted
150
100
50
variable
FEV1pp
FVCpp
16S rRNA gene copies per ml sputum
1e+11
1e+09
1e+07
1e+05
400
300
200
100
0
OTUID
Haemophilus_542
Streptococcus_338
Streptococcus_693
Veillonella_821
Prevotella_histicola_137
Actinomyces_479
Leptotrichia_89
Neisseria_688
Haemophilus_264
Rothia_619
Other

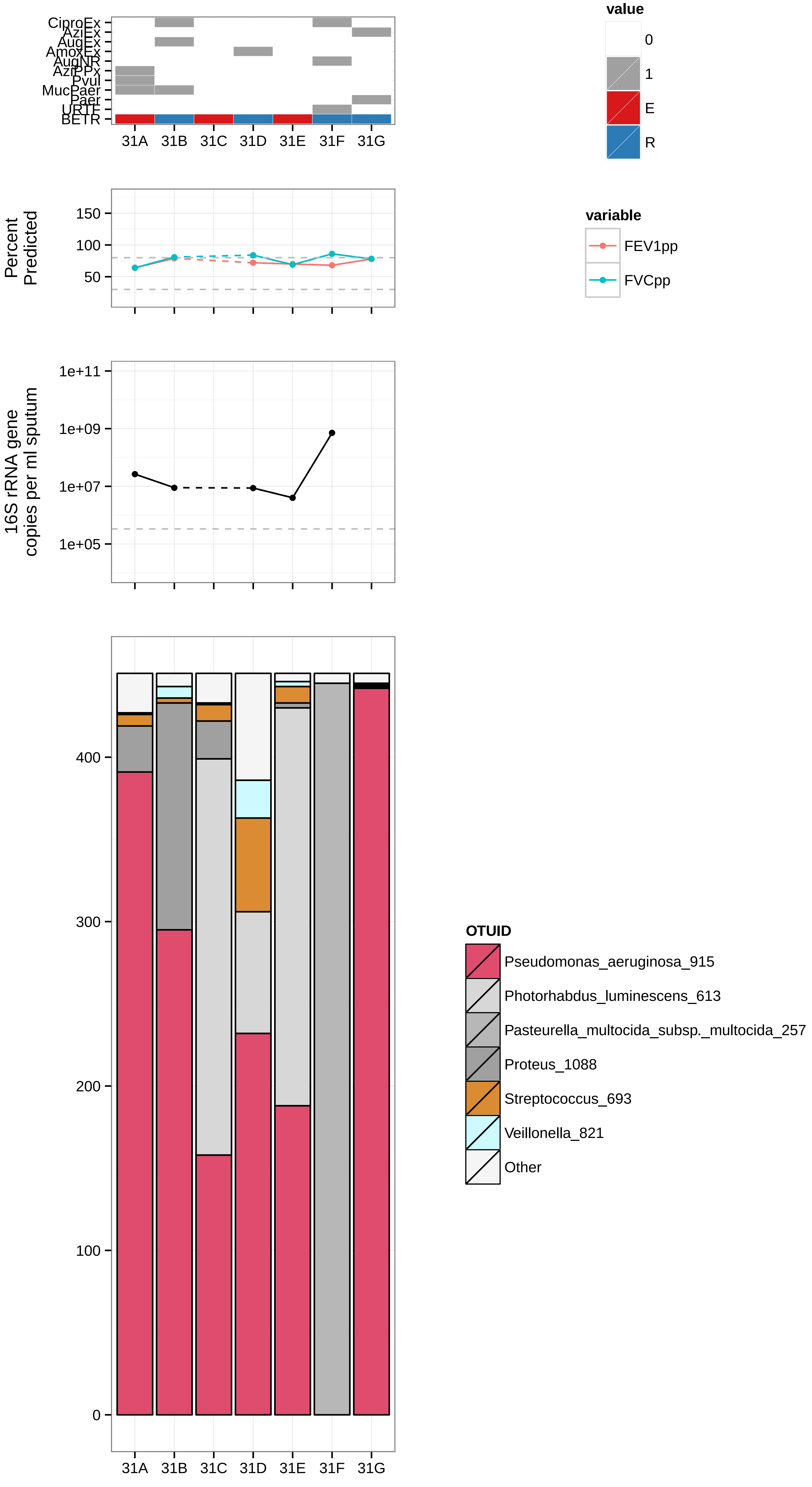

CiproEx
AziEx
AugEx
AmoxEx
AugNR
AziPPx
Pvul
MucPaer
Paer
URTE
BETR
31A
31B
31C
31D
31E
31F
31G
value
0
1
E
R
Percent Predicted
150
100
50
variable
FEV1pp
FVCpp
16S rRNA gene copies per ml sputum
1e+11
1e+09
1e+07
1e+05
400
300
200
100
0
OTUID
Pseudomonas_aeruginosa_915
Photorhabdus_luminescens_613
Pasteurella_multocida_subsp._multocida_257
Proteus_1088
Streptococcus_693
Veillonella_821
Other

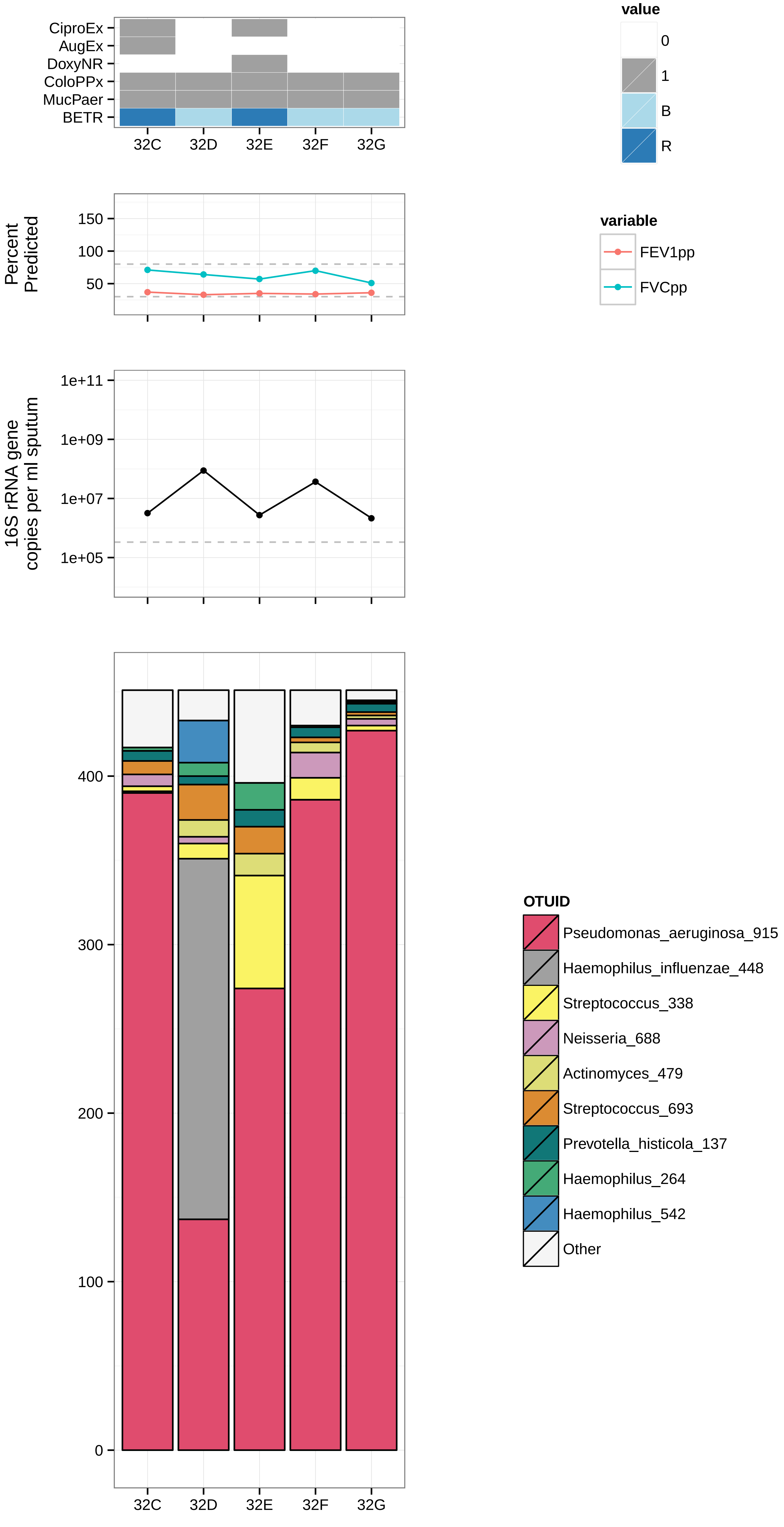
CiproEx
AugEx
DoxyNR
ColoPPx
MucPaer
BETR
32C
32D
32E
32F
32G
value
0
1
B
R
Percent Predicted
150
100
50
variable
FEV1pp
FVCpp
16S rRNA gene copies per ml sputum
1e+11
1e+09
1e+07
1e+05
400
300
200
100
0
OTUID
Pseudomonas_aeruginosa_915
Haemophilus_influenzae_448
Streptococcus_338
Neisseria_688
Actinomyces_479
Streptococcus_693
Prevotella_histicola_137
Haemophilus_264
Haemophilus_542
Other

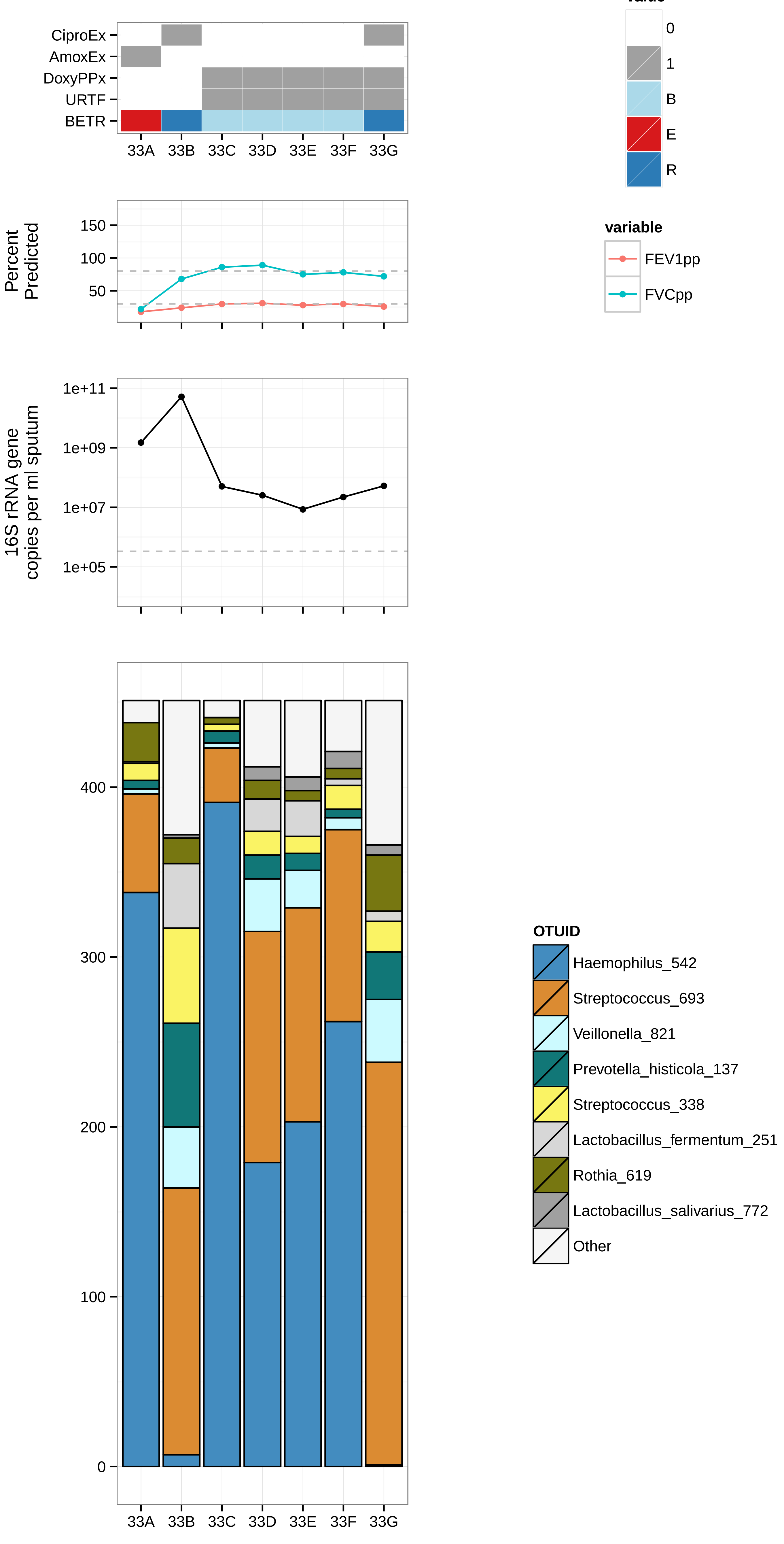

value
0
1
B
E
R
CiproEx
AmoxEx
DoxyPPx
URTF
BETR
33A
33B
33C
33D
33E
33F
33G
Percent Predicted
150
100
50
variable
FEV1pp
FVCpp
16S rRNA gene copies per ml sputum
1e+11
1e+09
1e+07
1e+05
400
300
200
100
0
OTUID
Haemophilus_542
Streptococcus_693
Veillonella_821
Prevotella_histicola_137
Streptococcus_338
Lactobacillus_fermentum_251
Rothia_619
Lactobacillus_salivarius_772
Other

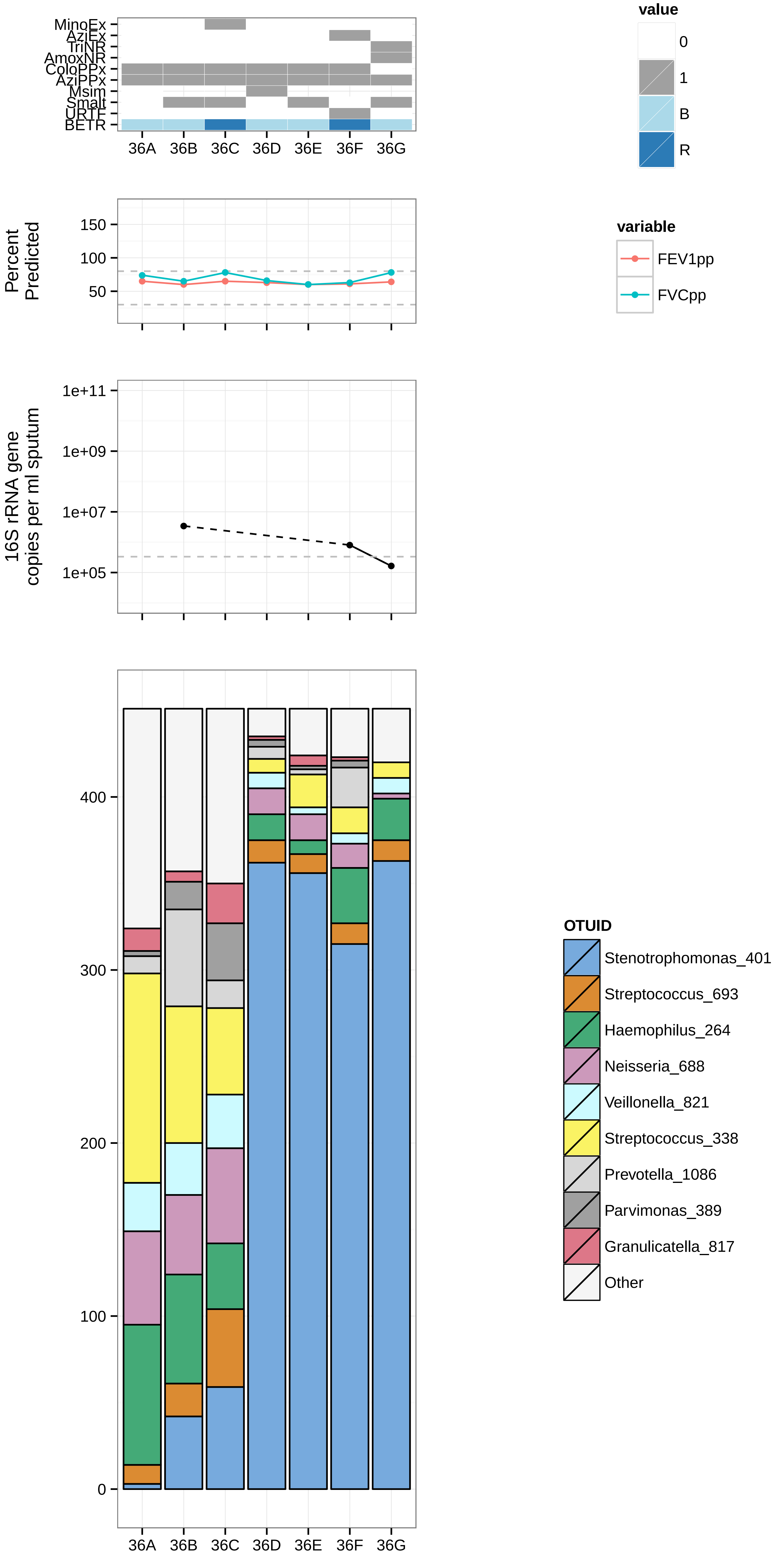

MinoEx
AziEx
TriNR
AmoxNR
ColoPPx
AziPPx
Msim
Smalt
URTF
BETR
36A 36B 36C 36D 36E 36F 36G
value
0
1
B
R
Percent Predicted
150
100
50
variable
FEV1pp
FVCpp
16S rRNA gene copies per ml sputum
1e+11
1e+09
1e+07
1e+05
400
300
200
100
0
OTUID
Stenotrophomonas_401
Streptococcus_693
Haemophilus_264
Neisseria_688
Veillonella_821
Streptococcus_338
Prevotella_1086
Parvimonas_389
Granulicatella_817
Other

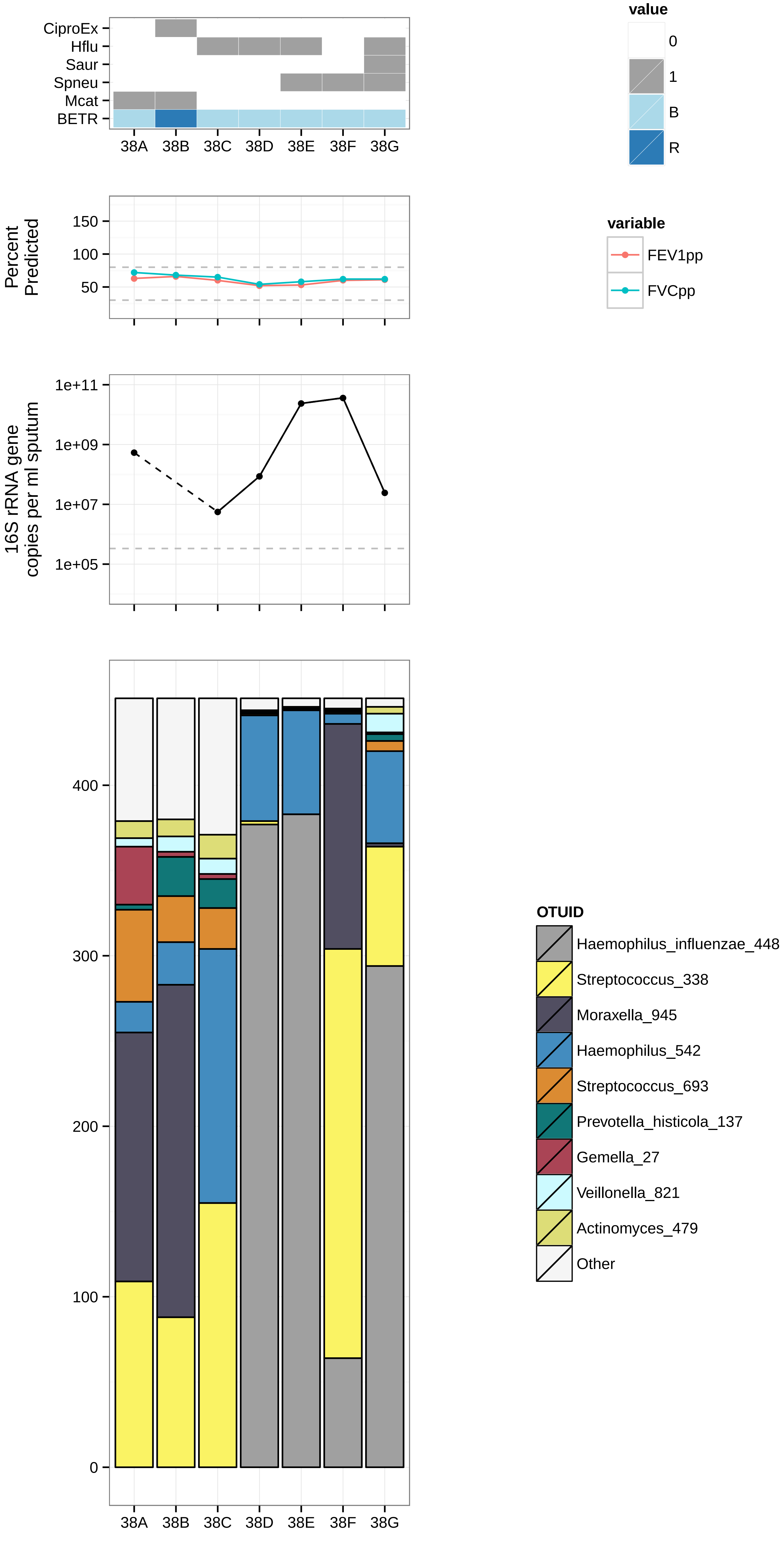
CiproEx
Hflu
Saur
Spneu
Mcat
BETR
38A
38B
38C
38D
38E
38F
38G
value
0
1
B
R
Percent Predicted
150
100
50
variable
FEV1pp
FVCpp
16S rRNA gene copies per ml sputum
1e+11
1e+09
1e+07
1e+05
400
300
200
100
0
OTUID
Haemophilus_influenzae_448
Streptococcus_338
Moraxella_945
Haemophilus_542
Streptococcus_693
Prevotella_histicola_137
Gemella_27
Veillonella_821
Actinomyces_479
Other

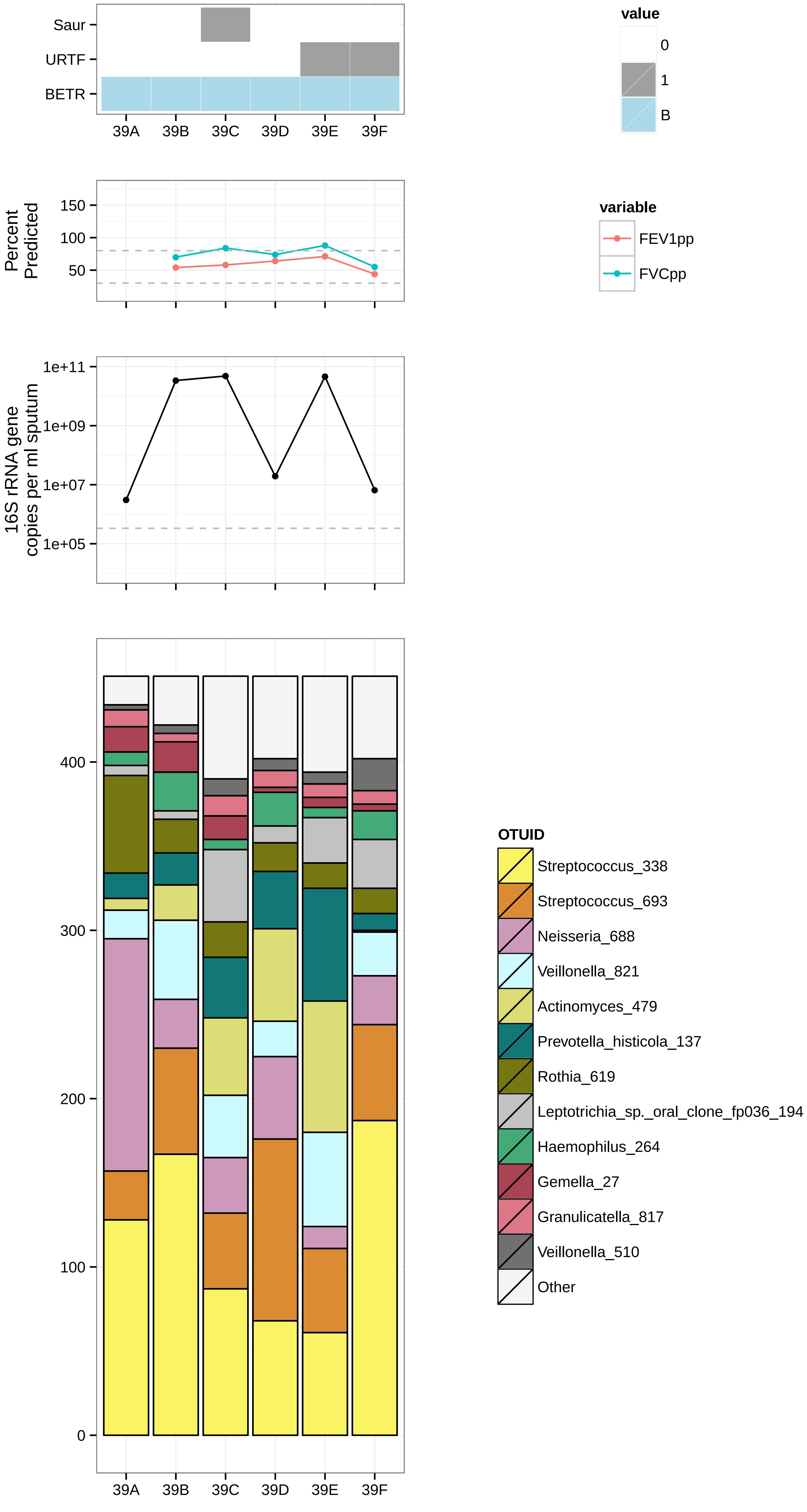

Saur
URTF
BETR
39A
39B
39C
39D
39E
39F
value
0
1
B
Percent Predicted
150
100
50
variable
FEV1pp
FVCpp
16S rRNA gene copies per ml sputum
1e+11
1e+09
1e+07
1e+05
400
300
200
100
0
OTUID
Streptococcus_338
Streptococcus_693
Neisseria_688
Veillonella_821
Actinomyces_479
Prevotella_histicola_137
Rothia_619
Leptotrichia_sp._oral_clone_fp036_194
Haemophilus_264
Gemella_27
Granulicatella_817
Veillonella_510
Other

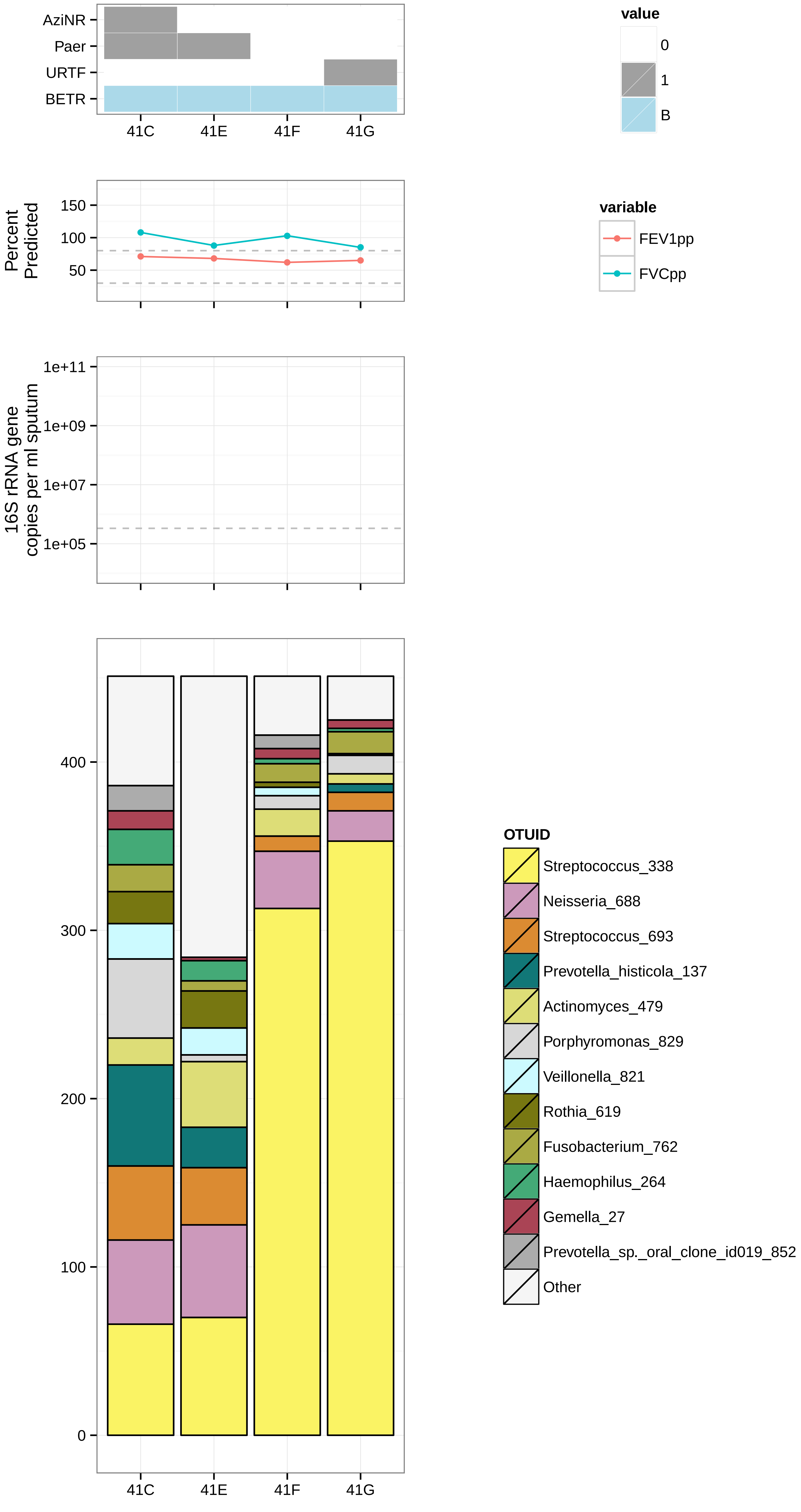

value
0
1
B
AziNR
Paer
URTF
BETR
41C
41E
41F
41G
Percent Predicted
150
100
50
variable
FEV1pp
FVCpp
16S rRNA gene copies per ml sputum
1e+11
1e+09
1e+07
1e+05
400
300
200
100
0
OTUID
Streptococcus_338
Neisseria_688
Streptococcus_693
Prevotella_histicola_137
Actinomyces_479
Porphyromonas_829
Veillonella_821
Rothia_619
Fusobacterium_762
Haemophilus_264
Gemella_27
Prevotella_sp._oral_clone_id019_852
Other

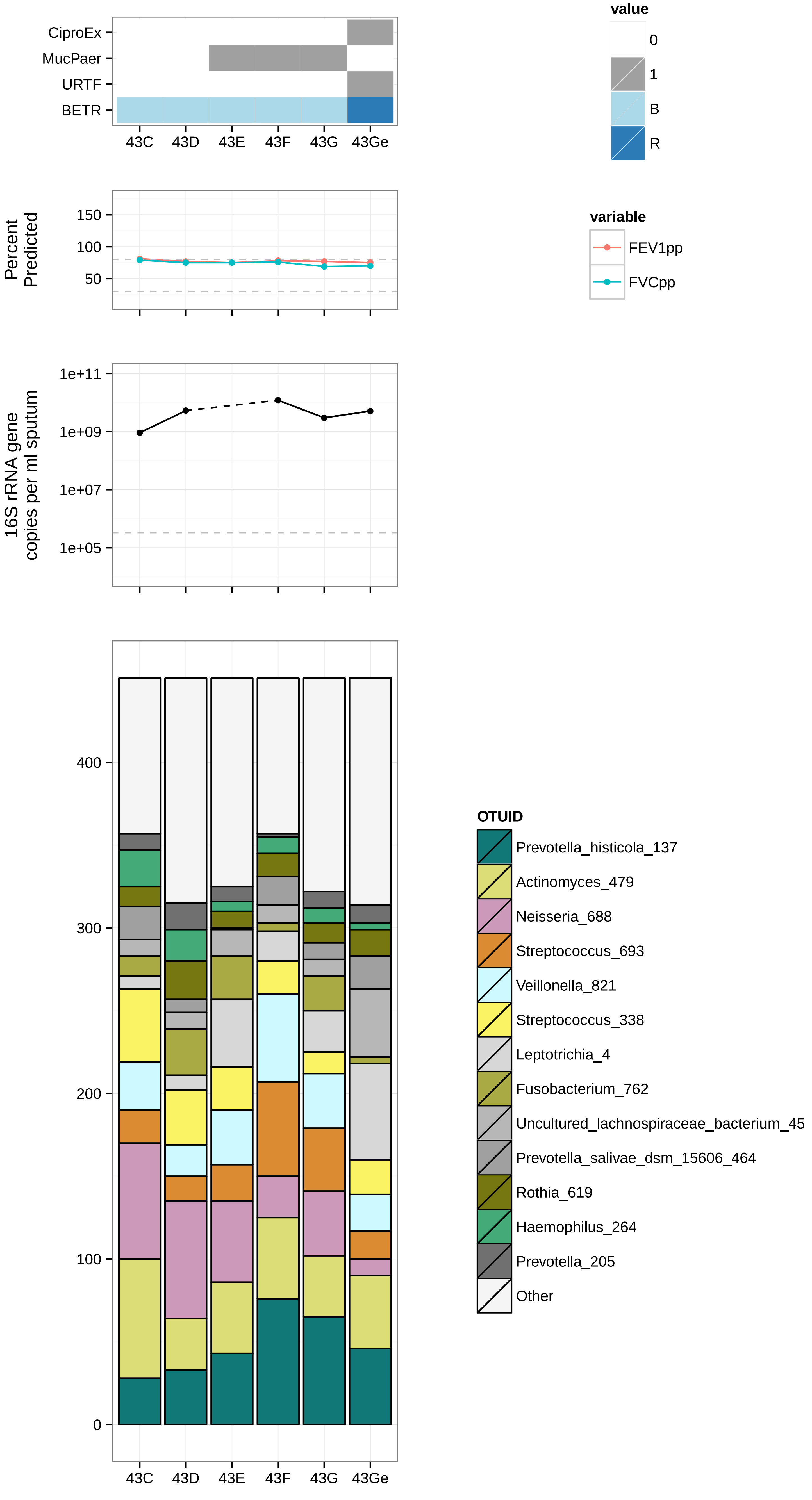
CiproEx
MucPaer
URTF
BETR
43C
43D
43E
43F
43G
43Ge
value
0
1
B
R
Percent Predicted
150
100
50
variable
FEV1pp
FVCpp
16S rRNA gene copies per ml sputum
1e+11
1e+09
1e+07
1e+05
400
300
200
100
0
OTUID
Prevotella_histicola_137
Actinomyces_479
Neisseria_688
Streptococcus_693
Veillonella_821
Streptococcus_338
Leptotrichia_4
Fusobacterium_762
Uncultured_lachnospiraceae_bacterium_45
Prevotella_salivae_dsm_15606_464
Rothia_619
Haemophilus_264
Prevotella_205
Other

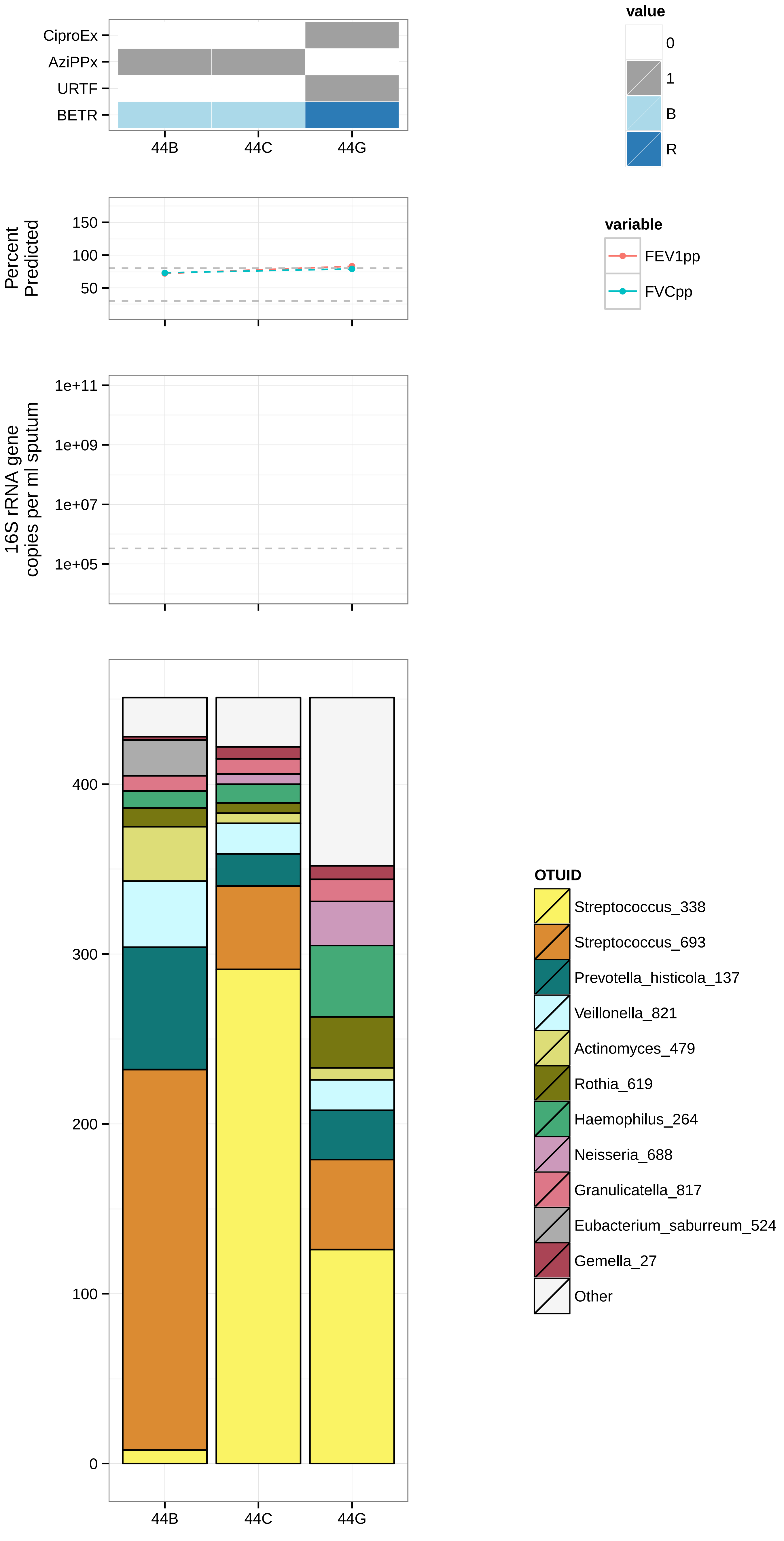

CiproEx
AziPPx
URTF
BETR
44B
44C
44G
value
0
1
B
R
Percent Predicted
150
100
50
variable
FEV1pp
FVCpp
16S rRNA gene copies per ml sputum
1e+11
1e+09
1e+07
1e+05
400
300
200
100
0
OTUID
Streptococcus_338
Streptococcus_693
Prevotella_histicola_137
Veillonella_821
Actinomyces_479
Rothia_619
Haemophilus_264
Neisseria_688
Granulicatella_817
Eubacterium_saburreum_524
Gemella_27
Other

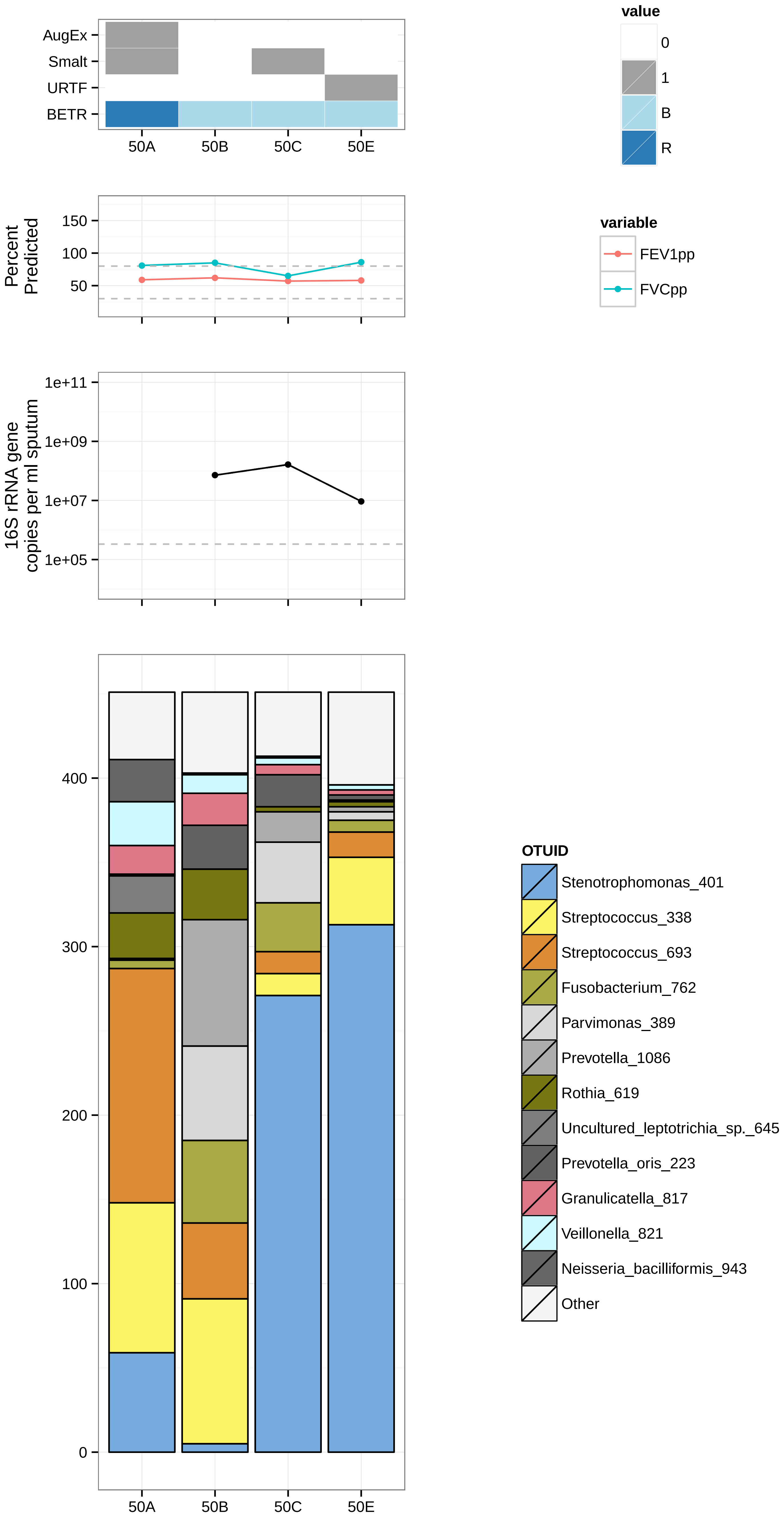

AugEx
Smalt
URTF
BETR
50A
50B
50C
50E
value
0
1
B
R
Percent Predicted
150
100
50
variable
FEV1pp
FVCpp
16S rRNA gene copies per ml sputum
1e+11
1e+09
1e+07
1e+05
400
300
200
100
0
OTUID
Stenotrophomonas_401
Streptococcus_338
Streptococcus_693
Fusobacterium_762
Parvimonas_389
Prevotella_1086
Rothia_619
Uncultured_leptotrichia_sp._645
Prevotella_oris_223
Granulicatella_817
Veillonella_821
Neisseria_bacilliformis_943
Other

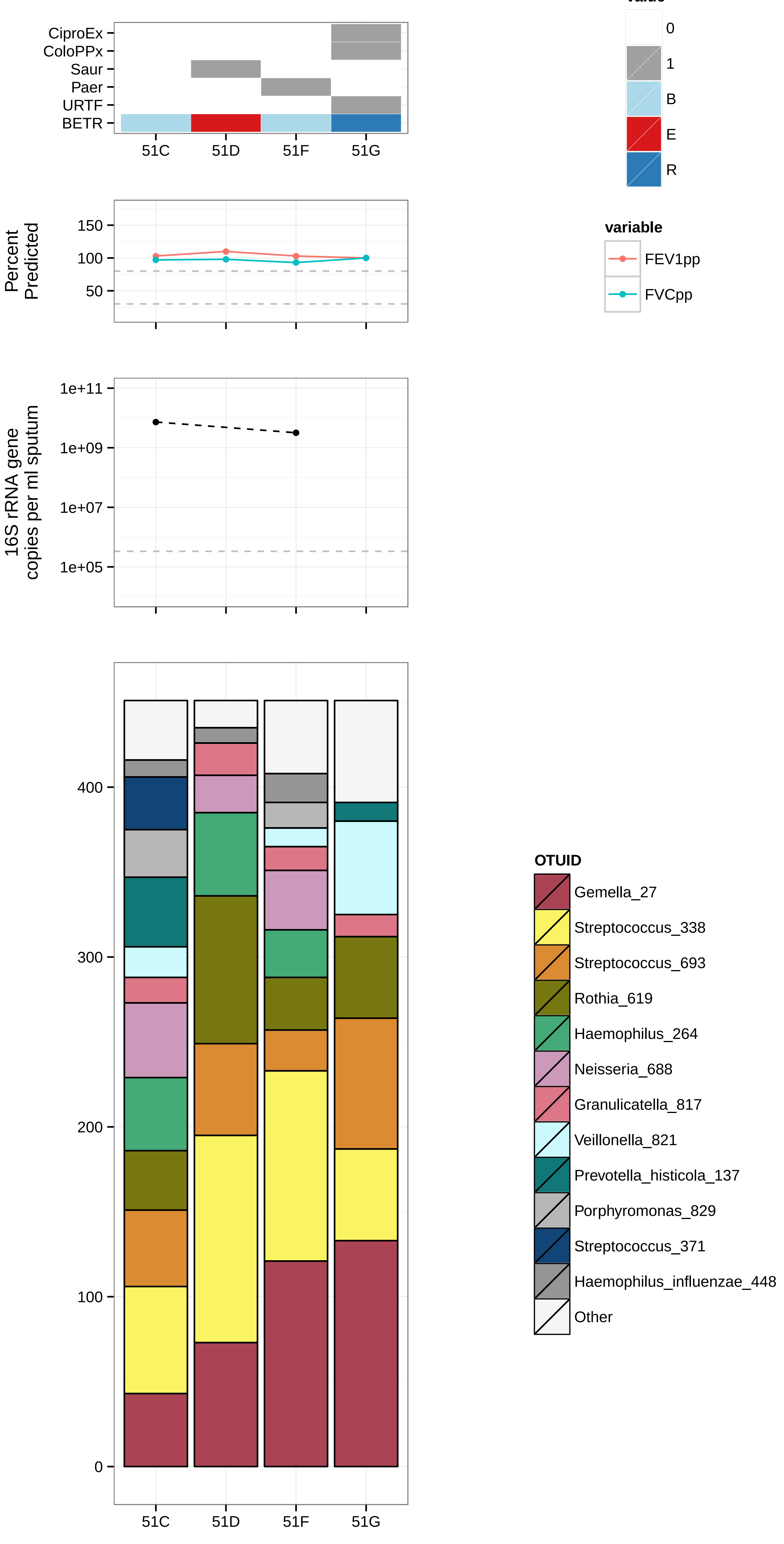

CiproEx
ColoPPx
Saur
Paer
URTF
BETR
51C
51D
51F
51G
value
0
1
B
E
R
Percent Predicted
150
100
50
variable
FEV1pp
FVCpp
16S rRNA gene copies per ml sputum
1e+11
1e+09
1e+07
1e+05
400
300
200
100
0
OTUID
Gemella_27
Streptococcus_338
Streptococcus_693
Rothia_619
Haemophilus_264
Neisseria_688
Granulicatella_817
Veillonella_821
Prevotella_histicola_137
Porphyromonas_829
Streptococcus_371
Haemophilus_influenzae_448
Other

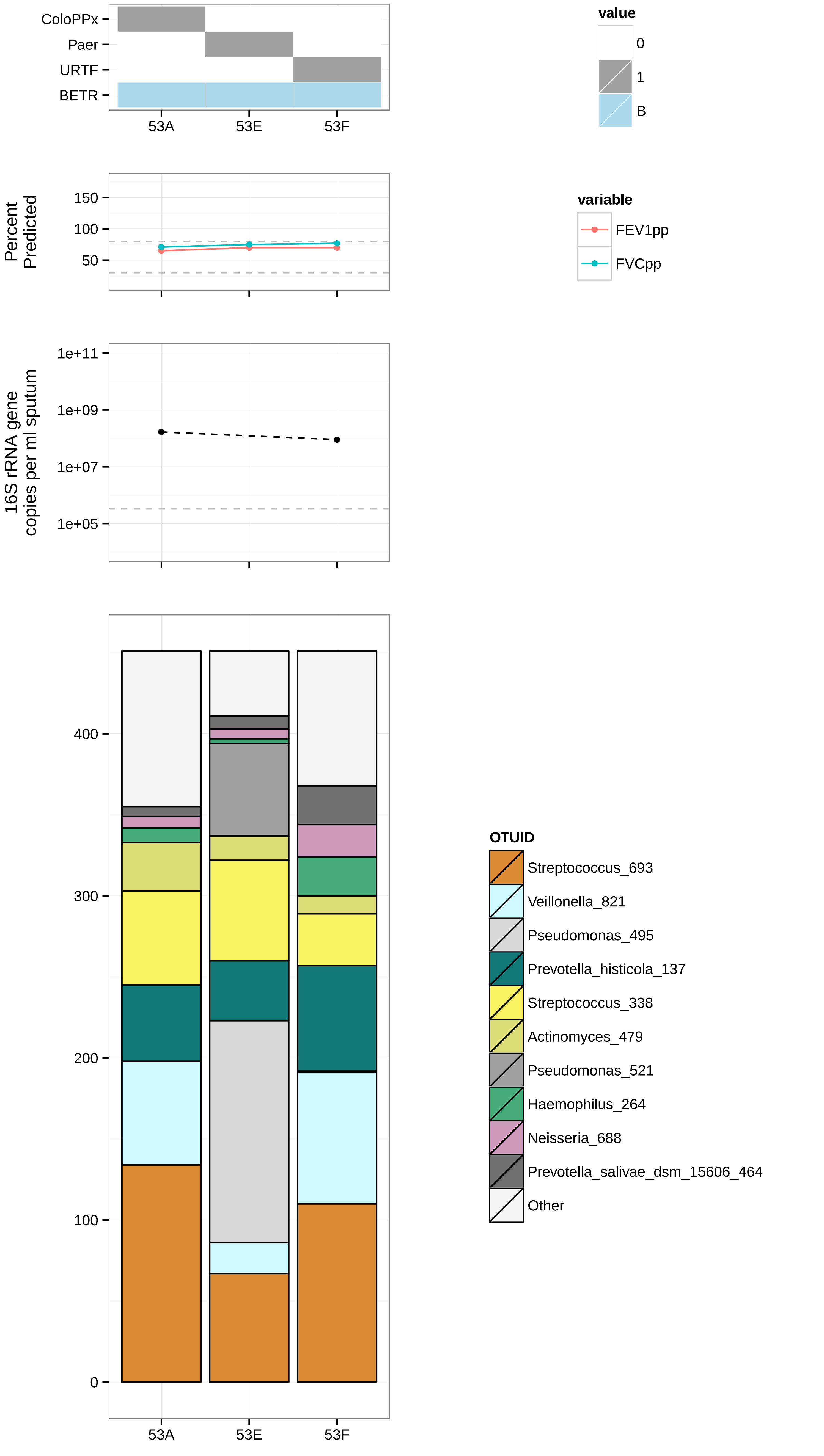

ColoPPx
Paer
URTF
BETR
53A
53E
53F
value
0
1
B
Percent Predicted
150
100
50
variable
FEV1pp
FVCpp
16S rRNA gene copies per ml sputum
1e+11
1e+09
1e+07
1e+05
400
300
200
100
0
OTUID
Streptococcus_693
Veillonella_821
Pseudomonas_495
Prevotella_histicola_137
Streptococcus_338
Actinomyces_479
Pseudomonas_521
Haemophilus_264
Neisseria_688
Prevotella_salivae_dsm_15606_464
Other

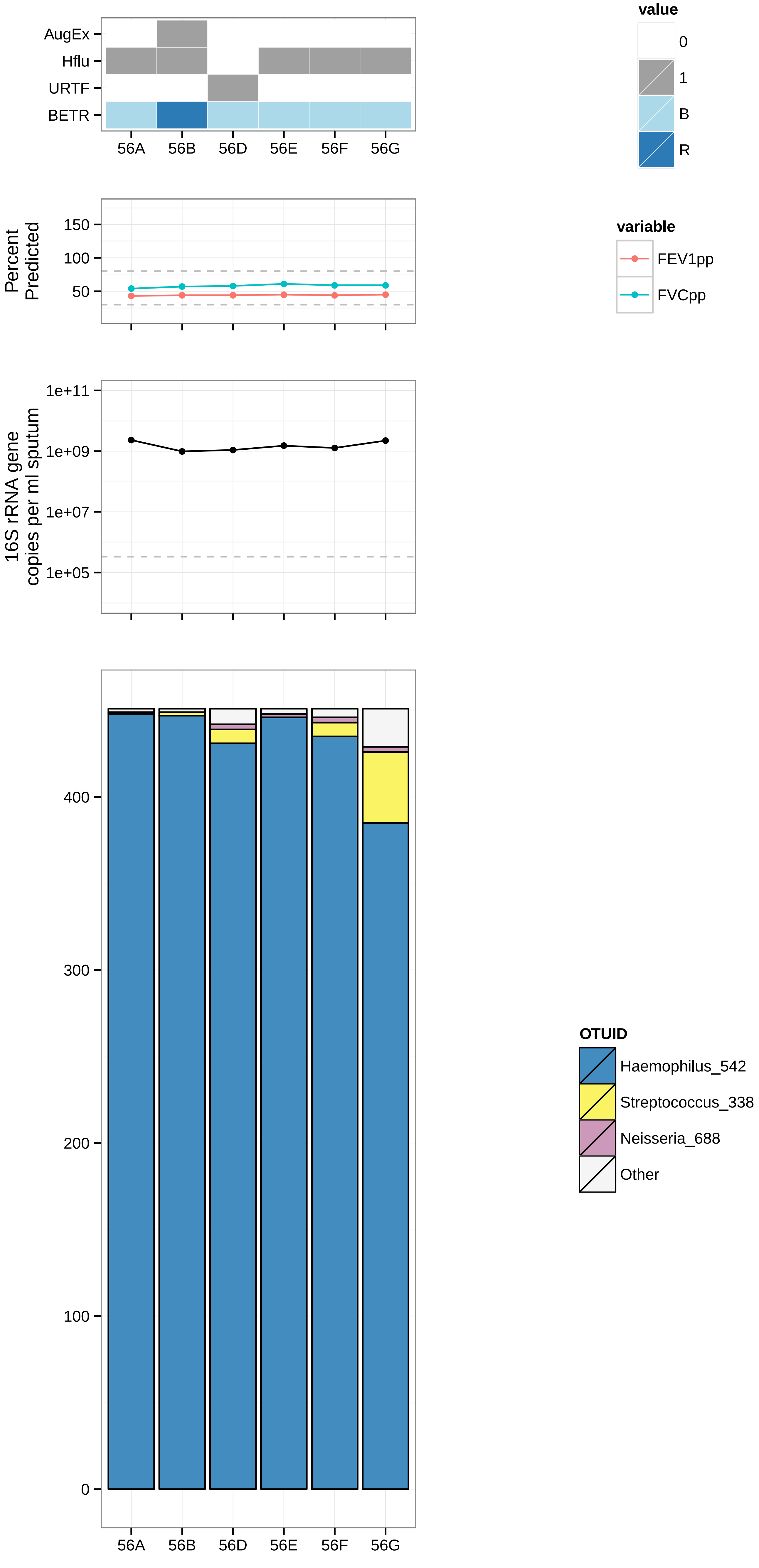
AugEx
Hflu
URTF
BETR
56A
56B
56D
56E
56F
56G
value
0
1
B
R
Percent Predicted
150
100
50
variable
FEV1pp
FVCpp
16S rRNA gene copies per ml sputum
1e+11
1e+09
1e+07
1e+05
400
300
200
100
0
OTUID
Haemophilus_542
Streptococcus_338
Neisseria_688
Other

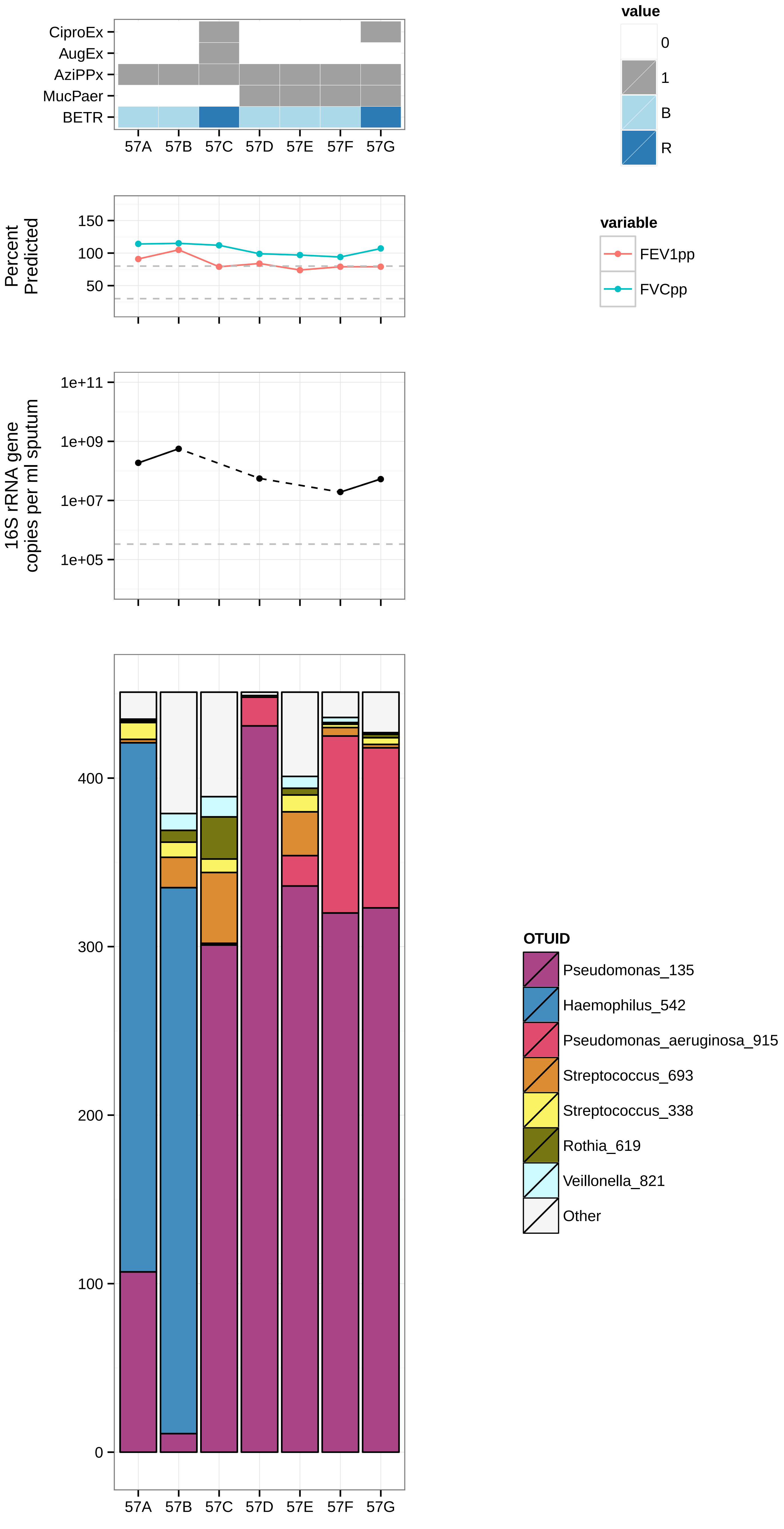

CiproEx
AugEx
AziPPx
MucPaer
BETR
57A
57B
57C
57D
57E
57F
57G
value
0
1
B
R
Percent
Predicted
150
100
50
variable
FEV1pp
FVCpp
16S rRNA gene
copies per ml sputum
1e+11
1e+09
1e+07
1e+05
400
300
200
100
0
OTUID
Pseudomonas_135
Haemophilus_542
Pseudomonas_aeruginosa_915
Streptococcus_693
Streptococcus_338
Rothia_619
Veillonella_821
Other

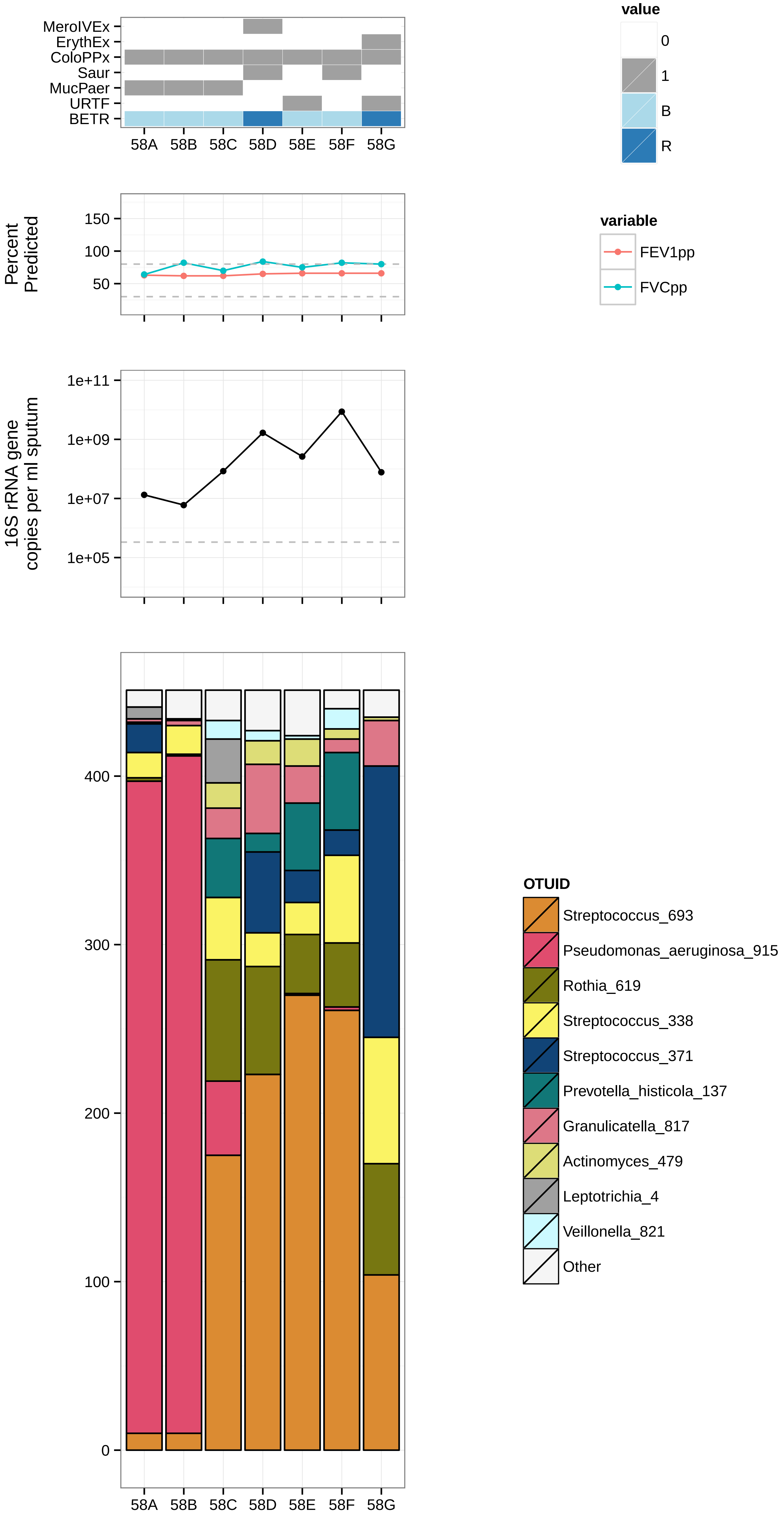

MeroIVEx
ErythEx
ColoPPx
Saur
MucPaer
URTF
BETR
58A
58B
58C
58D
58E
58F
58G
value
0
1
B
R
Percent Predicted
150
100
50
variable
FEV1pp
FVCpp
16S rRNA gene copies per ml sputum
1e+11
1e+09
1e+07
1e+05
400
300
200
100
0
OTUID
Streptococcus_693
Pseudomonas_aeruginosa_915
Rothia_619
Streptococcus_338
Streptococcus_371
Prevotella_histicola_137
Granulicatella_817
Actinomyces_479
Leptotrichia_4
Veillonella_821
Other

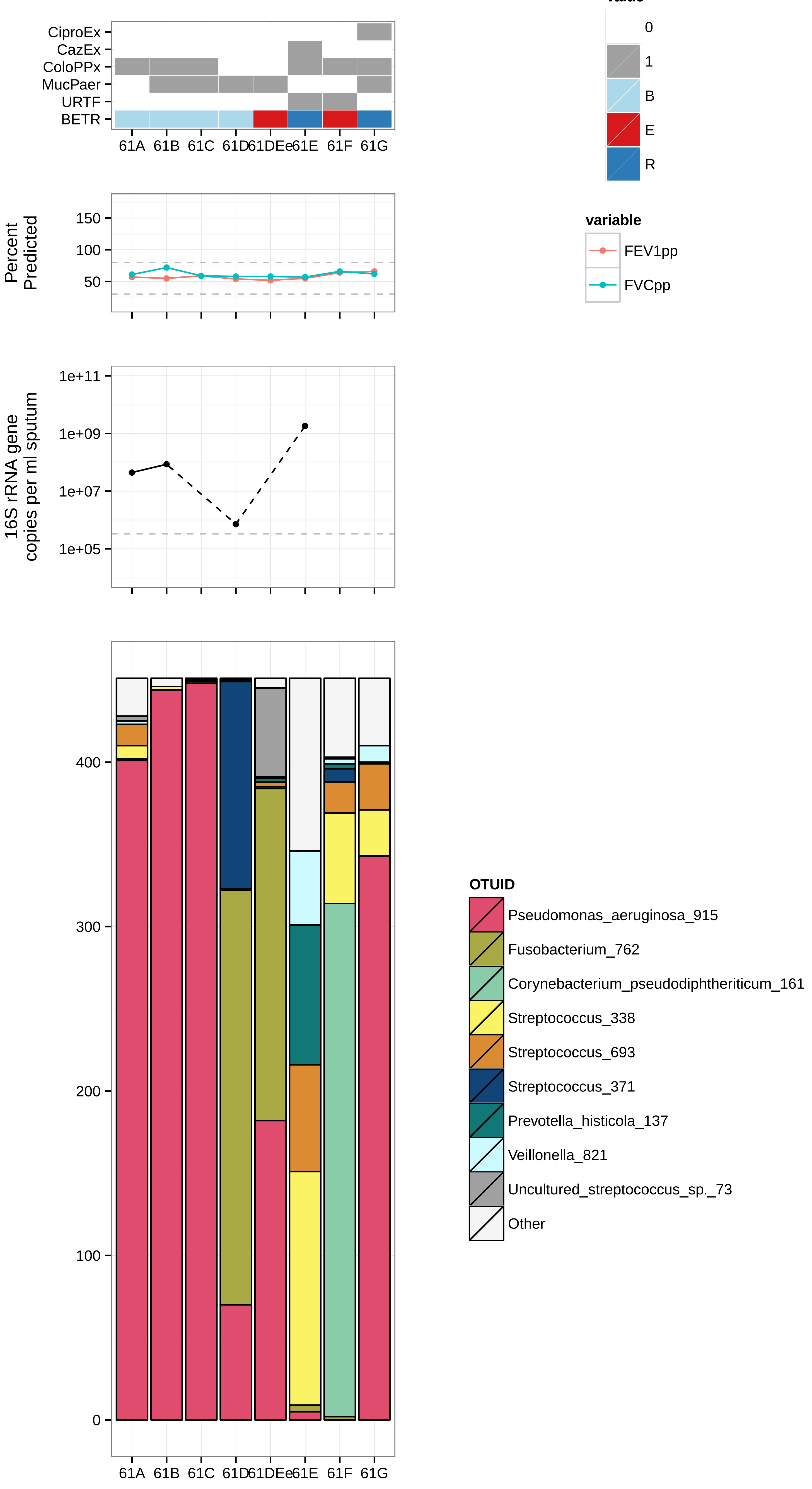

value
0
1
B
E
R
CiproEx
CazEx
ColoPPx
MucPaer
URTF
BETR
61A
61B
61C
61D
61DEe
61E
61F
61G
Percent Predicted
150
100
50
variable
FEV1pp
FVCpp
16S rRNA gene copies per ml sputum
1e+11
1e+09
1e+07
1e+05
400
300
200
100
0
OTUID
Pseudomonas_aeruginosa_915
Fusobacterium_762
Corynebacterium_pseudodiphtheriticum_161
Streptococcus_338
Streptococcus_693
Streptococcus_371
Prevotella_histicola_137
Veillonella_821
Uncultured_streptococcus_sp._73
Other

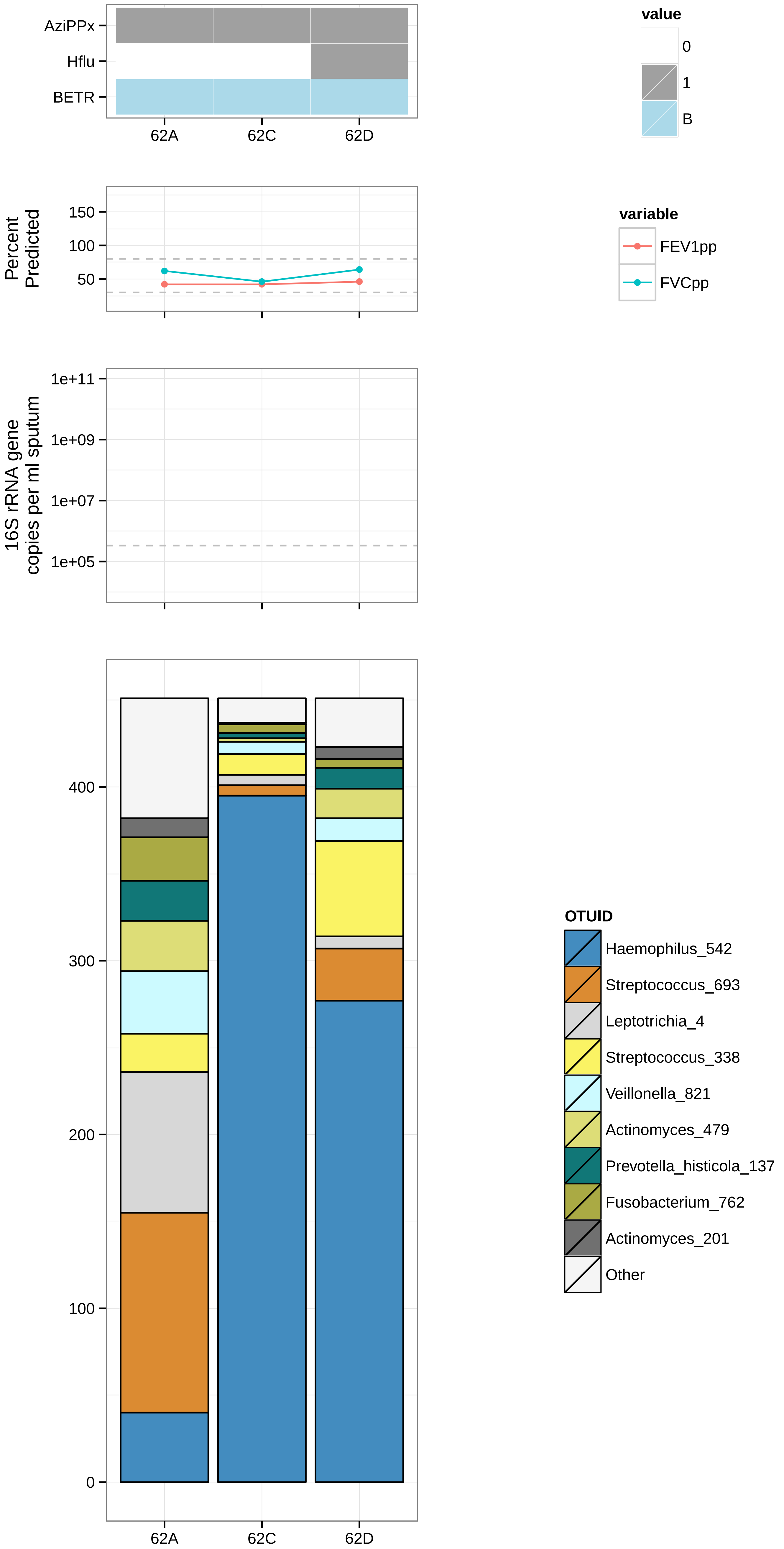

AziPPx
Hflu
BETR
62A
62C
62D
value
0
1
B
Percent Predicted
150
100
50
variable
FEV1pp
FVCpp
16S rRNA gene copies per ml sputum
1e+11
1e+09
1e+07
1e+05
400
300
200
100
0
OTUID
Haemophilus_542
Streptococcus_693
Leptotrichia_4
Streptococcus_338
Veillonella_821
Actinomyces_479
Prevotella_histicola_137
Fusobacterium_762
Actinomyces_201
Other

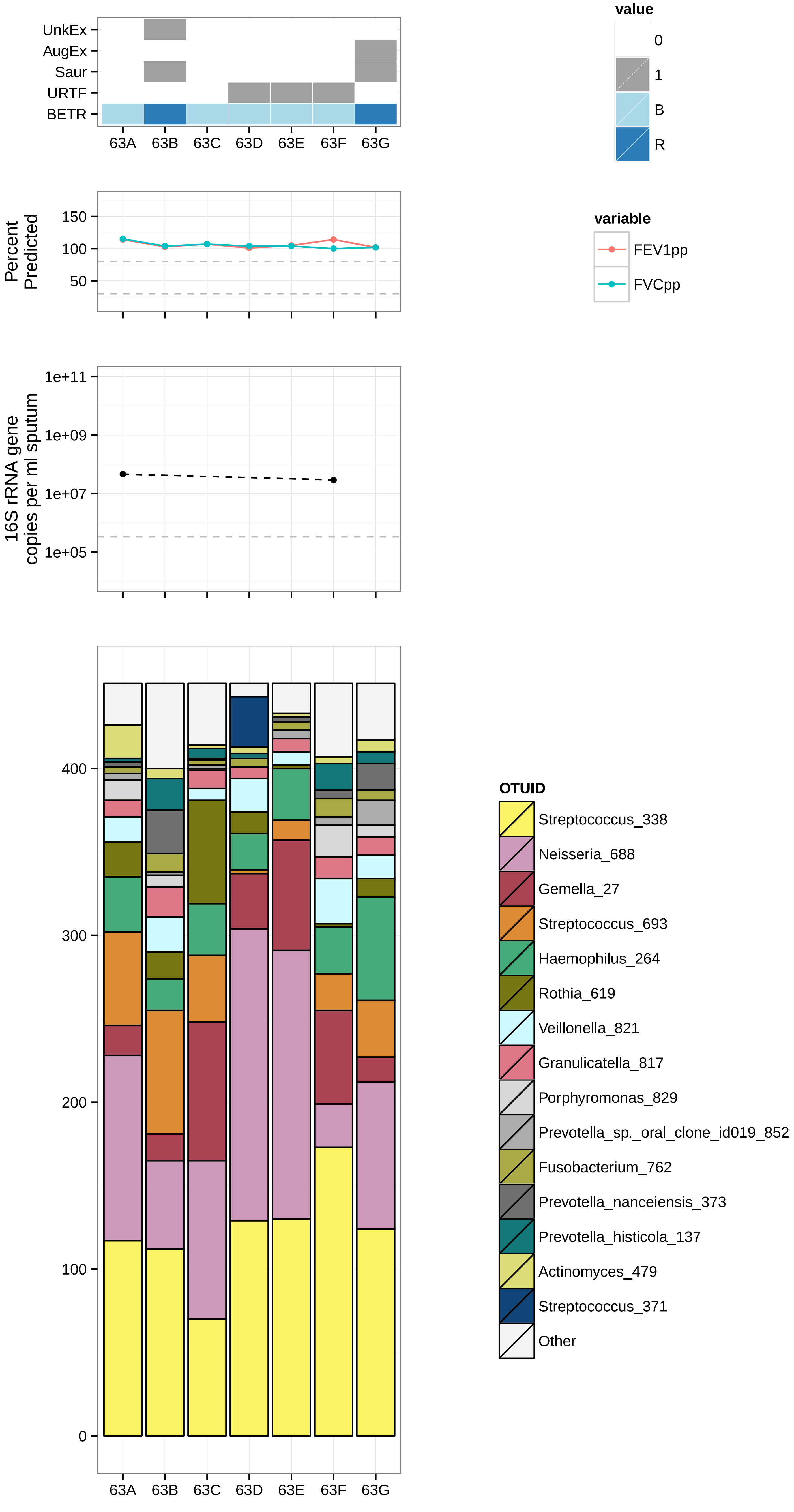

UnkEx
AugEx
Saur
URTF
BETR
63A 63B 63C 63D 63E 63F 63G
value
0
1
B
R
Percent Predicted
150
100
50
variable
FEV1pp
FVCpp
16S rRNA gene copies per ml sputum
1e+11
1e+09
1e+07
1e+05
400
300
200
100
0
OTUID
Streptococcus_338
Neisseria_688
Gemella_27
Streptococcus_693
Haemophilus_264
Rothia_619
Veillonella_821
Granulicatella_817
Porphyromonas_829
Prevotella_sp._oral_clone_id019_852
Fusobacterium_762
Prevotella_nanceiensis_373
Prevotella_histicola_137
Actinomyces_479
Streptococcus_371
Other

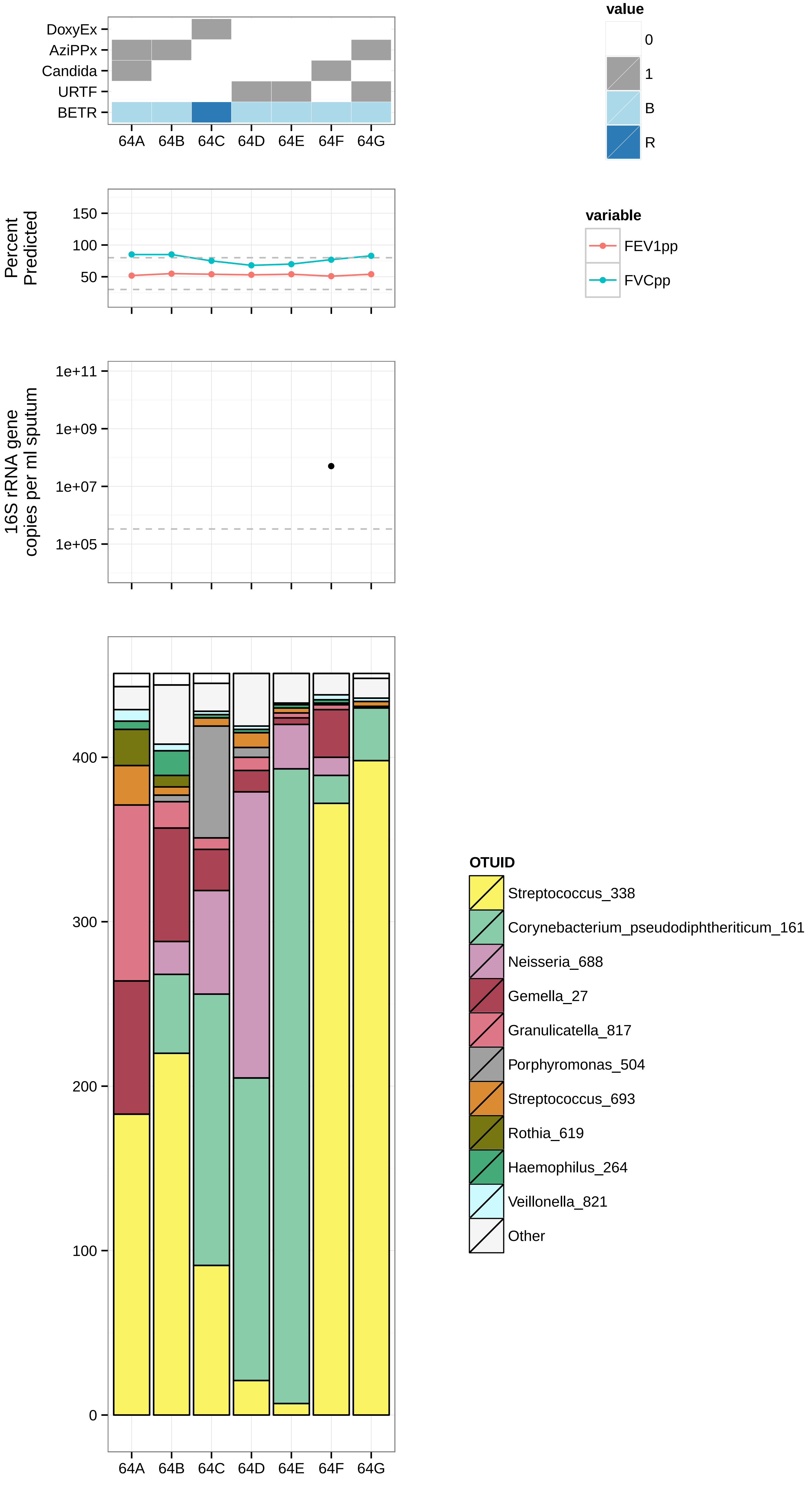

DoxyEx
AziPPx
Candida
URTF
BETR
64A
64B
64C
64D
64E
64F
64G
value
0
1
B
R
Percent Predicted
150
100
50
variable
FEV1pp
FVCpp
16S rRNA gene copies per ml sputum
1e+11
1e+09
1e+07
1e+05
400
300
200
100
0
OTUID
Streptococcus_338
Corynebacterium_pseudodiphtheriticum_161
Neisseria_688
Gemella_27
Granulicatella_817
Porphyromonas_504
Streptococcus_693
Rothia_619
Haemophilus_264
Veillonella_821
Other

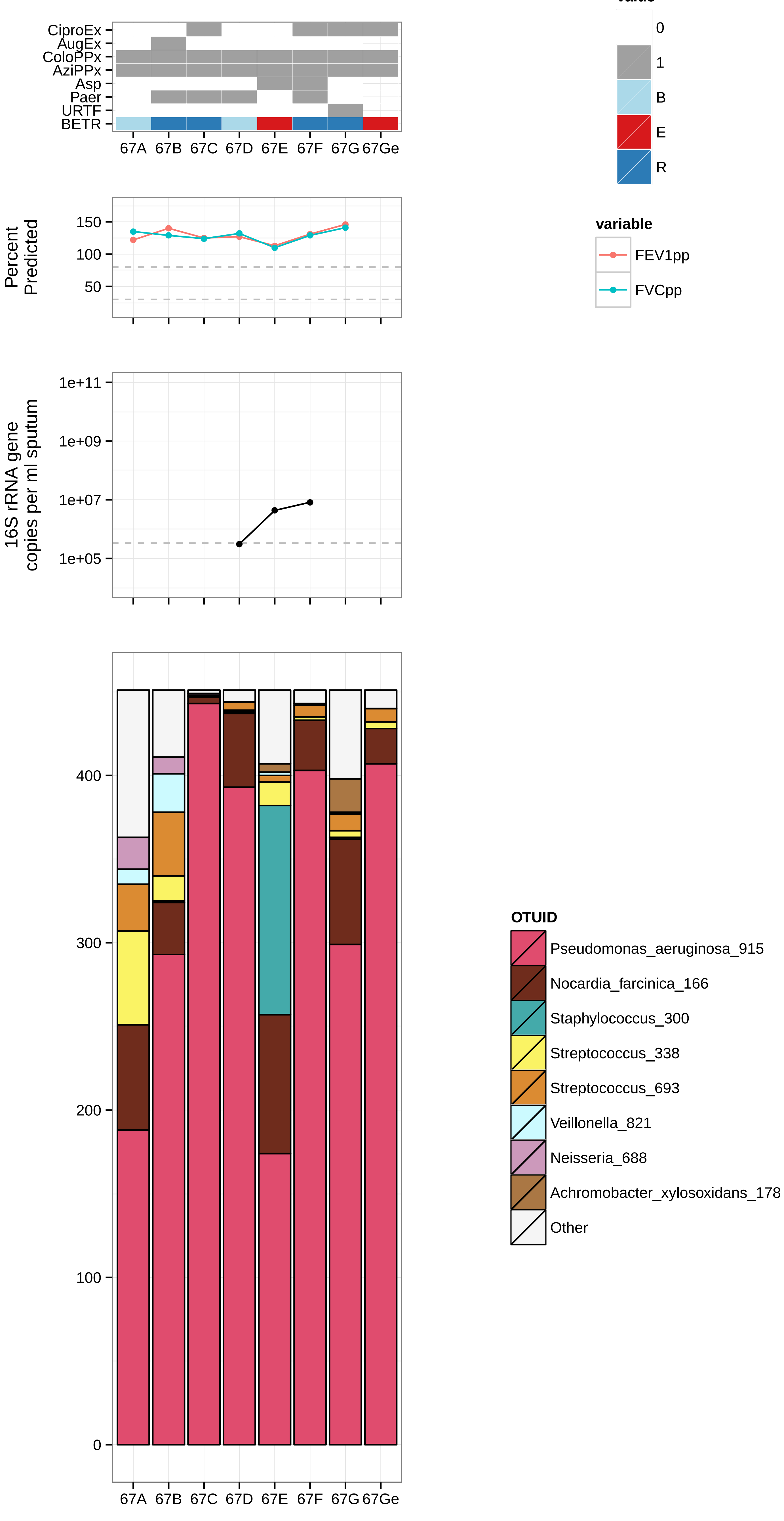
value
0
1
B
E
R
CiproEx
AugEx
ColoPPx
AziPPx
Asp
Paer
URTF
BETR
67A 67B 67C 67D 67E 67F 67G 67Ge
Percent Predicted
150
100
50
variable
FEV1pp
FVCpp
16S rRNA gene copies per ml sputum
1e+11
1e+09
1e+07
1e+05
400
300
200
100
0
OTUID
Pseudomonas_aeruginosa_915
Nocardia_farcinica_166
Staphylococcus_300
Streptococcus_338
Streptococcus_693
Veillonella_821
Neisseria_688
Achromobacter_xylosoxidans_178
Other

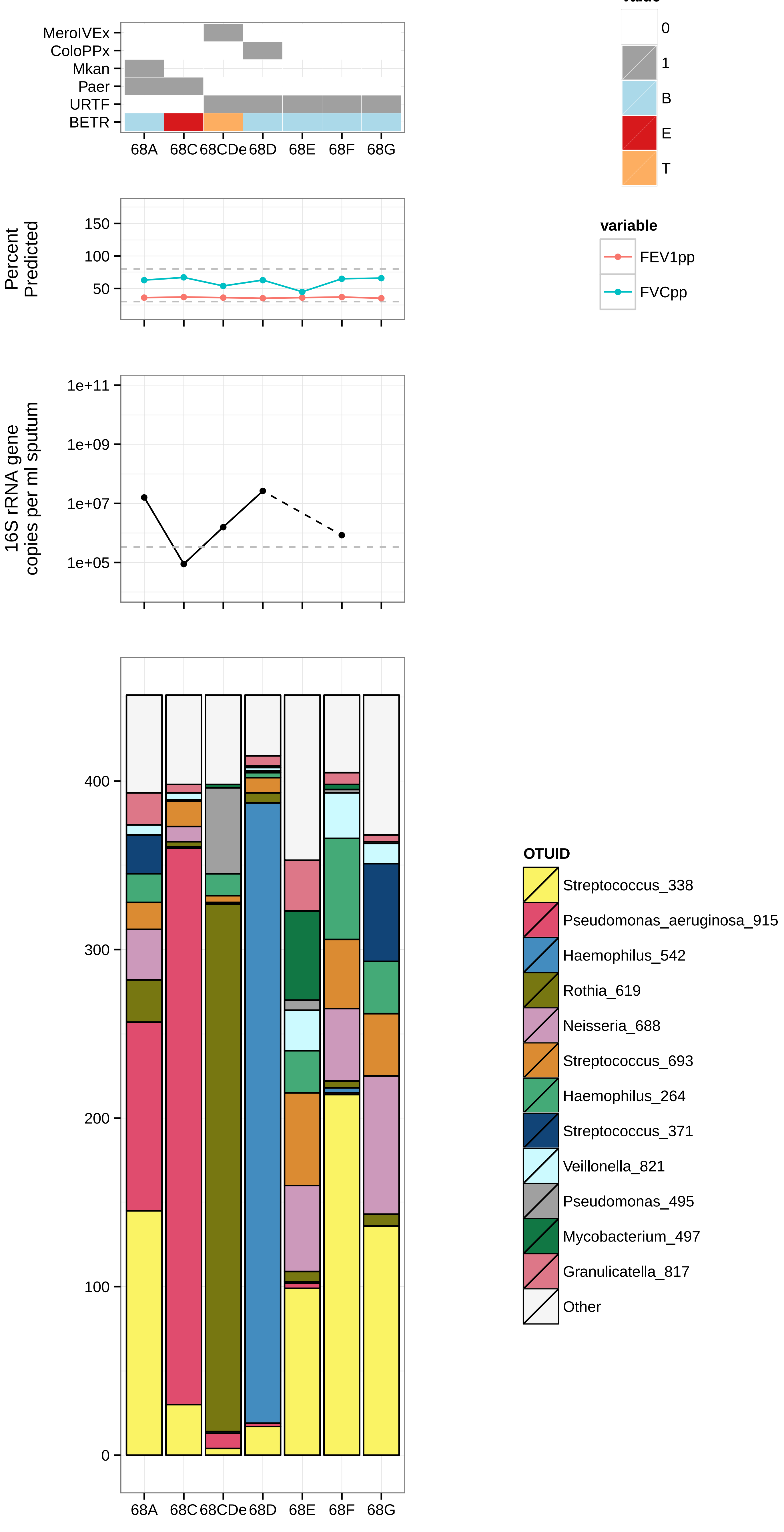

MeroIVEx
ColoPPx
Mkan
Paer
URTF
BETR
68A
68C
68CDe
68D
68E
68F
68G
0
1
B
E
T
Percent Predicted
150
100
50
variable
FEV1pp
FVCpp
16S rRNA gene copies per ml sputum
1e+11
1e+09
1e+07
1e+05
400
300
200
100
0
OTUID
Streptococcus_338
Pseudomonas_aeruginosa_915
Haemophilus_542
Rothia_619
Neisseria_688
Streptococcus_693
Haemophilus_264
Streptococcus_371
Veillonella_821
Pseudomonas_495
Mycobacterium_497
Granulicatella_817
Other

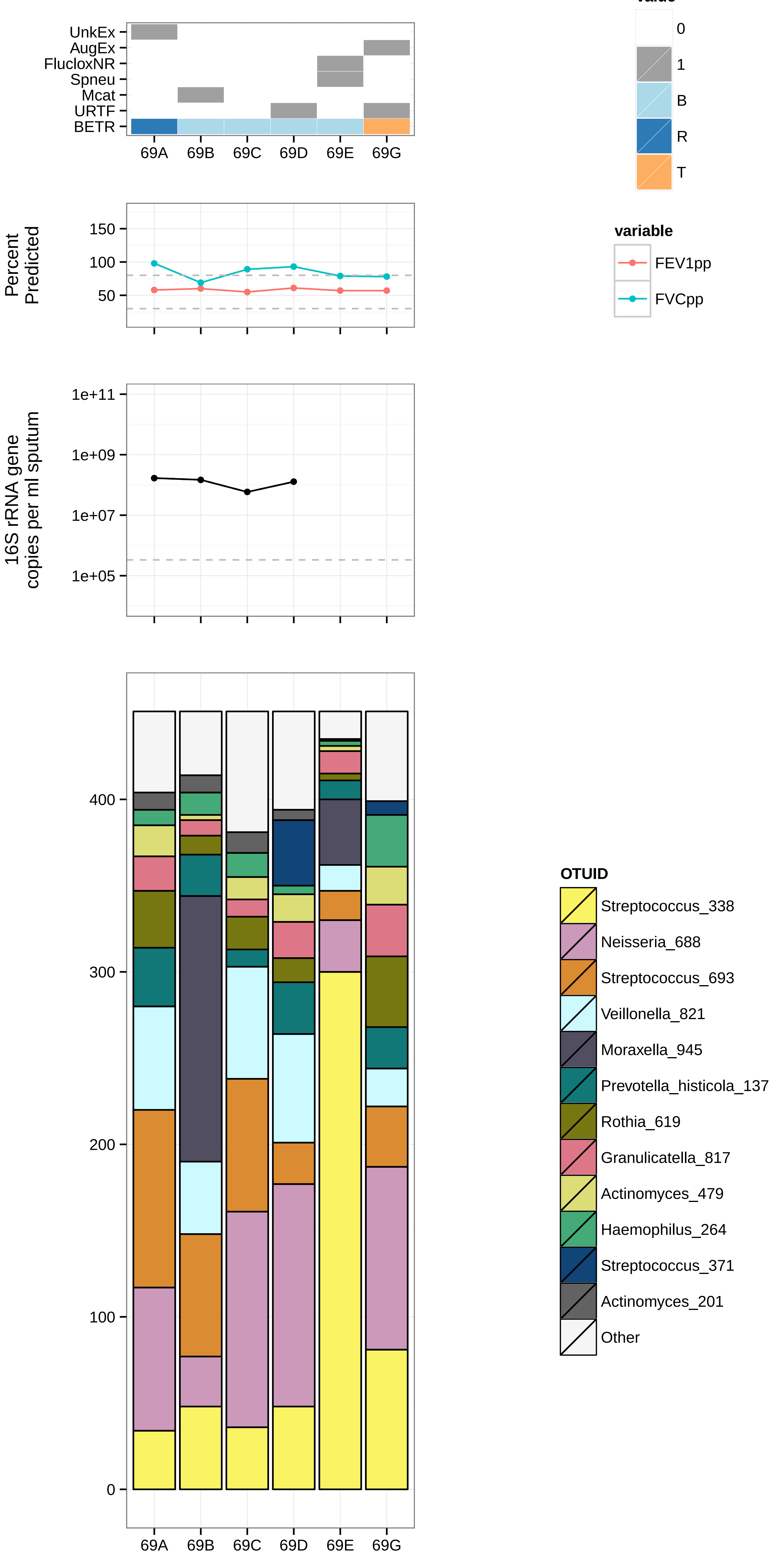

UnkEx
AugEx
FlucloxNR
Spneu
Mcat
URTF
BETR
69A
69B
69C
69D
69E
69G
value
0
1
B
R
T
Percent Predicted
150
100
50
variable
FEV1pp
FVCpp
16S rRNA gene copies per ml sputum
1e+11
1e+09
1e+07
1e+05
400
300
200
100
0
OTUID
Streptococcus_338
Neisseria_688
Streptococcus_693
Veillonella_821
Moraxella_945
Prevotella_histicola_137
Rothia_619
Granulicatella_817
Actinomyces_479
Haemophilus_264
Streptococcus_371
Actinomyces_201
Other

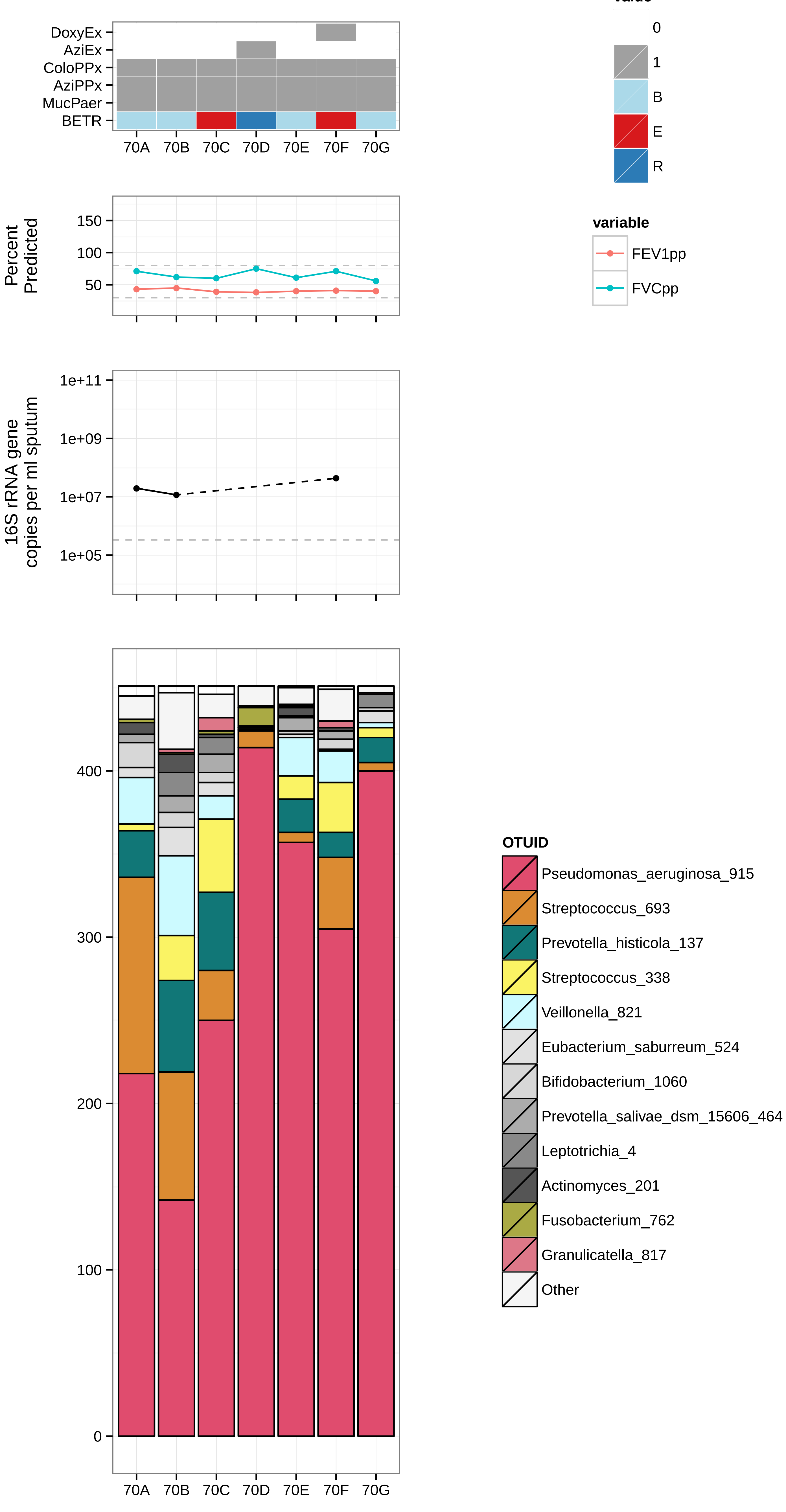
DoxyEx
AziEx
ColoPPx
AziPPx
MucPaer
BETR
70A
70B
70C
70D
70E
70F
70G
0
1
B
E
R
Percent Predicted
150
100
50
variable
FEV1pp
FVCpp
16S rRNA gene copies per ml sputum
1e+11
1e+09
1e+07
1e+05
400
300
200
100
0
OTUID
Pseudomonas_aeruginosa_915
Streptococcus_693
Prevotella_histicola_137
Streptococcus_338
Veillonella_821
Eubacterium_saburreum_524
Bifidobacterium_1060
Prevotella_salivae_dsm_15606_464
Leptotrichia_4
Actinomyces_201
Fusobacterium_762
Granulicatella_817
Other

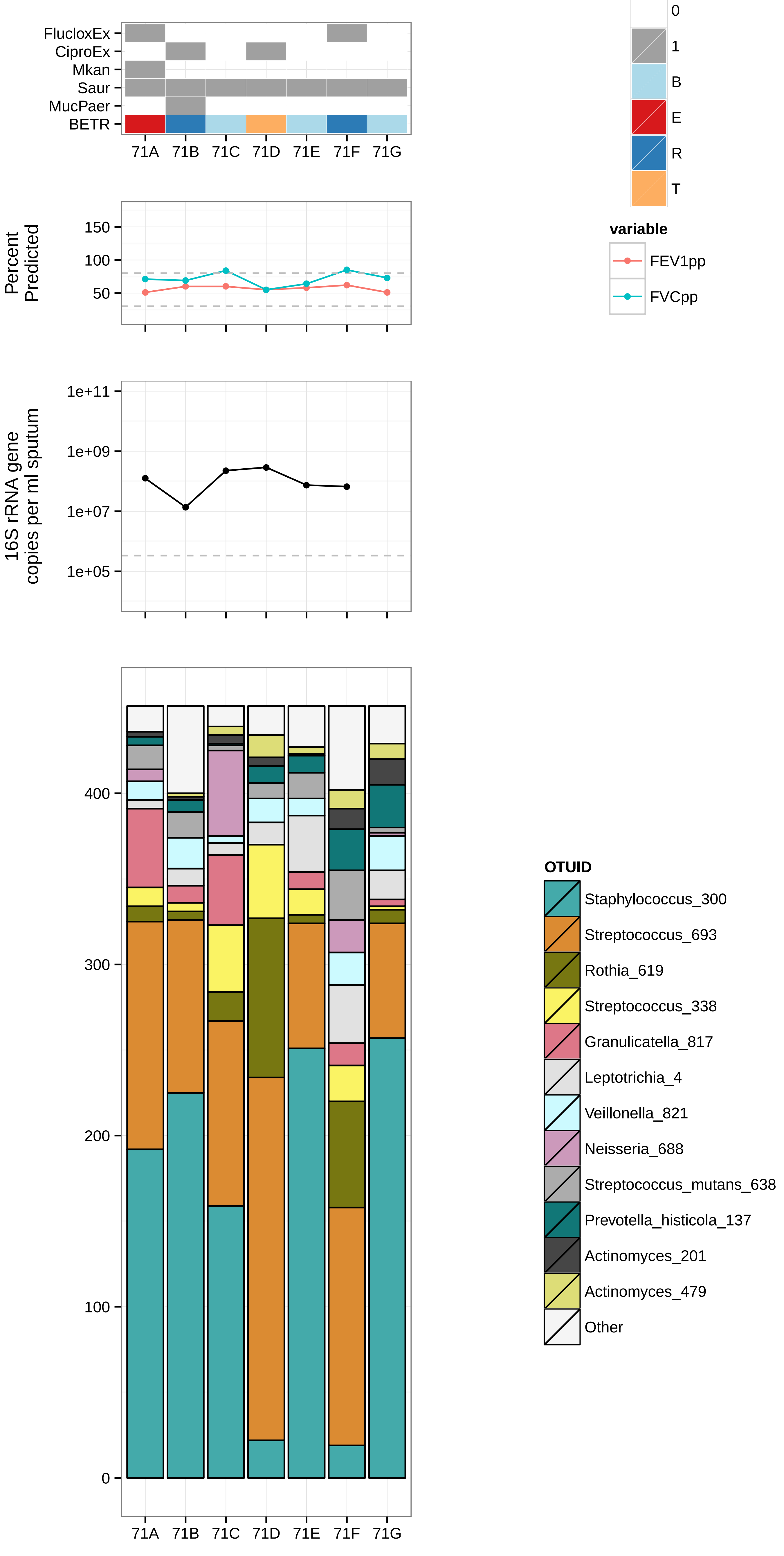
FlucloxEx
CiproEx
Mkan
Saur
MucPaer
BETR
71A
71B
71C
71D
71E
71F
71G
0
1
B
E
R
T
Percent Predicted
150
100
50
variable
FEV1pp
FVCpp
16S rRNA gene copies per ml sputum
1e+11
1e+09
1e+07
1e+05
400
300
200
100
0
OTUID
Staphylococcus_300
Streptococcus_693
Rothia_619
Streptococcus_338
Granulicatella_817
Leptotrichia_4
Veillonella_821
Neisseria_688
Streptococcus_mutans_638
Prevotella_histicola_137
Actinomyces_201
Actinomyces_479
Other

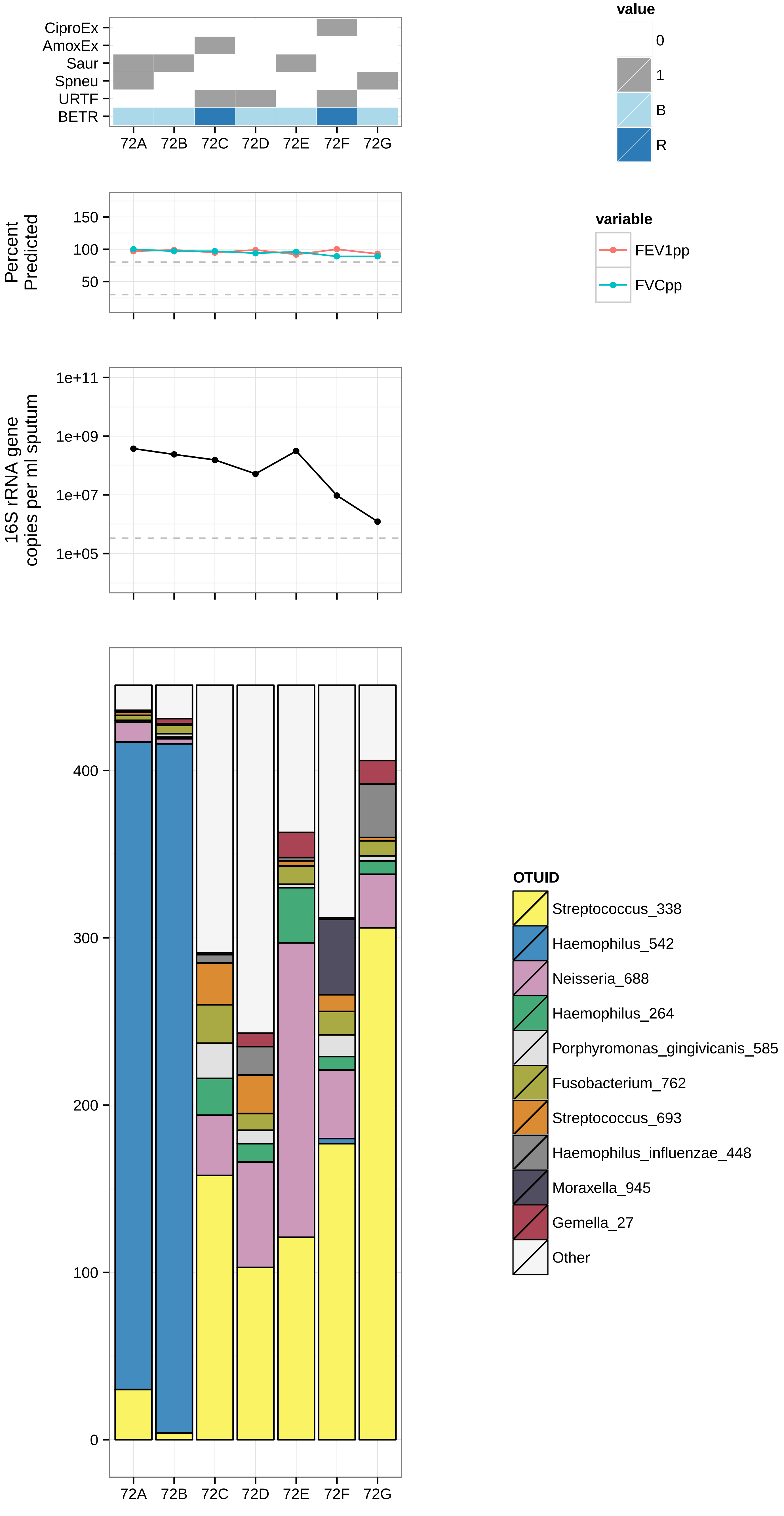

CiproEx
AmoxEx
Saur
Spneu
URTF
BETR
72A
72B
72C
72D
72E
72F
72G
value
0
1
B
R
Percent Predicted
150
100
50
variable
FEV1pp
FVCpp
16S rRNA gene copies per ml sputum
1e+11
1e+09
1e+07
1e+05
400
300
200
100
0
OTUID
Streptococcus_338
Haemophilus_542
Neisseria_688
Haemophilus_264
Porphyromonas_gingivicanis_585
Fusobacterium_762
Streptococcus_693
Haemophilus_influenzae_448
Moraxella_945
Gemella_27
Other

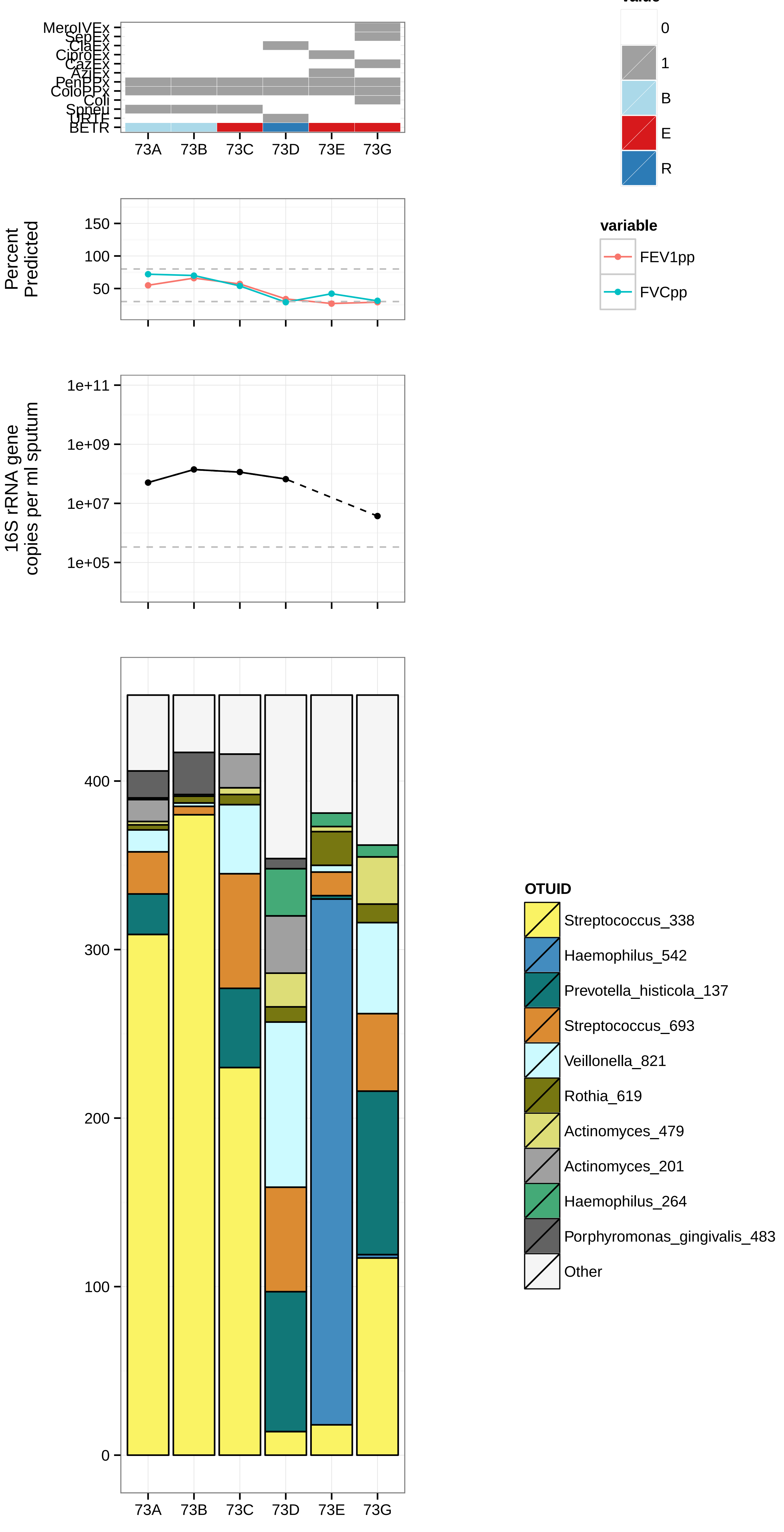

MeroIVEx
SepEx
ClaEx
CiproEx
CazEx
AziEx
PenPPx
ColoPPx
Coli
Spneu
URTI
BETR
73A
73B
73C
73D
73E
73G
value
0
1
B
E
R
Percent Predicted
150
100
50
variable
FEV1pp
FVCpp
16S rRNA gene copies per ml sputum
1e+11
1e+09
1e+07
1e+05
400
300
200
100
0
OTUID
Streptococcus_338
Haemophilus_542
Prevotella_histicola_137
Streptococcus_693
Veillonella_821
Rothia_619
Actinomyces_479
Actinomyces_201
Haemophilus_264
Porphyromonas_gingivalis_483
Other

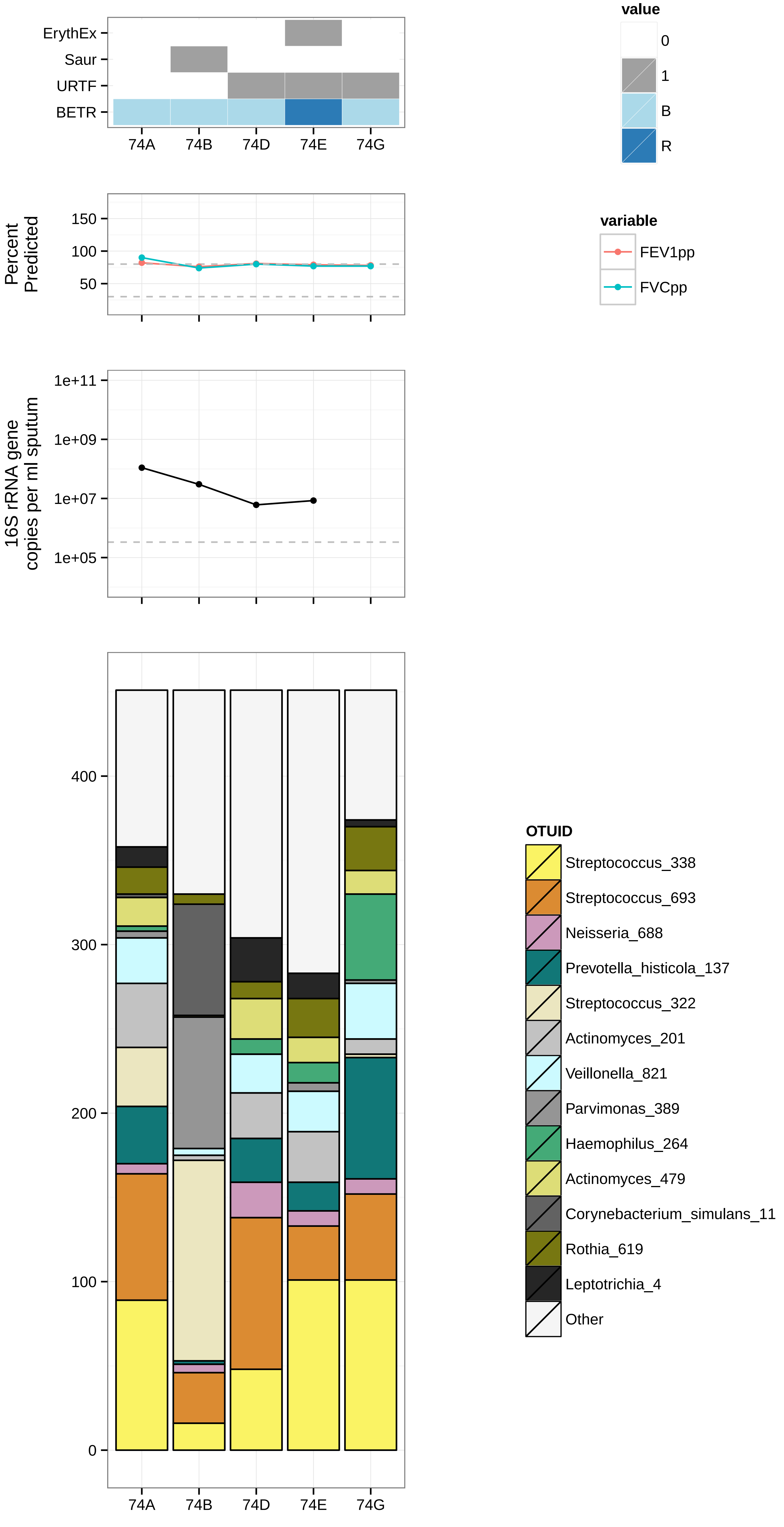
ErythEx
Saur
URTF
BETR
74A
74B
74D
74E
74G
value
0
1
B
R
Percent Predicted
150
100
50
variable
FEV1pp
FVCpp
16S rRNA gene copies per ml sputum
1e+11
1e+09
1e+07
1e+05
400
300
200
100
0
OTUID
Streptococcus_338
Streptococcus_693
Neisseria_688
Prevotella_histicola_137
Streptococcus_322
Actinomyces_201
Veillonella_821
Parvimonas_389
Haemophilus_264
Actinomyces_479
Corynebacterium_simulans_11
Rothia_619
Leptotrichia_4
Other

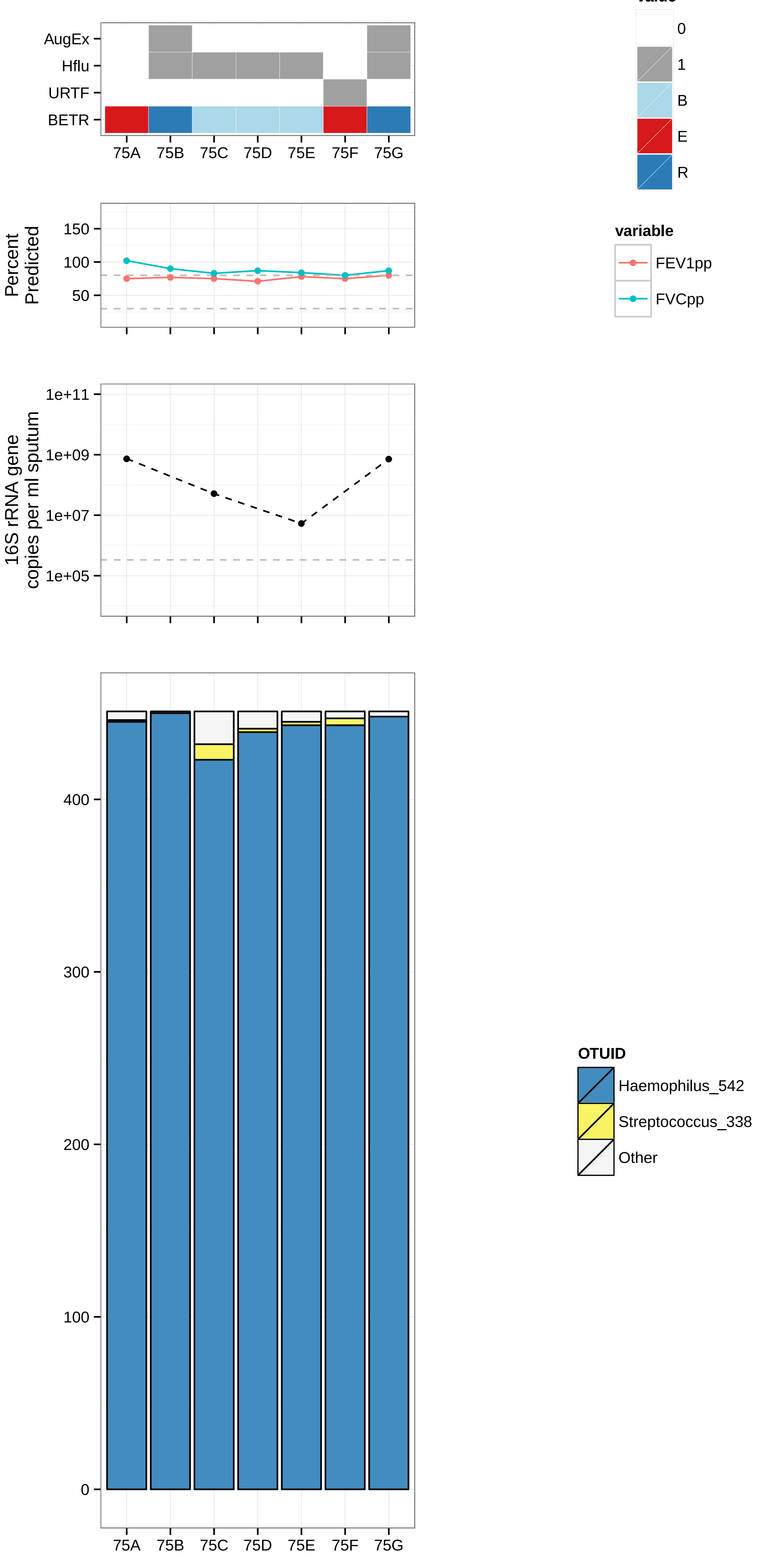
AugEx
Hflu
URTF
BETR
75A
75B
75C
75D
75E
75F
75G
0
1
B
E
R
Percent Predicted
150
100
50
variable
FEV1pp
FVCpp
16S rRNA gene copies per ml sputum
1e+11
1e+09
1e+07
1e+05
400
300
200
100
0
OTUID
Haemophilus_542
Streptococcus_338
Other

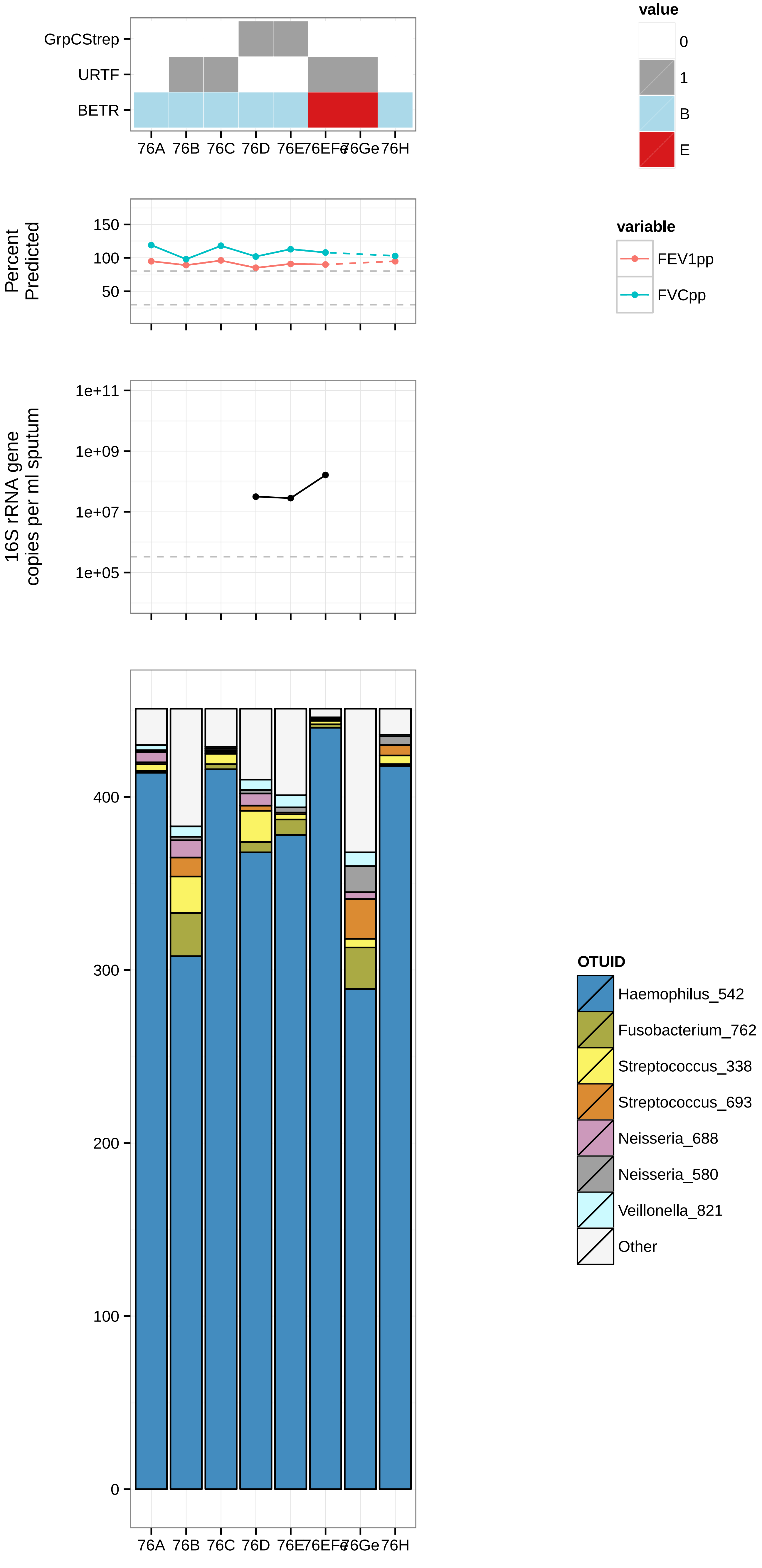

value
0
1
B
E
GrpCStrep
URTF
BETR
76A 76B 76C 76D 76E 76EFe 76Ge 76H
Percent Predicted
150
100
50
variable
FEV1pp
FVCpp
16S rRNA gene copies per ml sputum
1e+11
1e+09
1e+07
1e+05
400
300
200
100
0
OTUID
Haemophilus_542
Fusobacterium_762
Streptococcus_338
Streptococcus_693
Neisseria_688
Neisseria_580
Veillonella_821
Other
76A 76B 76C 76D 76E 76EFe 76Ge 76H

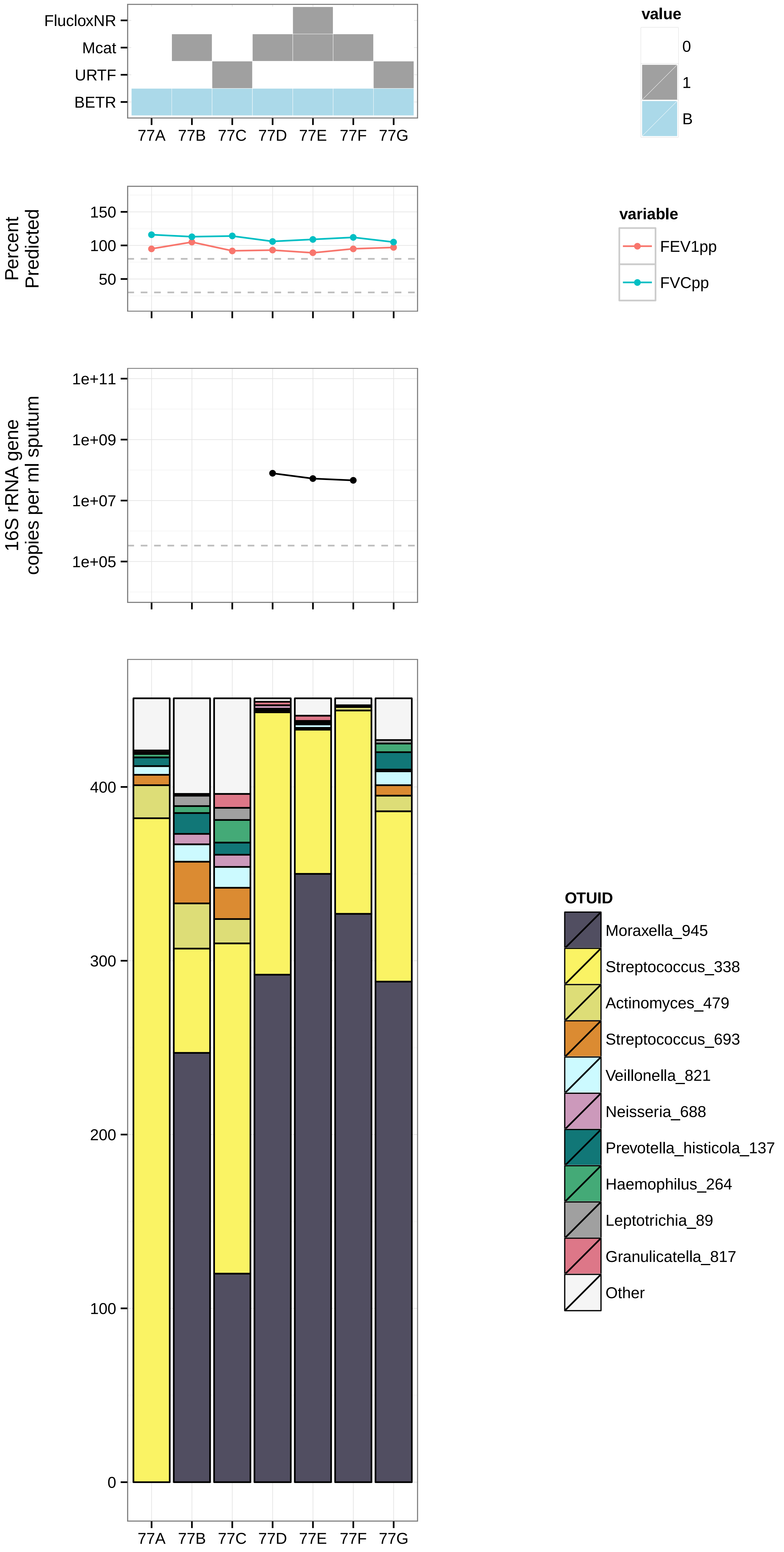

FlucloxNR
Mcat
URTF
BETR
77A
77B
77C
77D
77E
77F
77G
value
0
1
B
Percent Predicted
150
100
50
variable
FEV1pp
FVCpp
16S rRNA gene copies per ml sputum
1e+11
1e+09
1e+07
1e+05
400
300
200
100
0
OTUID
Moraxella_945
Streptococcus_338
Actinomyces_479
Streptococcus_693
Veillonella_821
Neisseria_688
Prevotella_histicola_137
Haemophilus_264
Leptotrichia_89
Granulicatella_817
Other

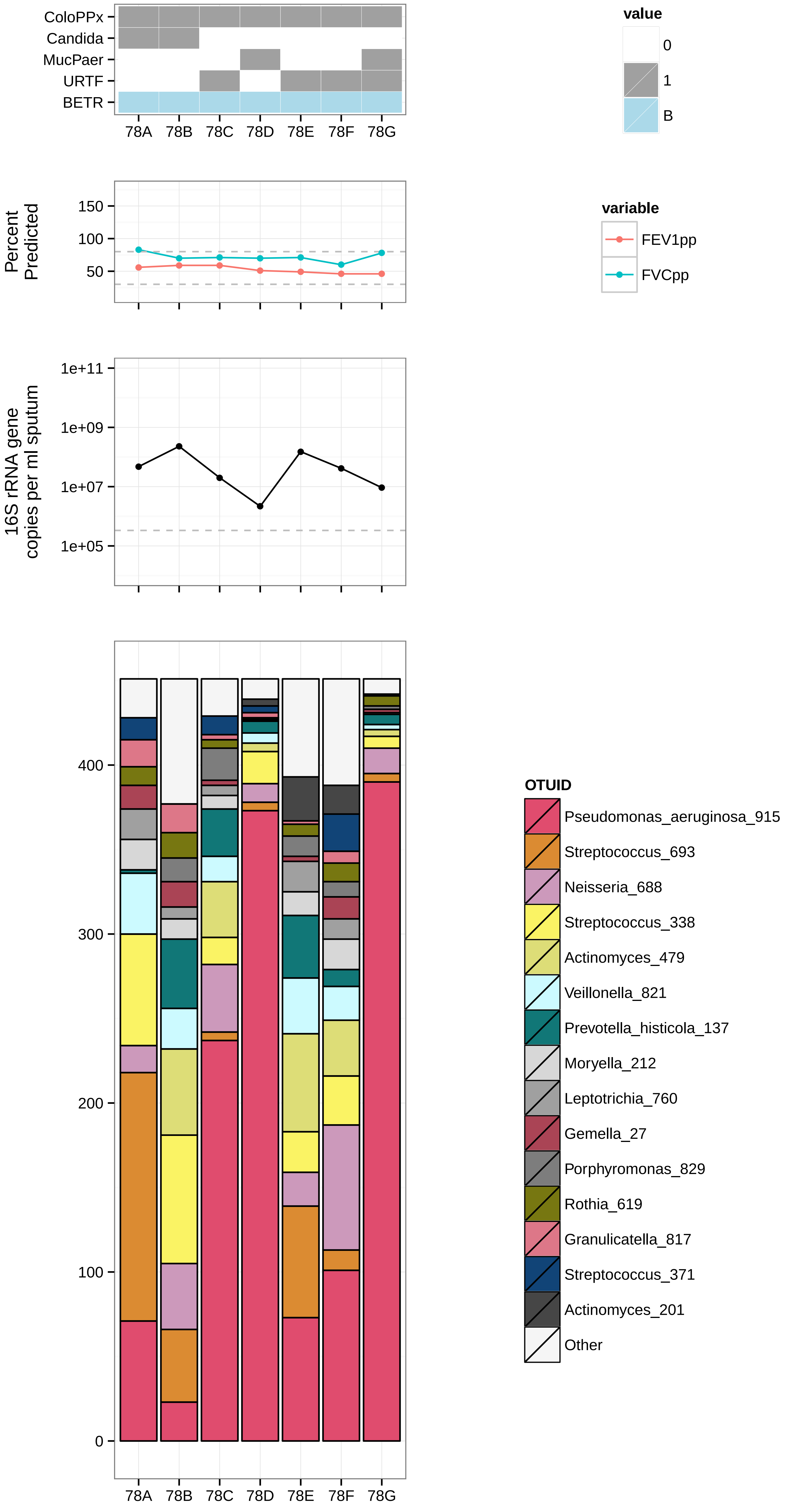

ColoPPx
Candida
MucPaer
URTF
BETR
78A 78B 78C 78D 78E 78F 78G
value
0
1
B
Percent Predicted
150
100
50
variable
FEV1pp
FVCpp
16S rRNA gene copies per ml sputum
1e+11
1e+09
1e+07
1e+05
400
300
200
100
0
OTUID
Pseudomonas_aeruginosa_915
Streptococcus_693
Neisseria_688
Streptococcus_338
Actinomyces_479
Veillonella_821
Prevotella_histicola_137
Moryella_212
Leptotrichia_760
Gemella_27
Porphyromonas_829
Rothia_619
Granulicatella_817
Streptococcus_371
Actinomyces_201
Other

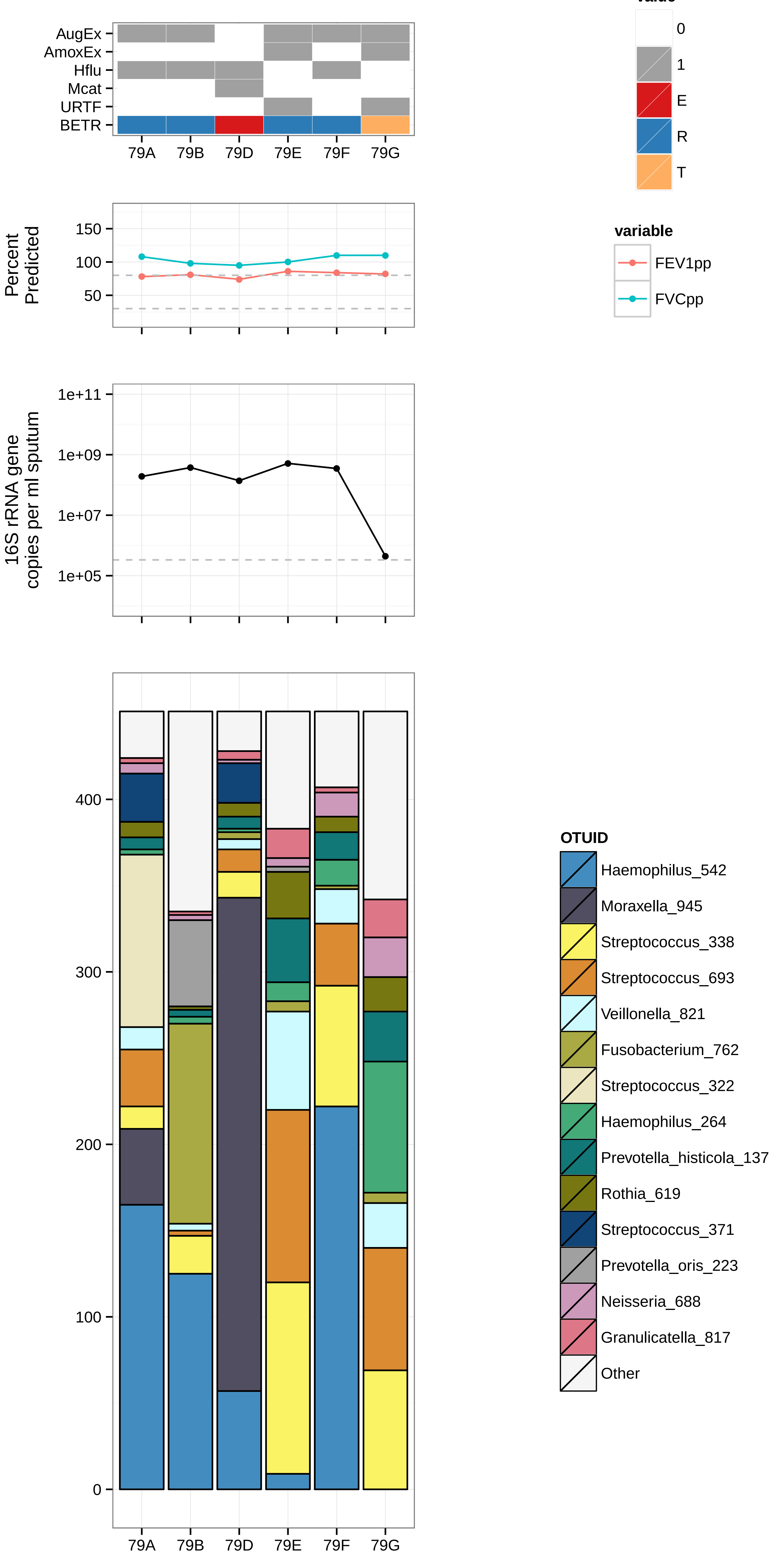
AugEx
AmoxEx
Hflu
Mcat
URTF
BETR
79A
79B
79D
79E
79F
79G
0
1
E
R
T
Percent Predicted
150
100
50
variable
FEV1pp
FVCpp
16S rRNA gene copies per ml sputum
1e+11
1e+09
1e+07
1e+05
400
300
200
100
0
OTUID
Haemophilus_542
Moraxella_945
Streptococcus_338
Streptococcus_693
Veillonella_821
Fusobacterium_762
Streptococcus_322
Haemophilus_264
Prevotella_histicola_137
Rothia_619
Streptococcus_371
Prevotella_oris_223
Neisseria_688
Granulicatella_817
Other

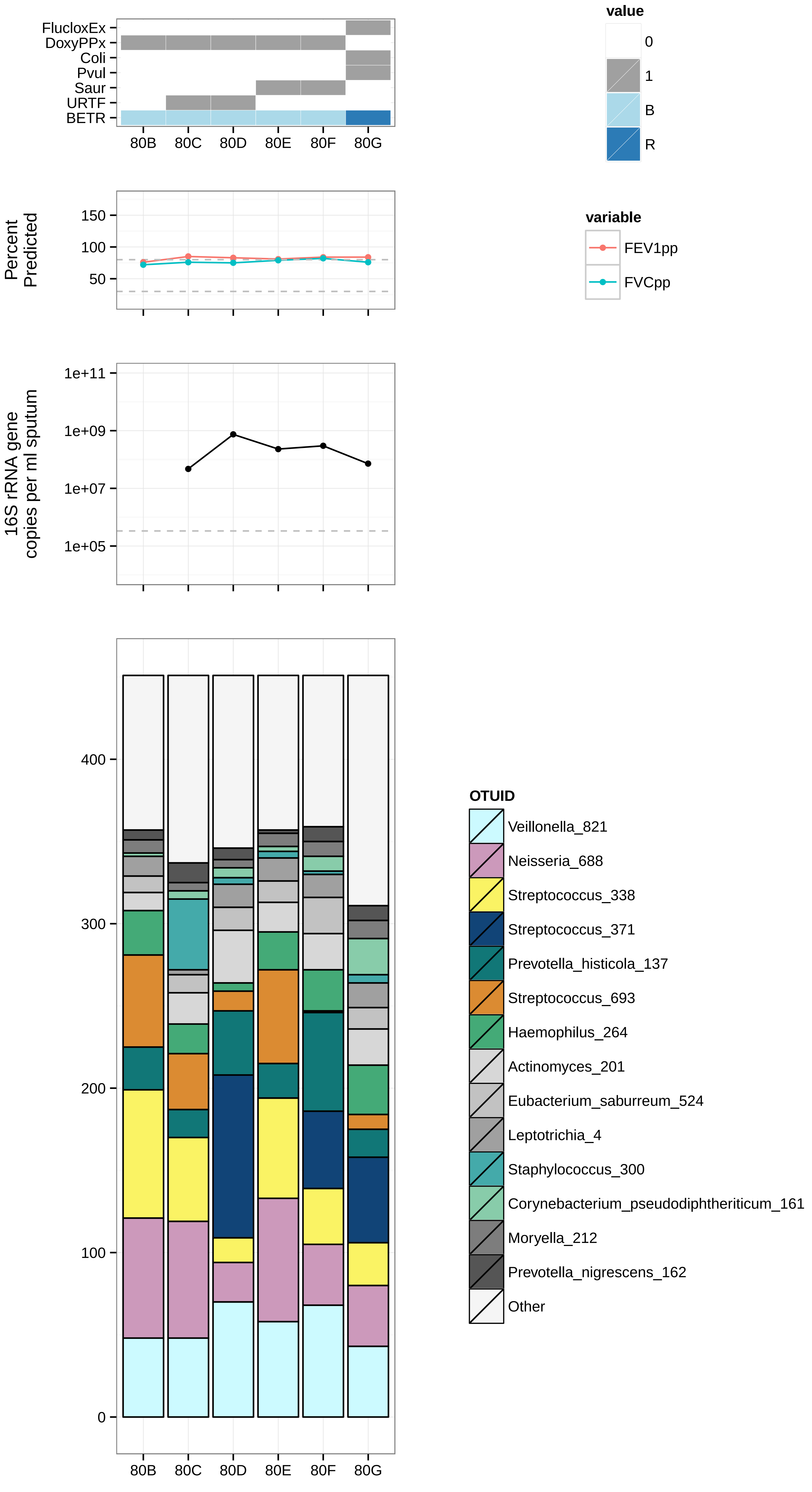

FlucloxEx
DoxyPPx
Coli
Pvul
Saur
URTF
BETR
80B
80C
80D
80E
80F
80G
value
0
1
B
R
Percent Predicted
150
100
50
variable
FEV1pp
FVCpp
16S rRNA gene copies per ml sputum
1e+11
1e+09
1e+07
1e+05
400
300
200
100
0
OTUID
Veillonella_821
Neisseria_688
Streptococcus_338
Streptococcus_371
Prevotella_histicola_137
Streptococcus_693
Haemophilus_264
Actinomyces_201
Eubacterium_saburreum_524
Leptotrichia_4
Staphylococcus_300
Corynebacterium_pseudodiphtheriticum_161
Moryella_212
Prevotella_nigrescens_162
Other

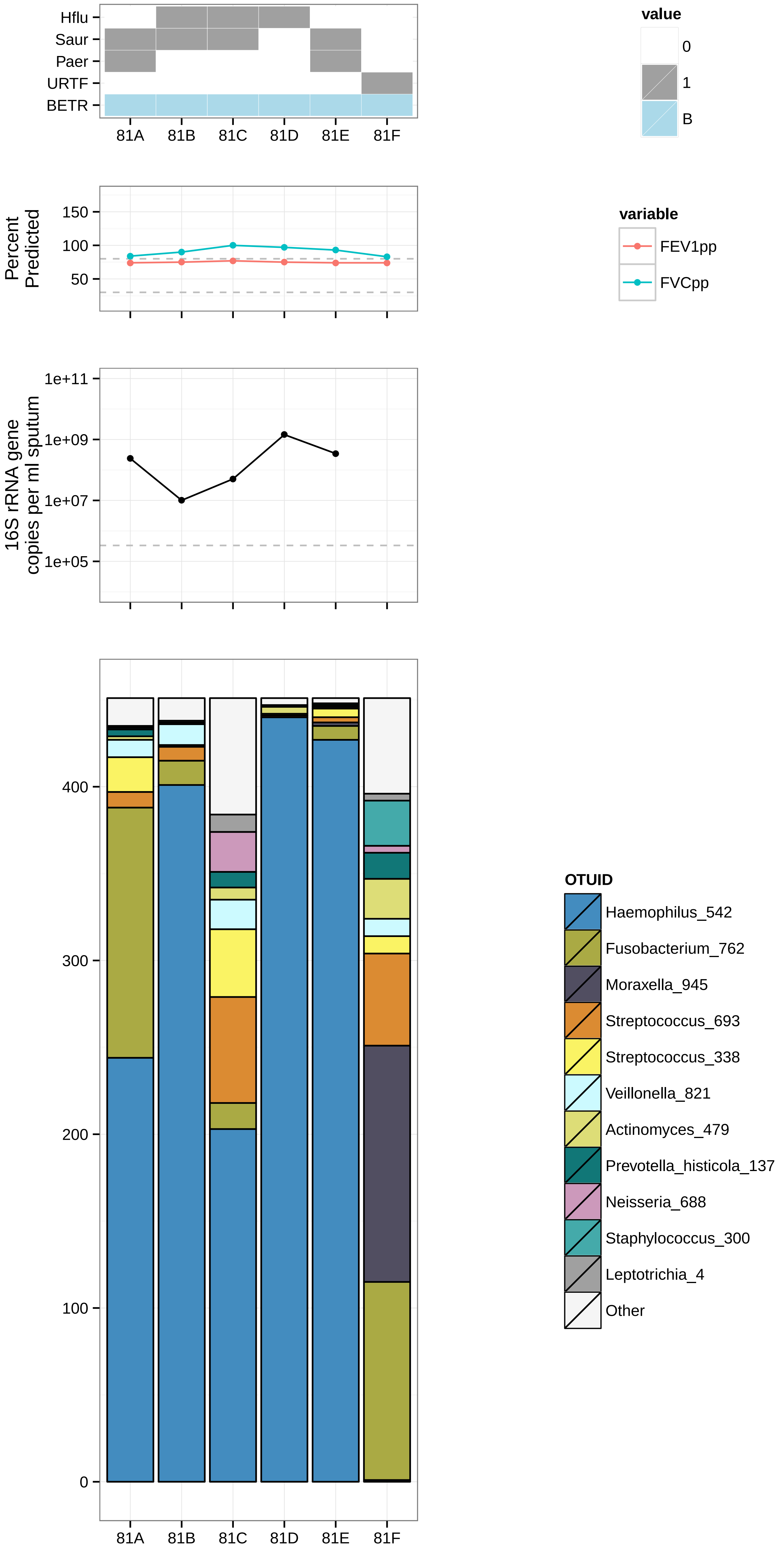

Hflu
Saur
Paer
URTF
BETR
81A
81B
81C
81D
81E
81F
value
0
1
B
Percent Predicted
150
100
50
variable
FEV1pp
FVCpp
16S rRNA gene copies per ml sputum
1e+11
1e+09
1e+07
1e+05
400
300
200
100
0
OTUID
Haemophilus_542
Fusobacterium_762
Moraxella_945
Streptococcus_693
Streptococcus_338
Veillonella_821
Actinomyces_479
Prevotella_histicola_137
Neisseria_688
Staphylococcus_300
Leptotrichia_4
Other

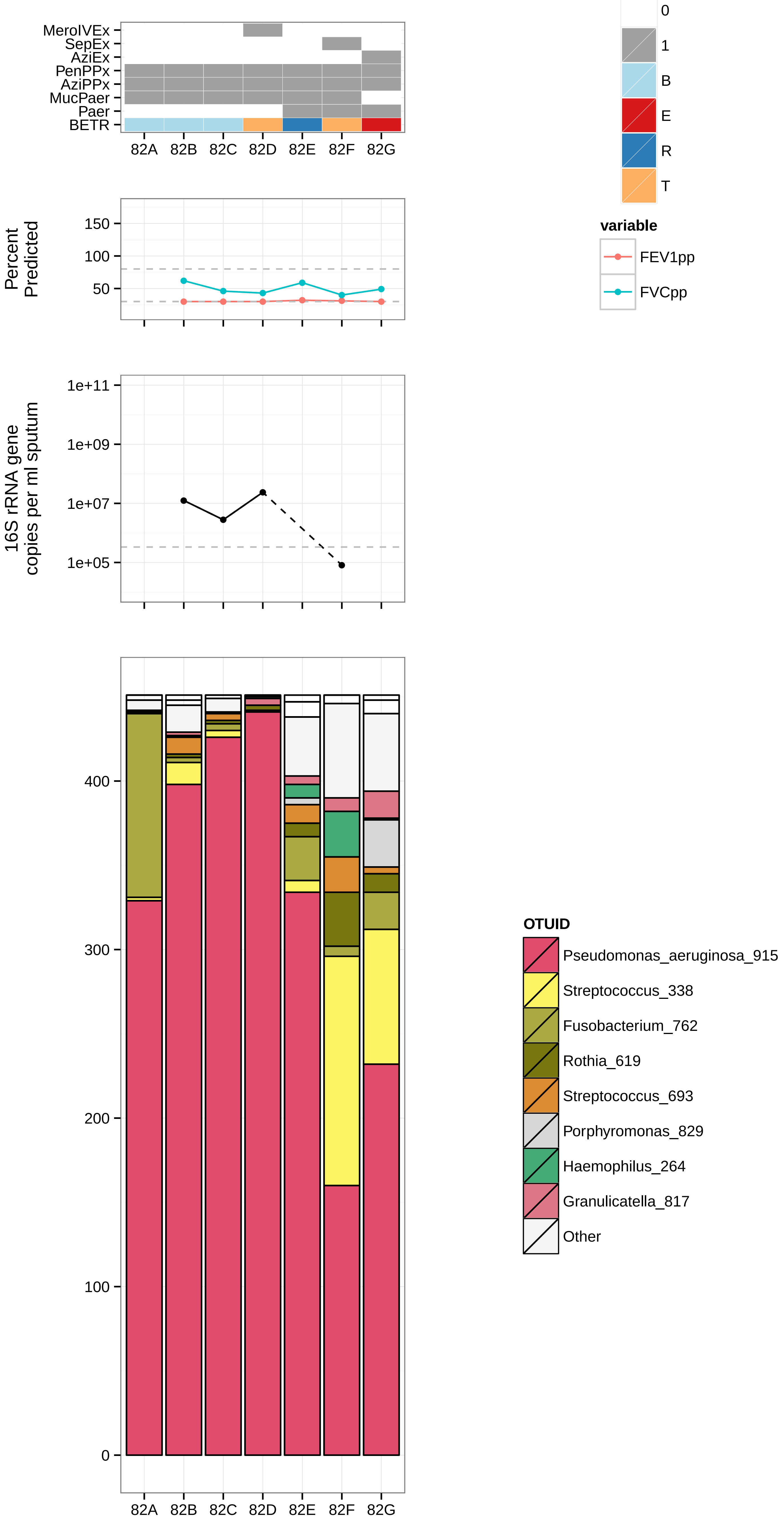

MeroIVEx
SepEx
AziEx
PenPPx
AziPPx
MucPaer
Paer
BETR
82A
82B
82C
82D
82E
82F
82G
0
1
B
E
R
T
Percent Predicted
150
100
50
variable
FEV1pp
FVCpp
16S rRNA gene copies per ml sputum
1e+11
1e+09
1e+07
1e+05
400
300
200
100
0
OTUID
Pseudomonas_aeruginosa_915
Streptococcus_338
Fusobacterium_762
Rothia_619
Streptococcus_693
Porphyromonas_829
Haemophilus_264
Granulicatella_817
Other

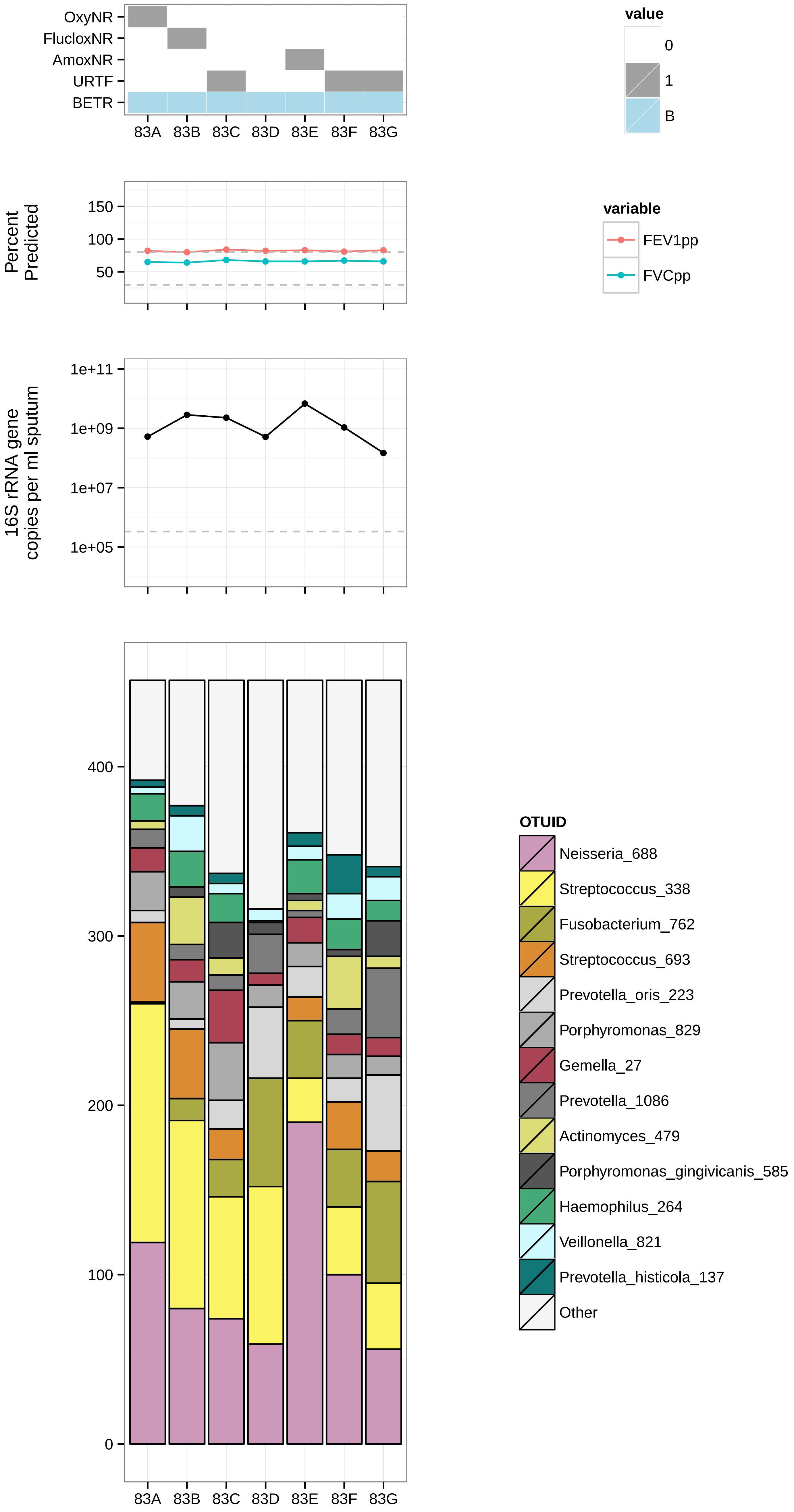
OxyNR
FlucloxNR
AmoxNR
URTF
BETR
83A
83B
83C
83D
83E
83F
83G
value
0
1
B
Percent Predicted
150
100
50
variable
FEV1pp
FVCpp
16S rRNA gene copies per ml sputum
1e+11
1e+09
1e+07
1e+05
400
300
200
100
0
OTUID
Neisseria_688
Streptococcus_338
Fusobacterium_762
Streptococcus_693
Prevotella_oris_223
Porphyromonas_829
Gemella_27
Prevotella_1086
Actinomyces_479
Porphyromonas_gingivicanis_585
Haemophilus_264
Veillonella_821
Prevotella_histicola_137
Other

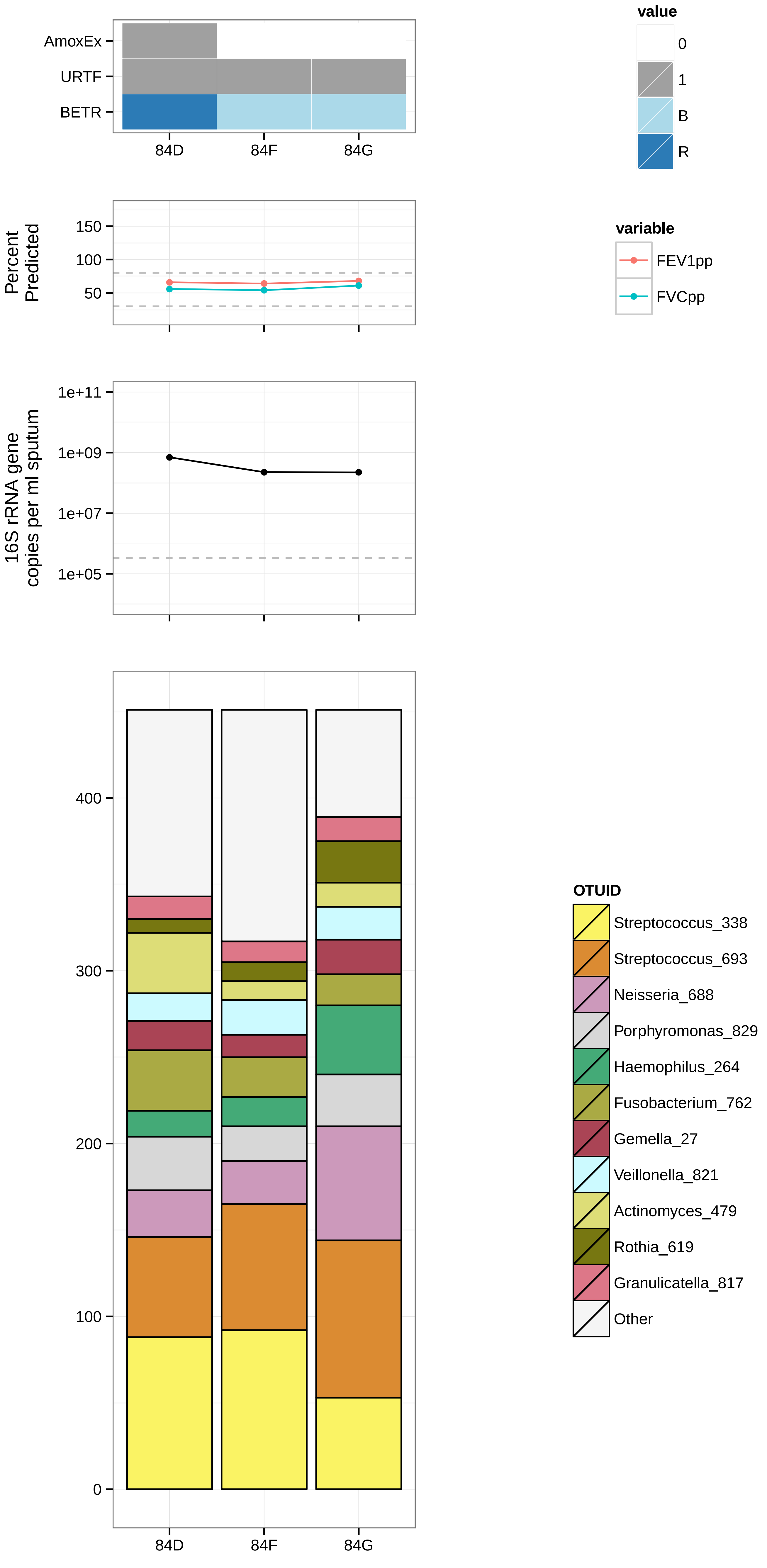
AmoxEx
URTF
BETR
84D
84F
84G
value
0
1
B
R
Percent Predicted
150
100
50
variable
FEV1pp
FVCpp
16S rRNA gene copies per ml sputum
1e+11
1e+09
1e+07
1e+05
400
300
200
100
0
OTUID
Streptococcus_338
Streptococcus_693
Neisseria_688
Porphyromonas_829
Haemophilus_264
Fusobacterium_762
Gemella_27
Veillonella_821
Actinomyces_479
Rothia_619
Granulicatella_817
Other

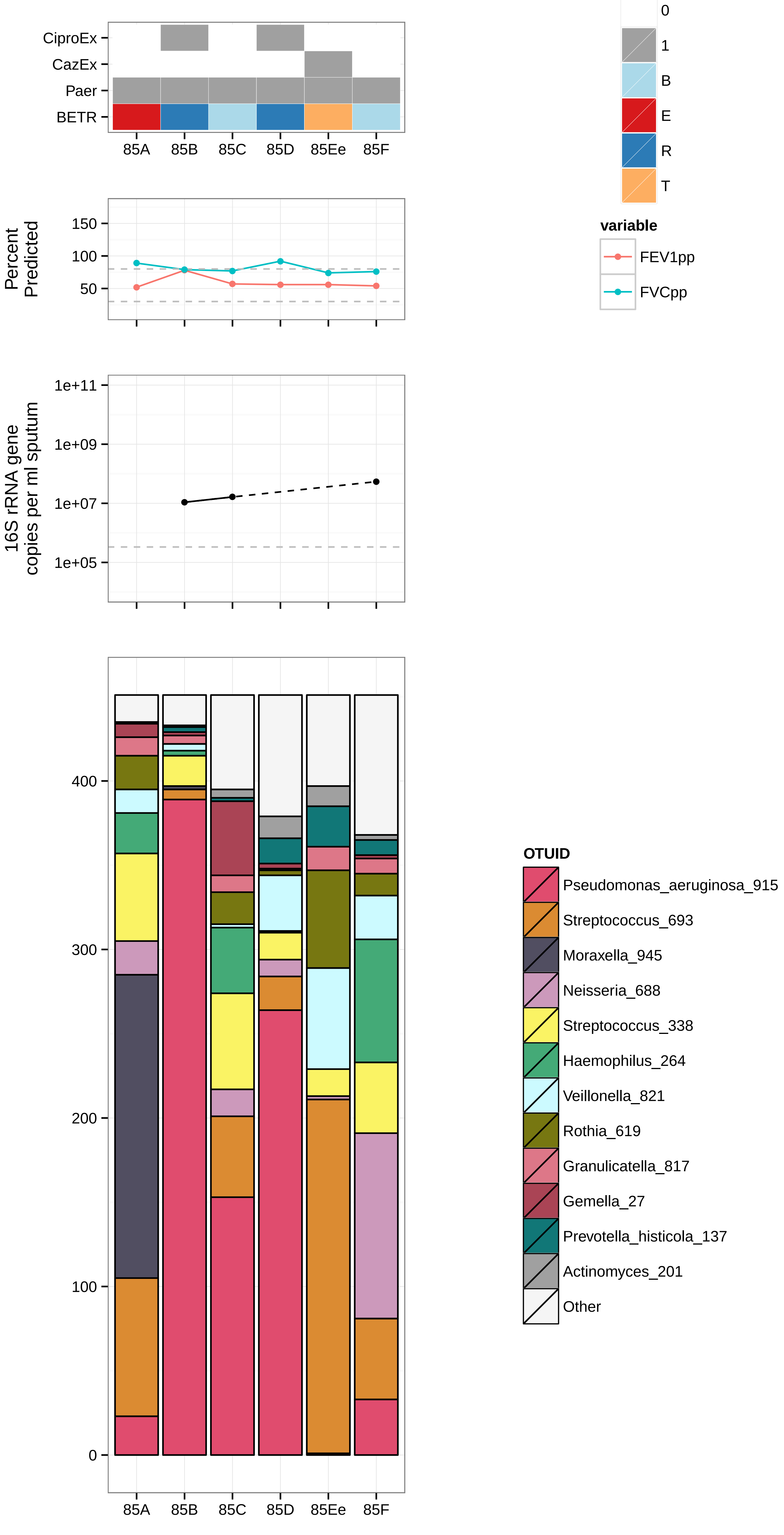

CiproEx
CazEx
Paer
BETR
85A
85B
85C
85D
85Ee
85F
0
1
B
E
R
T
Percent Predicted
150
100
50
variable
FEV1pp
FVCpp
16S rRNA gene copies per ml sputum
1e+11
1e+09
1e+07
1e+05
400
300
200
100
0
OTUID
Pseudomonas_aeruginosa_915
Streptococcus_693
Moraxella_945
Neisseria_688
Streptococcus_338
Haemophilus_264
Veillonella_821
Rothia_619
Granulicatella_817
Gemella_27
Prevotella_histicola_137
Actinomyces_201
Other
